# Extending the *Schizosaccharomyces pombe* Molecular Genetic Toolbox

**DOI:** 10.1371/journal.pone.0097683

**Published:** 2014-05-21

**Authors:** Dorota Fennessy, Agnes Grallert, Andrea Krapp, Adisa Cokoja, Alan J. Bridge, Janni Petersen, Avinash Patel, Victor A. Tallada, Elvan Boke, Ben Hodgson, Viesturs Simanis, Iain M. Hagan

**Affiliations:** 1 Cell Division Group, Cancer Research UK Manchester Institute, University of Manchester, Manchester, United Kingdom; 2 Swiss Institute for Experimental Cancer Research, École polytechnique fédérale de Lausanne, Lausanne, Switzerland; Cancer Research UK London Research Institute, United Kingdom

## Abstract

Targeted alteration of the genome lies at the heart of the exploitation of *S. pombe* as a model system. The rate of analysis is often determined by the efficiency with which a target locus can be manipulated. For most loci this is not a problem, however for some loci, such as *fin1* ^+^, rates of gene targeting below 5% can limit the scope and scale of manipulations that are feasible within a reasonable time frame. We now describe a simple modification of transformation procedure for directing integration of genomic sequences that leads to a 5-fold increase in the transformation efficiency when antibiotic based dominant selection markers are used. We also show that removal of the *pku70* ^+^ and *pku80* ^+^ genes, which encode DNA end binding proteins required for the non-homologous end joining DNA repair pathway, increases the efficiency of gene targeting at *fin1* ^+^ to around 75–80% (a 16-fold increase). We describe how a *natMX6/rpl42* ^+^ cassette can be used for positive and negative selection for integration at a targeted locus. To facilitate the evaluation of the impact of a series of mutations on the function of a gene of interest we have generated three vector series that rely upon different selectable markers to direct the expression of tagged/untagged molecules from distinct genomic integration sites. pINTL and pINTK vectors use *ura4* ^+^ selection to direct disruptive integration of *leu1* ^+^ and *lys1* ^+^ respectively, while pINTH vectors exploit nourseothricin resistance to detect the targeted disruption of a hygromycin B resistance conferring *hphMX6* cassette that has been integrated on chromosome III. Finally, we have generated a series of multi-copy expression vectors that use resistance to nourseothricin or kanamycin/G418 to select for propagation in prototrophic hosts. Collectively these protocol modifications and vectors extend the versatility of this key model system.

## Introduction

The genetic malleability of the fission yeast *S. pombe* has helped it to maintain a prominent position alongside the more extensively exploited budding yeast *Saccharomyces cerevisiae,* as a powerful model system for the characterisation of the basic facets of eukaryotic cell and molecular biology. This malleability is based upon an extensive repertoire of classical and molecular genetic techniques [1], [2], [3]. As in budding yeast these techniques were initially based upon the exploitation of key auxotrophic markers.

Classical genetic analysis the adenine biosynthesis pathway in *S. pombe* highlighted the utility of the colony-colour change resulting from the accumulation of P-ribosylaminoimidazole in *ade6* mutants that is then oxidised to a red pigment [4]. The ability to use this red pigmentation as a reporter for Ade6 function made this locus a major focus for studies of core genetic principles. These studies led to the development of a number of useful genetic tools including *ade6.M210/ade6.M216* hetero-allelic complementation for the selection and maintenance of diploid strains [5] and the use of the *sup3.5* opal suppressor tRNA^ser^ mutation as a marker for selection in an *ade6.704* mutant background [6], [7], [8]. Cross species complementation of *S. pombe leu1* mutations with the *S. cereviaisae LEU2* ^+^ gene was initially used to apply existing budding yeast technology to fission yeast [9], but remains a widely used selectable marker to this day because the lack of homology to sequences in the *S. pombe* genome means that it does not direct integration into a specific genomic site. However, when used as a marker to select for site specific integration, multiple integration events can occur [10], suggesting either that the heterologous expression of the *LEU2* ^+^ gene is barely sufficient for growth at low copy number or that the budding yeast enzyme is less attuned to fission yeast physiology than the native 3-isopropyl malate dehydrogenase enzyme, Leu1. Transposition of the lessons learnt from the exploitation of the budding yeast ornithine decarboxylase *URA3* ^+^ gene for positive and negative selection [11] led to the deletion of the *ura4* ^+^ gene from *S. pombe* to generate the *ura4.d18* allele that is so widely used in the field today [12] with many *ura4* ^+^ based vectors [13], [14], [15], [16]. Continued developments are considerably expanding the array of available auxotrophy-complementing markers to include: *ade7, his1, his2, his3, his5, arg3, arg12, lys1, lys2* and *tyr1* [17], [18], [19], [20], [21], [22], [23], [24]. However, *his3* ^+^, *LEU2* ^+^and *ura4* ^+^ remain the most widely-used markers for selection of multi-copy vectors in common use. Integration vectors that target a particular heterologous locus have been less extensively developed, however the pDUAL series and pJK148 vectors are used widely as they exploit recombination to convert the leucine auxotrophy of *leu1.32* to leucine prototrophy to select integration at the *leu1* locus [25], [26], [27]. The pJK210 uses a similar rescue of *ura4.294* to target integration at the *ura4* locus [25].

While these auxotrophic selection markers offer powerful tools, they also create the need to introduce an increasingly complex array of background markers into a strain of interest. Not only is this time consuming but many combinations of deficiencies in amino acid provision compromise a host strain’s fitness on certain media, which may complicate the interpretation of the phenotype arising from the mutation of interest. Furthermore, the sensitivity of the broadly acting TOR signalling network to addition of leucine to the medium [28] indicates that provision of amino acids demanded by the use of auxotrophic markers and perhaps the auxotrophic markers themselves are not merely passive players in cellular homeostasis, but can influence the control networks that impinge upon diverse processes from metabolism, through cell cycle control, sexual differentiation, and the actin cytoskeleton. Thus, controlling the genetic context within which the consequences of particular mutations are studied in prototrophs not only accelerates the rate of analysis, but avoids both anticipated and unforeseen complications arising from interplay between pathways.

Following the highly successful exploitation of antibiotic resistance genes as dominant selectable markers for PCR based tagging and deletion approaches in the budding yeast *S. cerevisiae* [29], [30], [31], [32], [33], [34], the technology has been adapted for use in a variety of fungi including *S. pombe*. Genes conferring resistance to kanamycin/G418, hygromycin B, phleomycin/bleomycin and nourseothricin/ClonNat are highly effective dominant markers in fission yeast [35], [36], [37], [38]. They have been extensively exploited in an increasing array of “PCR tagging vectors” in which oligo-nucleotides, that fuse vector sequences to short stretches of homology to the target locus, are used to amplify cassettes that will place “tags” and markers of choice in particular genomic contexts following targeted recombination into the host genome [29], [30], [38], [39], [40], [41], [42], [43], [44], [45]. While this approach is very powerful, it still faces the challenge that the number of manipulations is limited by the range of markers available. However, this problem can be circumvented by flanking the marker with loxP sites so that it can be excised from the genome following integration by the induction of Cre recombinase [46]. Host strains can then be sequentially modified with the same selectable marker, irrespective of previous manipulations.

Although attempts to define the extent of the problem have proved challenging [25], [47], [48], many anecdotal accounts suggest that the efficiency of targeting different loci by PCR tagging is variable; some loci can be targeted with very high efficiency while others only poorly, or not at all. It has been suggested that illegitimate recombination poses a major issue in these cases. In such circumstances the problem may be resolved by extending the region of homology with the genome can enhance the efficiency of targeting. In many other fungi deletion of the genes encoding the DNA end recognition proteins that are required for non-homologous end joining (NHEJ), Ku70 and Ku80 [49] greatly increases targeting efficiencies [50], [51], [52], [53], [54], [55], [56].

We now show that the removal of either the Ku70 or Ku80 homologues from *S. pombe* (Pku70 and Pku80 respectively) increases targeting efficiency at the *fin1* ^+^ locus from 5% to 80% (16-fold increase). A modification to transformation procedures enhances transformation frequencies by a further 5–8 fold when selecting for antibiotic resistance markers. We show that a PCR cassette that combines the cycloheximide sensitivity of *rpl42* ^+^ in an *rpl42.sP56Q* background [57] with the nourseothricin resistance conferred by *natMX6* [58] offers a robust and cheaper alternative to positive and negative selection cycles with *ura4* ^+^ and 5 fluoro-orotic acid (FOA) [12]. We describe two complementary integration vector series that exploit disruption of an auxotrophic marker with a second auxotrophic marker to direct the regulated expression of tagged or untagged molecules from a reproducible genome context. A further set of integration vectors exploits antibiotic resistance markers to direct the integration of both tagged and untagged expression cassettes into a site on chromosome III. We have also switched the markers in our multi-copy pREP41 based tagging/expression series [16] to generate vectors that exploit *natMX6* or *kanMX6* as a selectable marker.

## Materials and Methods

### Strain Growth, Selection and Maintenance

*E. coli* strain *DH5*α was used to propagate plasmids in standard LB medium. The *S. pombe leu1.32 his2 *h+(IH147) and 972h- (IH5974) strains were grown by standard procedures [2]. The following stock solutions of 100 mg ml^−1^ Geneticin (MP Biomedicals, 158782), Hygromycin B (Calbiochem, 400050), Nourseothricin/ClonNat (Werner BioAgents, 96736-11-7), Phleomycin (Sigma, P9564), and Cyclohexamide (Sigma, C1988) were added to generate final concentrations of 100 µg/ml in the growth medium where appropriate.

### Transformation of *S. pombe*

Cells were grown to mid-log phase in YES (4×10^6^ cells/ml). After harvesting cells were washed with H_2_O, 0.1 M Lithium Acetate (pH 4.9) and re-suspended in 0.1 M Lithium Acetate (pH 4.9) at 10^9^ cells ml^−1^. After 1 h incubation at 25°C 1–5 µg DNA and 290 µl of 50% PEG4000 (freshly made in sterile 0.1 M Lithium Acetate, pH 4.9 for each transformation) was added to 100 µl cell-suspension. After 1h incubation at 25°C and 15 min heat shock at 43°C, cells were harvested, washed with H_2_O and re-suspended in MSL-N or spread to YES.

### Molecular Genetics

#### Generating pINTL vectors

A 686 bp fragment of pUC19, including the MCS and *Nde*I site, were removed by Phusion mediated deletion (New England Biolabs) using oligo nucleotides BH1 and BH2, creating a NotI site. A 2.32 kb fragment extending from −456 to +752 of the *S. pombe leu1* ^+^ gene was amplified as a *Not*I fragment using oligo-nucleotides BH3 and BH4 and cloned into the modified pUC19 vector to create pINT1. An 1.78 kb fragment extending from –516 to +1186 of the *S. pombe ura4* ^+^ gene was amplified from pURA4 [12] using oligonucleotides BH5 and BH6 to flank the *ura4* ^+^ sequences with 5′ *Pst*I site and 3′ *Sac*I sites. Oligos BH7 and BH8 were used to amplify pINT1, which was then used as recipient for the amplified *ura4* ^+^ fragment within the *leu1* ^+^ open reading frame by Gibson-mediated integration (New England Biolabs) to generate pINTLA that can act as recipient for any *Pst*I/*Sac*I fragment containing promoter-insert-terminator (Figure S1). The remaining plasmids in the pINTL series were generated by cloning the appropriate *Pst*I–*Sac*I fragment from a relevant pREP tagging vector [16]. pINTL41PkN was generated by insertion of the *Pst*I/*Sac*I fragment of pREP41PkN into pINTLA followed by the *Sac*I/*Sac*I fragment from pREP41PkN. Full sequences of the pINTL vectors are presented in Figure S1.

#### Generating pINTK vectors

The *lys1* 5′ region was amplified (VS642/VS644) to introduce *HindIII NotI* sites at one end and *PstI* site at the other end. The *lys1* 3′region was amplified (VS645/VS646) to introduce a *KpnI* site at one end and *EcoRI NotI* sites at the other end. Both fragments were cloned *HindIII - PstI* and *KpnI – EcoRI*, respectively into pGEM3. The *loxP–ura4* cassette was generated by PCR amplification of the *ura4^+^* gene (VS647/VS648) to introduce *KpnI* site and a LoxP site on one end and *SmaI SacI* sites with the LoxP site on the other end. This fragment was then cloned as a *KpnI – SmaI* fragment into the *lys1^+^* containing vector to generate pINTK, pINTK81, pINTK41 and pINTK1 by cloning the *nmt* promoters from pREP81, pREP41 and pREP1 respectively as *PstI – BamHI* fragments into pINTK. GFP, CFP and YFP tags were amplified to introduce a *BamHI* site at the 5′end and *NheI SmaI SacI* sites at the 3′end. The tags were then cloned *BamHI – SacI* into the pINTK81/41/1. The 6His tag was cloned as a *BamHI – NheI* fragment generated after annealing of complementary oligonucleotides (VS1482/VS1483) into the pINTK1GFP to generate pINTK1-6His. The sequence of the pINTK vector is presented in Figure S2.

#### Generating pINTH vectors

The first step in the generation of the pINTH vector was Quickchange (Stragene) silent mutagenesis to remove the *PstI* and *NdeI* sites from *hphMX6* (CTGCAG>CTGCAA and CATATG>CATTTG, respectively) and a *XmaI/SmaI* site from *natMX6* (CCCGGG>CCCAGG). The following fragments were amplified using the indicated primers to introduce restriction sites at their termini before being cloned into the vector ZeroBluntTOPO (Invitrogen): one 0.85 kb half of *hphMX6* flanked with *PstI* and *NotI HindIII* (primers DF1 and DF2); the remaining 0.85 kb fragment flanked by *EcoRI* and *EcoRI NotI* (primers DF3 and DF4); *natMX6* flanked with *SacI* and *EcoRI* (primers DF5 and DF6). The *SacI* – *EcoRI natMX6* fragment was inserted into pUC19 followed by the *EcoRI* and *Pst1-HindIII hphMX6* fragments to generate pINT*. pINT* was digested *PfoI* and *KpnI*, end filled with Klenow polymerase (New England Biolabs) to remove a 189 bp fragment and re-ligated to remove the *Nde*I site of pUC19 to generate pINT**. *PstI SacI* sequences containing the *nmt* promoter - cloning site/tag - *nmt* teminator cassettes from pREP1, pREP41 and pREP81 based plasmids were then inserted between *PstI SacI* sites in pINT** to generate the vector series. Because the multi-cloning site of pREP41PkN and pREP81PkN contains a SacI site, the pINTH41PkN and pINTH81PkN were generated by sequential insertion of the appropriate SacI and PstI-SacI fragments from pREP41PkN and pREP81PkN respectively. pINT* was digested with *NdeI,* end filled with Klenow polymerase (New England Biolabs) and re-ligated to generate pINTHA. Full sequences of the pINTH vectors are presented in Figure S3.

#### Generating pREPN and pREPK vectors

To generate the pREPN vectors, the *Sma*I sites of the *natMX6* gene in *natMX6* cassette were destroyed (the NdeI site in pFA6anatMX6 is outside of the cassette). The *natMX6* gene was amplified with the oligonucleotides AG1and AG2 that had 20 nucleotides homology to the ends of the *natMX6* cassette and 80 bp homology with the sequences adjacent to the *LEU2* ^+^ integration site of pREP1. 5 µg of the *natMX6* fragment was transformed alongside 1 µg of the appropriate *LEU2* ^+^ based pREP vector that had been linearised by digestion with *KpnI* that cut inside the *LEU2* ^+^ marker gene of the relevant vector in host IH147. Plasmids were isolated from two antibiotic resistant leucine auxotrophic colonies with the DNA isolation kit (Flowgen), before 5 µl was transformed into *DH5*α. Restriction mapping and sequencing of DNA from two transformants identified the desired vector. The pREPK series were made in the same way as pREPN, but the co-transformation used a KanMX6 fragment amplified from pFA6a-kanMX6 [40].

#### Biochemistry

Generation of cell extracts and western blotting of these extracts was as described previously [59].

## Results

### A *natMX6/rpl42* Cassette for Positive and Negative Selection

**Figure 1:**
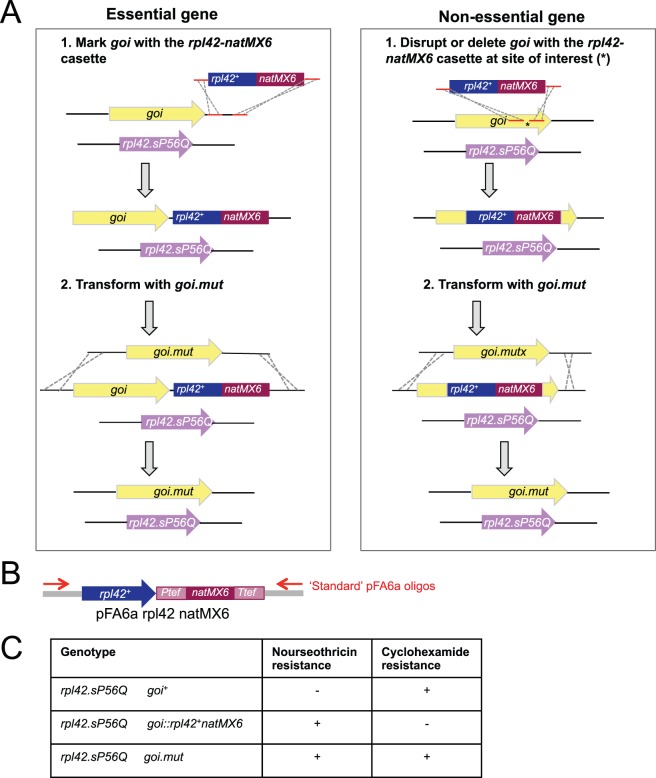
Manipulating native loci with an *rpl42^+^/natMX6* cassette. A) Approaches used for targeted mutagenesis. B) The structure of the pFA6arpl42natMX6 plasmid. C) The phenotype switches arising from the progression through the indicated genotypes.

To study the significance of phosphorylation events in the timing and execution of cell division we mutate candidate sites at endogenous loci [60], [61], [62]. We first integrate a marker at the gene of interest (*goi* ^+^) before transforming this new host strain with a fragment whose homology to the genome extends beyond either side of the integration site. As this fragment harbours a *goi* mutation, positively selecting for marker loss and screening by DNA sequencing identifies the candidate with the desired mutation (Figure 1A). Although the ability to apply both positive and negative selection for *ura4* ^+^ make it an ideal marker for this purpose [12], the cost of FOA can become limiting in a programme that targets multiple mutants to multiple loci. Similarly, the need to express human equilibrative nucleoside transporter, hENT1, to use thymidine analogues for positive selection [63] limits the appeal of this alternative approach. To generate a rapid and cheap alternative to these two options we have combined the strong positive selection of *natMX6* [58] with the *rpl42* recessive cycloheximide resistance marker system developed by Krogan and colleagues [57] in a *natMX6/rpl42* ^+^ double cassette in pFA6arpl42natMX6 (Figure 1B). Resistance to nourseothricin selects for integration of this cassette in an *rpl42.sP56Q* host strain. Subsequent replacement of the cassette by an overlapping sequence is selected for by placing transformants onto plates containing cycloheximide (Figure 1C).

### Overnight MSL-N Incubation Enhances Transformation Efficiency

Our ability to manipulate native loci has been confounded by varying efficiencies of targeted integration. For some loci, such as *fin1* ^+^, targeted integration was uncommon, with around a 5% chance that a transformant was the desired integration event, prompting us to seek strategies that may improve transformation and targeting efficiencies.

When using antibiotic selection for integrative transformation, transformants are incubated in non-selective conditions for 18 hours before applying selection to enable them to accumulate sufficient enzyme from the newly generated expression cassette to survive the otherwise lethal impact of the antibiotic [40]. Traditionally this “recovery phase” has been applied by spreading cells on non-selective plates before replica plating the ensuing lawn of cells onto antibiotic containing plates 18 hours later [40]. While highly effective, it is inevitable that the transfer efficiency during replica plating is less than 100%. Furthermore, if the targeting event compromises fitness, vigorous growth of the non-transformed host clones may out compete the less fit transformant clones. We therefore sought recovery conditions in which cell division would be blocked and yet the antibiotic metabolising enzymes could accumulate in all transformants before exposure of the entire mix of transformants and untransformed neighbours to selection pressure.

Cells are unable to divide in the absence of a nitrogen source [64]. We therefore asked whether we could simply substitute the overnight incubation on solid medium with incubation in a liquid minimal medium that lacked a nitrogen source. The MSL medium that was developed by Richard Egel [65], is ideal for this goal because it efficiently invokes a nitrogen starvation response. Amino acid supplements were omitted from this medium in our tests to limit provision of nitrogen from *in vivo* amino acid catabolism.

We used the integration of a marker at the *pku80^+^* and *leu1^+^* loci to assess the impact of MSL-N recovery phase of differing durations upon the transformation efficiency. Cells were grown to mid log phase (4×10^6^ cells ml^−1^) in rich YES medium before standard procedures were used to make the cells competent to receive DNA. The DNA fragments that were added to these competent cells were generated by PCR amplification of pFA6a antibiotic resistance deletion vector series templates with the same oligonucleotides being used with each template [40], [58]. For each marker tested the transformed cell mix was split into 6. One portion was immediately spread onto non-selective YES plates at 25°C, while the others were re-suspended in 1 ml of MSL-N and incubated with agitation at 25°C. The MSL-N transformation mixes were spread onto selective plates 2, 4, 8, 16 and 24 hours later. Cell counts confirmed that no cell division occurred during the 24 hours of incubation in MSL-N medium (data not shown). The lawn of cells on the YES plates that had received the transformation mix immediately were replica plated 20 hours after the initial spreading of the transformation mix. There were no major differences in the number of transformants between any of the protocols when uracil prototrophy was used as the selectable marker to detect *ura4* ^+^ integration (Table 1). In contrast, when antibiotic resistance formed the basis for the selection for the integration event between 5 to 8 fold more transformants were obtained in the samples that received a 24 hour MSL-N recovery period than when the aliquot had been replica plated aliquot (Table 1). PCR analysis revealed a similar rate of integrative transformation in either the replica plated or liquid recovery samples (data not shown).

**Table 1:** Transformation efficiencies.

	10^8^ cells transformed with 1 µg DNA for the indicated transformation				
	*ura4* ^+^ *into* *leu1^+^*	*kanMX6 into* *pku80^+^*	*natMX6 into* *pku80^+^*	*hphMX6 into* *pku80^+^*	*bleMX6 into* *pku80^+^*
Spread directly to selective media	950	0	0	0	0
Spread to YES, replica plate to selective media after 20 h	1000	150	140	120	100
Incubate in MSL for 2 h, spread to selective media	900	0	0	0	0
Incubate in MSL for 4 h, spread to selective media	950	3	2	0	1
Incubate in MSL for 8 h, spread to selective media	1000	4	5	3	3
Incubate in MSL for 16 h, spread to selective media	1100	650	700	600	650
Incubate in MSL for 20 h, spread to selective media	1100	850	800	750	800

The table shows the number of transformants obtained when 1 µg of the indicated DNA fragments was transformed into identical numbers of competent cells of the indicated strains. Each transformation mix was split into seven equal aliquots that were treated as indicated in the column on the left.

### Enhanced Efficiency of Integrative Targeting in *pku70.Δ* or *pku80.Δ* Backgrounds

Our attempts to target integration at a range of loci concur with the anecdotal experiences of the *S. pombe* community that the efficiency of targeting different loci varies widely. In many fungi removal of the Ku70 and Ku80 end recognition proteins blocks non-homologous end joining DNA repair pathway [49] to greatly enhances the frequency of gene targeting [50], [51], [52], [53], [54], [55], [56]. We therefore asked whether deletion of either molecule might enhance the 5% efficiency of integration at the *S. pombe fin1* ^+^ locus.

**Figure 2:**
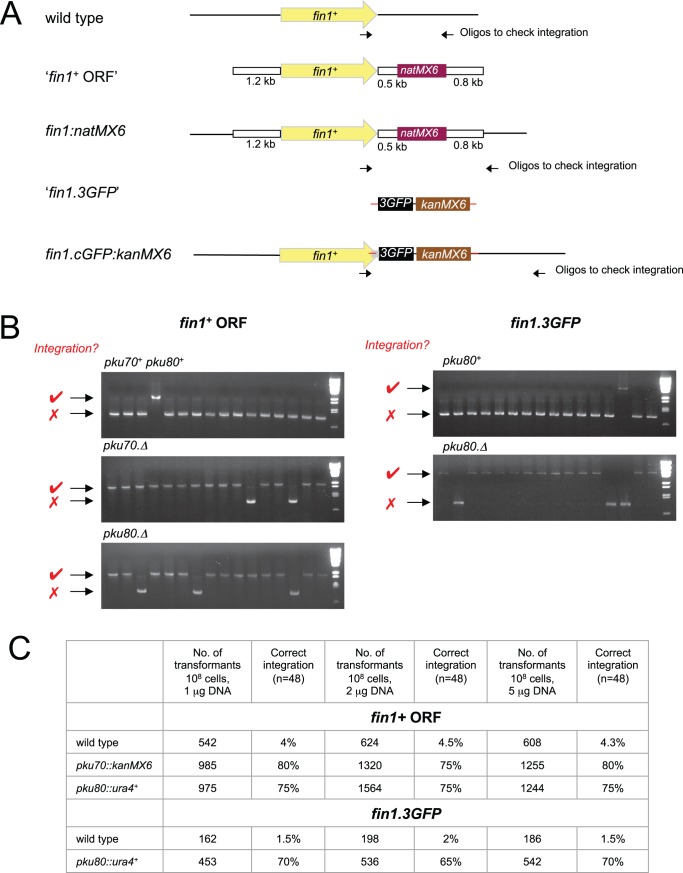
Inclusion of *pku70.Δ* and *pku80.Δ* in host strain radically enhances targeting at the *fin1* ^+^ locus. A) Cartoons depicting the structure of the DNA fragments used to direct the integration of a *natMX6* cassette 3′ to the Fin1 coding sequences at the *fin1* ^+^ locus and the integration of sequences encoding three GFP molecules, a stop codon and the *kan^R^MX6* marker at the end of the *fin1* ^+^ locus. B) PCR amplification reactions with the oligonucleotides indicated by arrows in panel A to monitor the structure of the genomic regions at the *fin1* ^+^ locus. For the “*fin1 ORF*” transformation amplification gives an 850 bp fragment (red cross next to each panel), whereas with successful integration generates an 2050 bp fragment (red tick next to each panel). For the “*fin1.3GFP*” transformation amplification with the same primers used to screen “ *fin1 ORF”* transformants generated an 850 bp fragment in the recipient host (red cross next to each panel) and an 4650 bp fragment in the correct transformant (red tick next to each panel). C) A table showing the frequency of correct integration events in the indicated strains with the indicated concentrations of each DNA fragment as determined by PCR analysis of 48 candidate transformants in each case.

Two types of DNA fragment were used for transformation: a large fragment excised from a plasmid in which the *natMX6* marker was flanked by extensive regions of homology (1.2 kb 5′ and 0.8 kb 3′) to the *fin1* ^+^ locus (Figure 2A, upper “*fin1* ^+^ ORF”) and a short fragment with 80 bp regions of homology either side of the stop codon that generated a *fin1^+^.3GFP* fusion sequence by standard PCR amplification [40] from the pSM1023 template [66], [67] (Figure 2A, lower “*fin1^+^.3GFP”*). For the “*fin1* ^+^ ORF” DNA fragment, a single sample of donor DNA was split into four. One quarter was transformed into a *pku70::kanMX6* strain, another into an otherwise isogenic *pku70* ^+^ strain and the remaining two aliquots into *pku80::ura4^+^* and isogenic *pku80* ^+^ hosts. As the selectable marker for the “*fin1^+^.3GFP”* fragment was the same geneticin/G418 resistance marker that had been used to delete *pku70* ^+^ with *kanMX6*, this *fin1^+^.3GFP* fragment was only transformed into *pku80::ura4^+^* and isogenic hosts. Diagnostic PCR analysis of transformants from each comparison revealed that the efficiency of gene targeting was elevated to between 75 and 80% (at least 16 fold increase) by the removal of either Pku70 or Pku80 (Figure 2B, C). Such a marked improvement in transformation efficiency upon removal of these end recognition factors prompted us to generate strains in which *pku70* ^+^ and *pku80* ^+^ have been replaced with the *kanMX6*, *hphMX6 and natMX6* cassettes. These strains have been deposited in the Yeast Genome Resource Centre Japan (http://yeast.lab.nig.ac.jp/nig/index_en.html, for YGRC strain numbers see Table 2).

**Table 2:** Strains used in this study.

Lab Strain number	Genotype	YGRC strain number	Source
IH5974	972 *h^−^*		Lab stock
IH1308	*ura4.D18 h^−^*		Lab stock
IH8794	*rpl42.sP56Q leu1.32 ura4.D18 h^+^*		Roguev et al. 2007
IH5221	*pku70::his3 leu1.32 his3.d1 ade6.M216 ura4.d18 h^−^*	FY23684	Lab stock
IH6067	*pku70::kanMX6 leu1.32 ura4.D18 his2 h^+^*	FY23686	Manolis et al. 2001
IH12994	*pku70::natMX6 ura4.D18*	FY23687	This study
IH12959	*pku70::hphMX6^+^ leu1.32 ura4.D18 his2 h^+^*	FY23685	This study
IH6114	*pku80::ura4^+^ leu1.32 ura4.D18 his2 h^+^*	FY23691	Manolis et al. 2001
IH13006	*pku80::kanMX6*	FY23689	This study
IH12958	*pku80::natMX6^+^ leu1.32 ura4.D18*	FY23690	This study
IH12960	*pku80::hphMX6 ura4.D18 his2 h^+^*	FY23688	This study
IH5869	*hph171k h^−^*	FY23692	This study
IH6365	*leu1::nmt41fin1.KD-pkn:ura4+ ura4.D18*		This study
IH6366	*leu1::nmt81fin1.KD-pkn:ura4+ ura4.D18*		This study
IH6364	*hph171k::nmt41fin1.KD-pkn:natMX6 ura4.D18*		This study
IH6409	*hph171k::nmt81fin1.KD-pkn:natMX6 ura4.D18*		This study

### Integration Vectors

**Figure 3:**
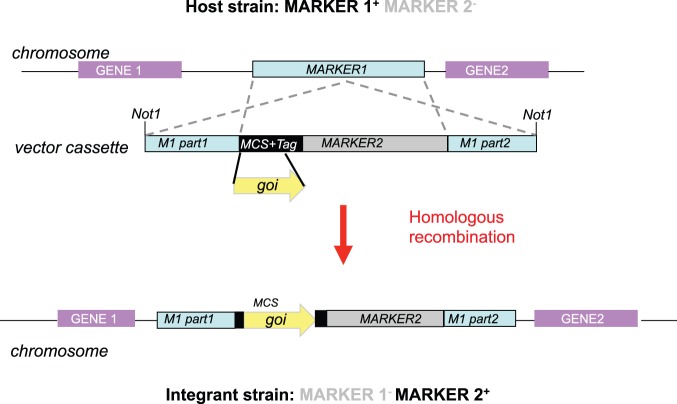
A cartoon indicating the approach used by all three integration vector systems.

Although the expression of molecules from multi-copy vectors can be highly informative, the highly variable stoichiometry of protein levels between neighbouring cells can make it difficult to derive concrete conclusions from a particular manipulation. In contrast, direct comparisons can be made between the consequences of expressing different mutant alleles when integrated in the same vector context into the same genomic location. We have therefore developed three different vector series that each direct integration of an expression cassette into distinct, defined locations in the fission yeast genome. The same principle is employed in each case; the correct integration event is identified through the simultaneous gain of one marker and the disruption, and therefore loss, of another (Figure 3). For two systems the markers are classic amino acid auxotrophies, while the third exploits dominant antibiotic markers. Each system exploits the *nmt1* based thiamine repressible promoter series: nmt1, nmt1* and nmt1** derived from the plasmids pREP1, pREP41 and pREP81 respectively [14], [15], [68]. The “A” plasmid in each series has no insert but can receive the entire promoter – gene – terminator expression cassette from existing pREP plasmids as a Pst1-Sac1 fragment. It is also the easiest recipient vector for integration of any sequence of choice.

### Auxotrophic pINTL and pINTK Integration Vectors

**Figure 4:**
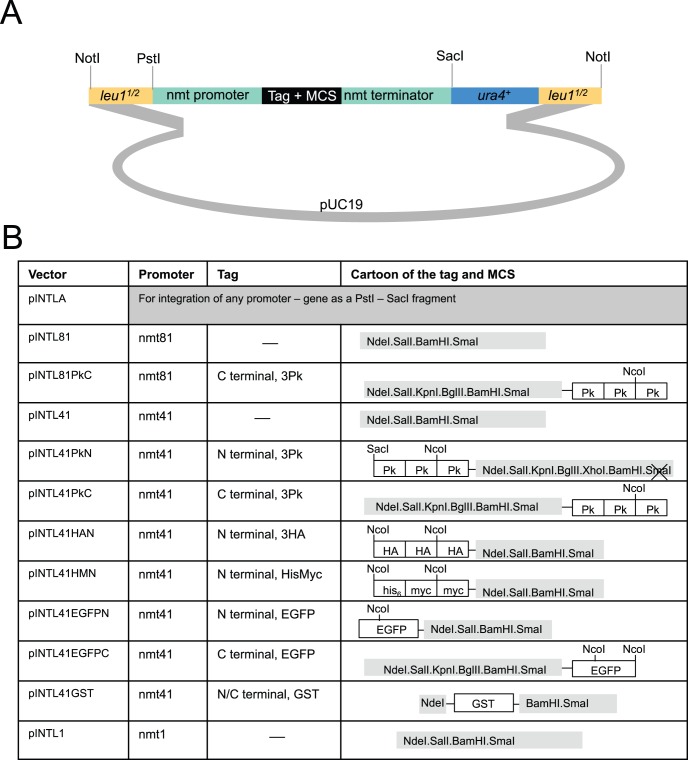
The pINTL series of vectors for the expression of a gene of interest from the *leu1* locus. Cartoons depicting the structure of the indicated pINTL vectors.

**Figure 5:**
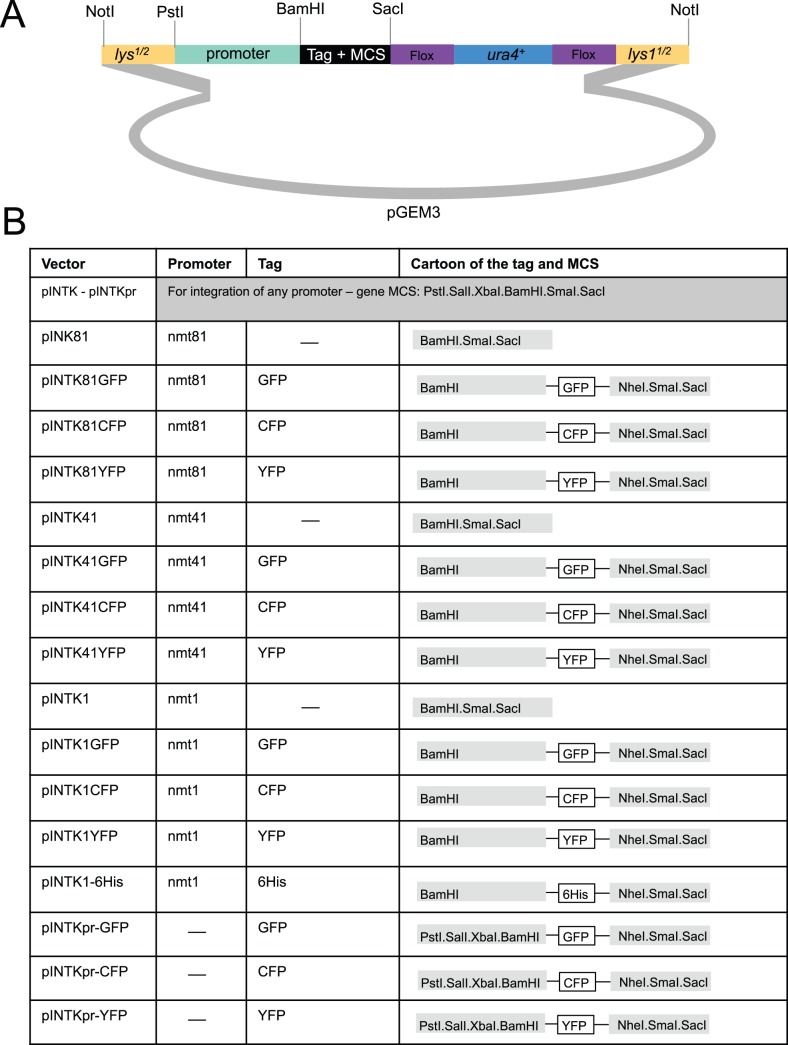
The pINTK series of vectors for the expression of a gene of interest from the *lys1* locus. Cartoons depicting the structure of the indicated pINTK vectors.

In the INTL vectors the *leu1* ^+^ gene has been disrupted by a cloning module (expression or expression + tag) and *ura4* ^+^ (Figure 4). The entire cassette is flanked by *Not*1 restriction sites. Once the desired sequences have been inserted, the *Not*1 fragment is excised and transformed into an *ura4.d18* host. In correct transformants the disruption of *leu1* ^+^ by the vector sequences flips auxotrophy from leu+ ura− to leu− ura+. INTK vectors direct the disruption of *lys1* ^+^ with a similar *ura4* ^+^ expression/tagging module to switch auxotrophy from lys+ ura− to lys− ura+ (Figure 5). The use of *ura4* ^+^ as a positive selection in both the INTL and INTK systems excluding subsequent use of *ura4^+^* based multi-copy vectors in either case. Consequently the *ura4* ^+^ sequences within the INTK cassette have been flanked with *loxP* sites to facilitate marker excision upon expression of Cre recombinase (Figure 5).

### Nourseothricin Resistance Based pINTH Integration Vectors

**Figure 6:**
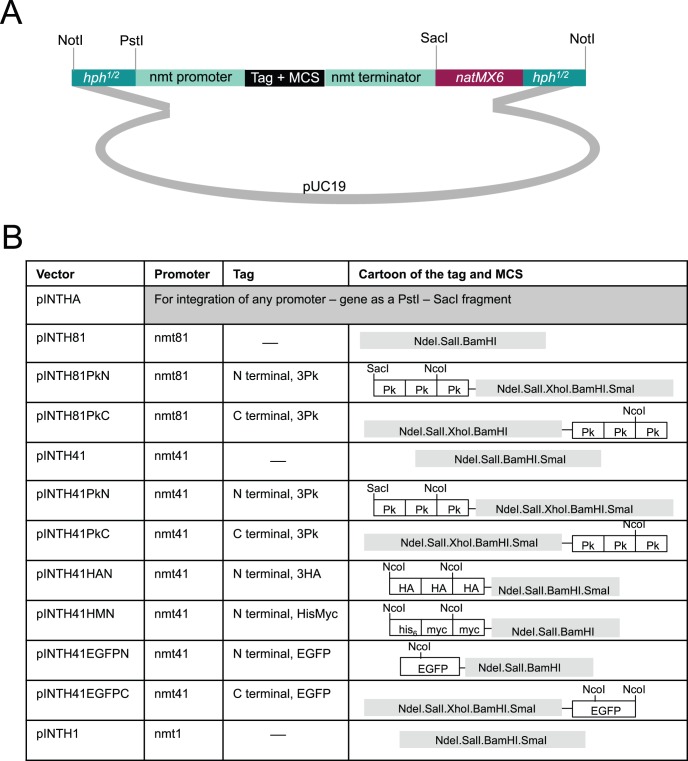
The pINTH series of vectors for the expression of a gene of interest from the *hph.171k* locus on chromosome III. Cartoons depicting the structure of the indicated pINTH vectors.

Modification of the amino acid requirements and amino acid content of the medium can significantly change flux through the TOR signalling pathway to impact upon diverse aspects of cell physiology [28]. We therefore generated an integrative vector system that can be used in prototrophs because it relies upon switching resistance to antibiotics rather than amino acid requirements. To achieve this goal we needed to select a site at which to integrate the recipient antibiotic marker that would later act as a target site for vector integration. As the smallest chromosome, chromosome III, harbours the smallest proportion of the genome of the three chromosomes, inserting a marker on this chromosome would lend itself to easier manipulation in subsequent crosses to introduce an expression cassette into a particular background. We therefore scanned chromosome III using the dataset of Wihelm *et al.* to find regions with low or no transcriptional activity [69]. Because no transcription was detected around position 171385 we integrated the Hygromycin B resistance cassette, *hphMX6*, at this site to generate an integration target locus that we refer to as the “*hph.171k*” locus (available from Yeast Genome Resource Centre Japan (http://yeast.lab.nig.ac.jp/nig/index_en.html). YGRC strain number listed in Table 2). The growth rate and fitness of *hph.171k* cells was indistinguishable from wild type at all temperatures tested (20°C, 25°C, 30°C, 32°C, 36°C) in both rich YES and minimal EMM2 medium. We then generated the pINTH series of vectors shown in Figure 6B that can be used to target any integration to any *hphMX6* sequence in the genome (Figure 6B). In our case we use it to target *hph.171k*.

**Figure 7:**
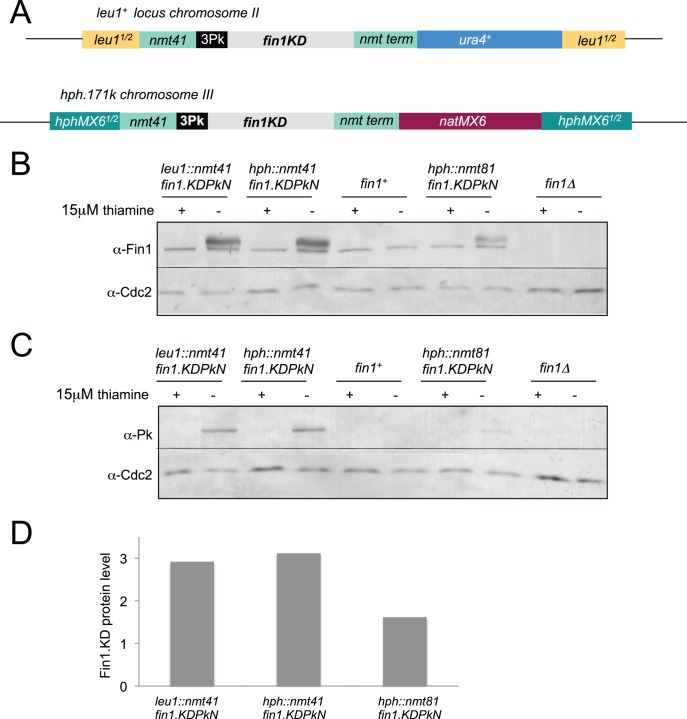
Integration at either the *hph.171k* or *leu1* loci gave identical levels of protein expression. A) Cartoons showing the structure of the two *nmt41* integrated cassettes from which catalytically inactive Fin1.KD fusion proteins (three “Pk” SV5 epitopes fused, in frame, to their amino termini) are expressed upon removal of thiamine. B) Cells were grown to early log phase in EMM2+15 µM thiamine at 25°C before being washed three times in thiamine free EMM2 medium and re-suspended in EMM2 at a density of 1.8×10^5^. Protein extracts were prepared from the mid-log phase cultures and processed for Western Blots after a further 15 hours culture at 25°C. Blots were cut in two; high molecular weight regions were probed with Fin1 antibodies while the loading control, While Cdc2 was detected on the lower molecular weight portion of the same blot. C) The same samples as shown in B probed with Cdc2 and mAb336 antibodies that recognised the Pk tags on the Fin1.KD3Pk fusion protein. D) A plot of the intensity ratios between the Fin1 and Cdc2 bands in each lane of the blots in B setting the ratio seen in wild type cells as 1 and that detected in *fin1.Δ* control as 0.

To assess the level of expression obtained following integration of the pINTH expression cassettes at *hph.171k* we cloned a *fin1* allele that encodes a catalytically inactive kinase, *fin1.KD* [62], into the pINTH41PkN vector. The *Not*1 restriction enzyme digested fragment was transformed into a *hph.171k* host (Figure 7A). 135 of the 141 transformants obtained were hygromycin resistance negative and nourseothricin resistance positive (i.e. a targeting efficiency of 96% in this *hph.171k pku70^+^ pku80^+^* host). After backcrossing, protein samples were prepared from mid-log phase cultures 15 hours after expression from the *nmt41* promoter was de-repressed by the removal of thiamine and processed for western blotting. To compare the expression level of the pINTH vectors with that obtained with the pINTL series vectors, the same *fin1.KD* insert had been cloned into the pINTL41PkN vector before integration into the genome, backcrossing and the production of protein extracts from mid-log phase cultures 15 hours after thiamine removal. The levels of Fin1.KD protein attained following induction of expression from either the *leu1* targeted pINTL41PkN or the *hph.171k* targeted pINTH41PkN construct (Figure 7B, C) were indistinguishable from one another (3 fold higher than that of the native Fin1 kinase (Figure 7B)). As expected, the expression levels from the *nmt81* based cassette was lower than from the *nmt41* cassettes. We note that the 2 fold differential in protein levels between the two strength promoters (Figure 7D) is less than the 10 fold difference reported for the production of RNA levels from the *nmt81* and *nmt41* promoters on multi-copy vectors [70], however, Fin1 is subject to proteolytic control [62] making it impossible to draw solid conclusions about transcription rates when integrated into the *leu1* ^+^ locus.

### pREPN and pREPK Vectors

**Figure 8:**
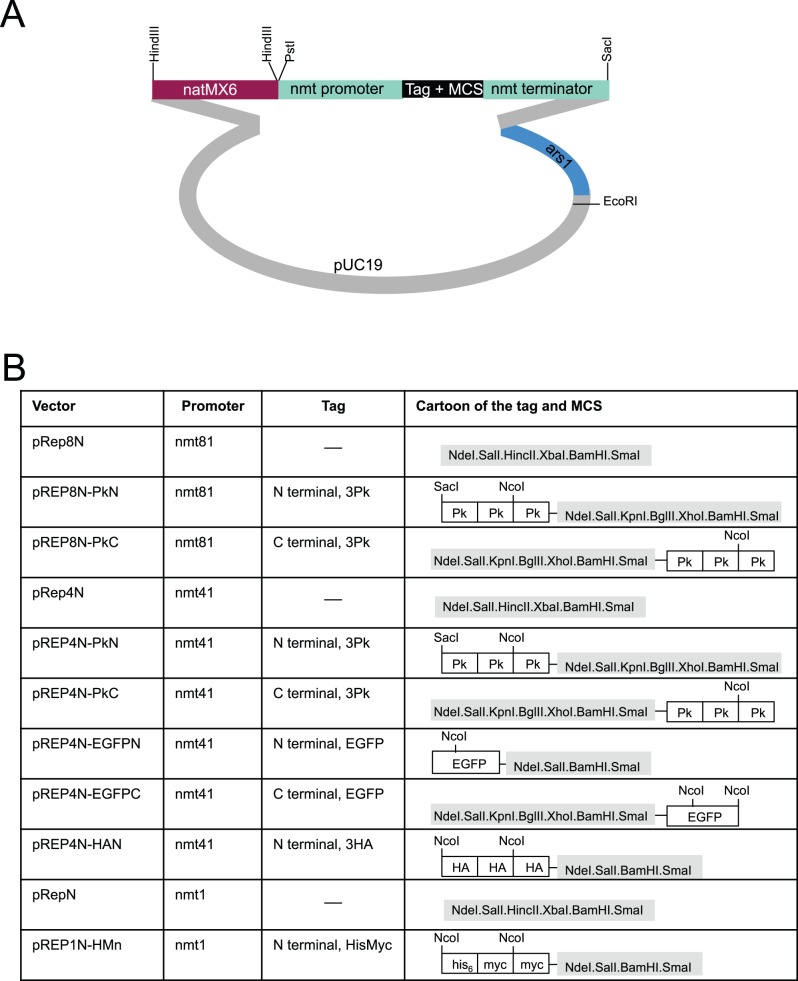
The pREPN series of vectors for the expression of a gene of interest from an ectopic plasmid. Cartoons depicting the structure of the indicated pREPN vectors.

**Figure 9:**
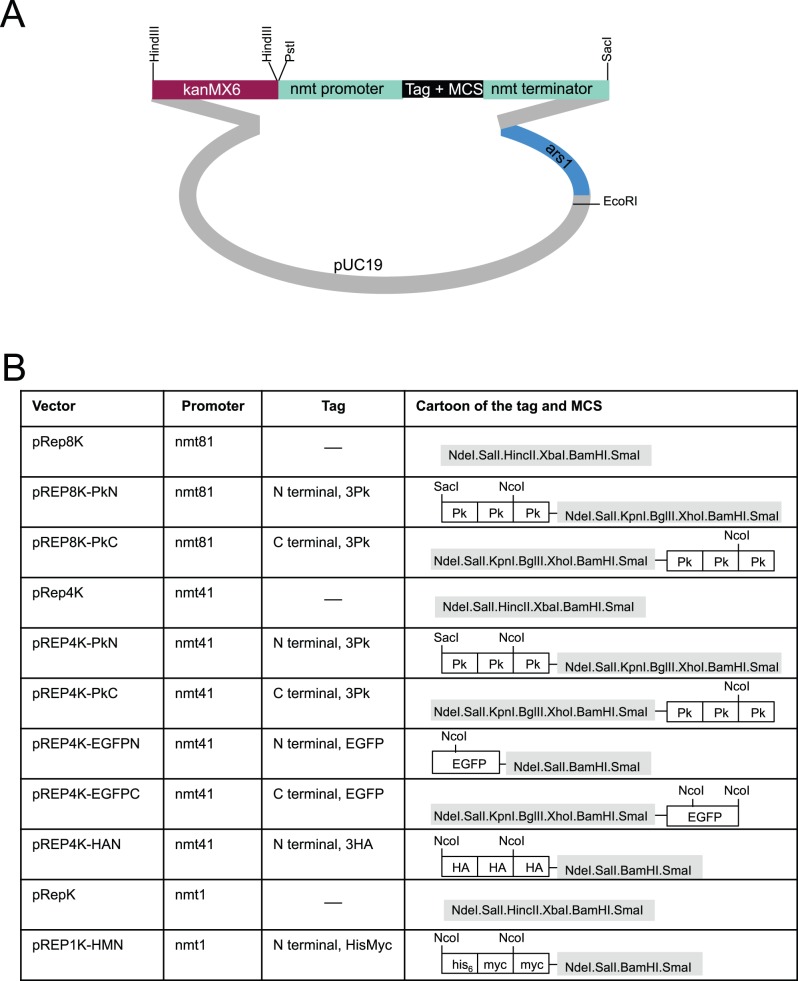
The pREPK series of vectors for the expression of a gene of interest from an ectopic plasmid. Cartoons depicting the structure of the indicated pREPK vectors.

Multi-copy plasmids that can be selected for in prototrophs to drive the expression of tagged molecules remain popular. To generate a series of vectors we used *in vivo* gap repair in fission yeast [24], [71] to switch the markers in pREP81 and pREP41/42 based vector backbones [68]. We have previously reported the construction of these donor vectors [16]. Of the four antibiotic resistance markers in use in *S. pombe*, only nourseothricin/ClonNat and Kanamycin/G418 resistance can be used in the minimal medium in which the *nmt1* based promoters of the pREP series vectors can be de-repressed, making these the only markers that would be of utility for a pREP based series of vectors. The *LEU2* ^+^ marker of pREP81 and pREP41 derived plasmids was removed by restriction digestion and the linear vector sequences were co-transformed with a DNA fragment in which the *natMX6* or the *kanMX6* cassette had been amplified with primers that had 80 bp of homology with either end of the opened vector sequences. Plasmids were re-isolated from two nat + or kan + transformants and the new vector sequenced. As reported previously for this approach [24], [71], recombination had faithfully created the desired vectors in each case (Figure 8, 9). The transformation efficiency and stability of the pREP1N plasmid was indistinguishable from that of pREP1 (data not shown).

## Discussion

We describe a number of the tools that we have developed to assist our efforts that exploit the molecular genetics of *S. pombe* to understand the signal transduction pathways that control cell division. The tools and methods presented in this paper make a significant contribution to resolving the problem of locus-dependent gene targetting efficiency. Manipulation of all loci has now become routine with the enhancements of transformation efficiency after switching the recovery incubation to an overnight incubation in un-supplemented MSL and the removal of the NHEJ response by deleting either *pku70* ^+^ or *pku80* ^+^ (the choice of which deletion to use depends upon the genomic location of the gene of interest to be targeted). We have generated *pkuX0::kanMX6*, *pkuX0::hygMX6*, *pkuX0::natMX6* strains for greatest flexibility in designing a particular knockout strategy (available from the Yeast Genome Resource Centre Japan (http://yeast.lab.nig.ac.jp/nig/index_en.html)). We note that *ura4* ^+^ and *LEU2* ^+^ deleted alleles have been generated in other studies [72], [73].

While removal of the NHEJ pathway radically enhances the frequency of gene targeting in our work on cell cycle control, our experience with genes in the TOR signalling pathway has been different as targeting can be less efficient in *pku70.Δ* and *pku80.Δ* strains than in wild type strains (data not shown). Why this should be is unclear, however we suggest that *pku70* ^+^ and *pku80* ^+^ deletions be used as host strains for manipulations of genes in processes other than TOR signalling, but reverting to targeting in a wild type prototrophic strain should targeting efficiencies prove to be poor.

We found that the efficiency of integration was radically enhanced by altering the nature of the recovery period that is used to enable the expression of antibiotic resistance markers before they are challenged with selective conditions. Switching a from recovery phase on solid medium to a liquid recovery phase in nitrogen free medium increased transformation efficiencies 5–8 fold with antibiotic resistance cassettes. Although the greatest enhancement of transformation efficiencies arose at the 24 hour time point, an overnight incubation is normally sufficient and avoids delays in strain construction. While the MSL-N recovery period was a great benefit when the integrated expression cassette directed the expression of antibiotic resistance markers to counteract the otherwise lethal impact of antibiotics, it had only a modest impact when the selection relied upon *ura4* ^+^ complementation of *ura4.d18* in media lacking uracil. We assume that this is due to the inherent differences in the nature of the selection pressure in the two cases. Expression of antibiotic resistance molecules is required to prevent an apparently immediate death from a lethal assault by the antibiotic, whereas the ornithine decarboxylase is required to permit the generation of uracil. Cells will simply remain in a stationary phase until ornithine decarboxylase levels reach the critical threshold to allow them to resume growth and division.

The generation of three series of integration vectors that allow the expression of wild type, mutant, tagged or un-tagged native molecules from three different loci greatly facilitates the analysis of the impact of mutations on individual molecules or compound interactions of mutant molecules in a protein complex. The pINTXX.A vectors can accept the entire promoter–gene–terminator cassette as a *Pst1-Sac1* restriction fragment from any existing pREP based vector [74]. Furthermore, the cloning approaches can be adapted to express full length non-coding RNAs from these sites of integration [75]. While we describe the full vector series here, we have used some members of each series in a number of studies that validate the application of these vectors in molecular cell biology in fission yeast [62], [74], [76], [77], [78], [79], [80], [81], [82], [83], [84].

## Supporting Information

Figure S1 **DNA sequences of the pINTL series.**
(DOCX)

Figure S2 **DNA sequence of pINTK.**
(DOCX)

Figure S3 **DNA sequences of the pINTH series.**
(DOCX)
