# Supplementary material for: Extending the Schizosaccharomyces pombe Molecular Genetic Toolbox
Source: PLoS One. 2014 May 21;9(5):e97683. doi: 10.1371/journal.pone.0097683 (PMC4029729; doi:10.1371/journal.pone.0097683)
Supplement: Figure S1 — DNA sequences of the pINTL series. (DOCX) [file pone.0097683.s001.docx]

**pINTLA**

atcagggttattgtctcatgagcggatacatatttgaatgtatttagaaaaataaacaaataggggttccgcgcacatttccccgaaaagtgccacctgacgtctaaggcggccgcacattataatgcatattcgtactgtctattaaacatttttttgacattaaaaatttcgtttactaacgtagaaagcgctgataattgaggctactattttaatatctagaaacaaagattcattaattaattaagtaagctccaggatacttgtatatttcgttaaaaaagcgcaatttcaacaattcctatgaacatccatgatttcatcacaaaatcaaacatatcaggatttaacgtgaattatttgctttcttgtgattttcatctgatttctggtcatttacgttactgtaaacaagctttttaaaaaaaggattttttacgcaatattatcgatatcccaatctgtagtataaaaagtgcaacagtgttgagtttcccgaaaccaggcatttttgcggtagggtctgaaactttcacgaaaccatttcacaacaaaacttcttcaacaacatgtgtgcaaagaaaatcgtcgtcttaccaggagaccatattggccctgaaattgttgcttctgccttggaggttttgaaagtcgttgagaagaagcgacctgagttaaaactcgagtttgaagaacacaagattggaggtgcctctattgatgcctatggaacccctttgactgacgagactgtgaaggcttgtttggaagctgacggtgttcttttgggtgccgttggtggtcctgaatggaccaaccccaattgtcgtcctgagcaaggtttattgaagcttcgtaagagtatgggtgtttgggccaaccttcgaccttgcaactttgccagcaagtctttagtcaagtacagccctttgaagcctgaaatcgttgaaggtgtcgatttttgtgttgtacgagaacttactggaggttgttactttggtgagcgcactgaggacaacggatcgggttatgctatggacacttggccttacagtttggaagaagtttctcgtattgctcgtttggctgcttggttagctgaaacttccaaccctcctgctcccgtcacattactcgacaaagctaatgtctgcagtgcaattgagctcgatctacaaatcccactggctatatgtatgcatttgtgttaaaaaagtttgtatagattatttaatctactcagcattctttctctaaataggaatttgttacttaatggagaaaaaaatgtttcgatttacctagtgtatttgtttgtatactcacgtttaatttcaaacatccattctatcttgtgtaatttttggcatggtgaaaaagataatcagccttataatctttacaaaagtaagaaattctgtaaataagccttaatgcccttgctttaaattaaaatggttctttttcatgataatgtttgcactttgtgaatatattttagatagttctgtgaggtataattaagatgttttagagacttatacaattttgtctttataaattcttaattgattttaccatcccagtttaactatgcttcgtcggcatctctgcacatgtcgtgttttcttaccgtattgtcctaccaagaacctcttttttgcttggatcgaaattaaaggtttaaaagcaaagttatggatgctagagtatttcaaagctattcagctagagctgaggggatgaaaaatcccattgccaaggaattgttggctttgatggaagaaaagcaaagcaacttgtcagtcgcggtcgatttgacgaagaaatccgaaatcttagaattggtagataaaattggaccctatgtctgtgttatcaagacacatattgacgttgtcgaggatttcgaccaggatatggtagaaaaactggtggccttaggtaaaaagcatcgttttcttatctttgaggatcgcaaattcgcagacattggaaataccgtcaagctacaatatgcatctggtgtgtacaaaattgcttcttgggctcatatcacaaattgccatacagtgccaggcgagggtattatacaaggcctcaaagaagttggtttacctttgggacgtggtctcttgcttttggctgaaatgtcttccaaaggctctttggctactggttcctacacagagaaaaccttagaatggtttgagaagcataccgatttttgctttggctttatagctggtcgtcgatttcctaaccttcaaagcgactacataactatgtcccctggtatcggcttggatgttaaaggagacgggctgggacagcaatatcgtactcctgaagaagtgattgtaaactgcggtagcgatatcatcattgttggtcgtggagtctatggagctggtcgtaatcctgttgtcgaagccaagagatatagagaagctggttggaaggcatatcagcaaagactttctcagcattaaaaaaagactaatgtaaaatttttttggttggttattgaaaaagtcgatgccttgtttgcgtttgttttcctaggcgttttatgtcagaaggcatttagaattagtatacaagtactctttggtaaaattttatgtagcgactaaaatattaactattatagataaacaccttgggaataaaaagtaatttgctatagtaatttattaaacatgctcctacaacattaccacaatcttttctcttggattgacattgaataagaaaagagtgaatttttttagacttgtaatgataactatgtacaaagccaatgaaagatgtatgtagatgaatgtaaaataccatgtagacaaacaagataaaacttggttataaacattggtgttggaacagaataaattagatgtcaaaaagtttcgtcaatatcacgatttgatttgacctaatttttattgattaattgtgtcgcatatttattatcgttaaaaatgcaattgacaaaagcgtaaattttaaggctctaatgttttttcttttggtatgttatagtcaagcaaagatgtattaagttgcaaaagcatggaattgatttacgacatccgttcaactttatgaccgagttgtcgctgtgtacttactctttttttactatgaaaagtttcaaacataatatcattaattatttctttttaaacgaagtaacctgtaacataaataataggggaaaagaagaaaaaaaccataaaaattcgagaacgtaacaattattattgtaacgatgacaacccgattctttgagagagccagagacatgacttttgcattaccgtaattggtttctacttatgaattgtaaacttgtgtagggaatttcatccacagctatcgcgggctgctctctgcatcagtgttagcacattttatattaatgaagattttgctatgcaaaatacgtacatttctggcgcatatatttttatcaaacgaacatgactatcctttccactttgatggttcgcaagtattttgatttgtattaaatgcacatctaacaagccatttcgcccaaaatcaccaactggcacatgtacccaaccagttggttcctcgacttgaactgttgtaacaatttccaaatcttgaaaccctgtccgcggccgcccagctgcattaatgaatcggccaacgcgcggggagaggcggtttgcgtattgggcgctcttccgcttcctcgctcactgactcgctgcgctcggtcgttcggctgcggcgagcggtatcagctcactcaaaggcggtaatacggttatccacagaatcaggggataacgcaggaaagaacatgtgagcaaaaggccagcaaaaggccaggaaccgtaaaaaggccgcgttgctggcgtttttccataggctccgcccccctgacgagcatcacaaaaatcgacgctcaagtcagaggtggcgaaacccgacaggactataaagataccaggcgtttccccctggaagctccctcgtgcgctctcctgttccgaccctgccgcttaccggatacctgtccgcctttctcccttcgggaagcgtggcgctttctcatagctcacgctgtaggtatctcagttcggtgtaggtcgttcgctccaagctgggctgtgtgcacgaaccccccgttcagcccgaccgctgcgccttatccggtaactatcgtcttgagtccaacccggtaagacacgacttatcgccactggcagcagccactggtaacaggattagcagagcgaggtatgtaggcggtgctacagagttcttgaagtggtggcctaactacggctacactagaagaacagtatttggtatctgcgctctgctgaagccagttaccttcggaaaaagagttggtagctcttgatccggcaaacaaaccaccgctggtagcggtggtttttttgtttgcaagcagcagattacgcgcagaaaaaaaggatctcaagaagatcctttgatcttttctacggggtctgacgctcagtggaacgaaaactcacgttaagggattttggtcatgagattatcaaaaaggatcttcacctagatccttttaaattaaaaatgaagttttaaatcaatctaaagtatatatgagtaaacttggtctgacagttaccaatgcttaatcagtgaggcacctatctcagcgatctgtctatttcgttcatccatagttgcctgactccccgtcgtgtagataactacgatacgggagggcttaccatctggccccagtgctgcaatgataccgcgagacccacgctcaccggctccagatttatcagcaataaaccagccagccggaagggccgagcgcagaagtggtcctgcaactttatccgcctccatccagtctattaattgttgccgggaagctagagtaagtagttcgccagttaatagtttgcgcaacgttgttgccattgctacaggcatcgtggtgtcacgctcgtcgtttggtatggcttcattcagctccggttcccaacgatcaaggcgagttacatgatcccccatgttgtgcaaaaaagcggttagctccttcggtcctccgatcgttgtcagaagtaagttggccgcagtgttatcactcatggttatggcagcactgcataattctcttactgtcatgccatccgtaagatgcttttctgtgactggtgagtactcaaccaagtcattctgagaatagtgtatgcggcgaccgagttgctcttgcccggcgtcaatacgggataataccgcgccacatagcagaactttaaaagtgctcatcattggaaaacgttcttcggggcgaaaactctcaaggatcttaccgctgttgagatccagttcgatgtaacccactcgtgcacccaactgatcttcagcatcttttactttcaccagcgtttctgggtgagcaaaaacaggaaggcaaaatgccgcaaaaaagggaataagggcgacacggaaatgttgaatactcatactcttcctttttcaatattattgaagcattt

Leu1 frag

Ura4 frag

beta-lactamase (AmpR)

**pINTL81**

atcagggttattgtctcatgagcggatacatatttgaatgtatttagaaaaataaacaaataggggttccgcgcacatttccccgaaaagtgccacctgacgtctaaggcggccgcacattataatgcatattcgtactgtctattaaacatttttttgacattaaaaatttcgtttactaacgtagaaagcgctgataattgaggctactattttaatatctagaaacaaagattcattaattaattaagtaagctccaggatacttgtatatttcgttaaaaaagcgcaatttcaacaattcctatgaacatccatgatttcatcacaaaatcaaacatatcaggatttaacgtgaattatttgctttcttgtgattttcatctgatttctggtcatttacgttactgtaaacaagctttttaaaaaaaggattttttacgcaatattatcgatatcccaatctgtagtataaaaagtgcaacagtgttgagtttcccgaaaccaggcatttttgcggtagggtctgaaactttcacgaaaccatttcacaacaaaacttcttcaacaacatgtgtgcaaagaaaatcgtcgtcttaccaggagaccatattggccctgaaattgttgcttctgccttggaggttttgaaagtcgttgagaagaagcgacctgagttaaaactcgagtttgaagaacacaagattggaggtgcctctattgatgcctatggaacccctttgactgacgagactgtgaaggcttgtttggaagctgacggtgttcttttgggtgccgttggtggtcctgaatggaccaaccccaattgtcgtcctgagcaaggtttattgaagcttcgtaagagtatgggtgtttgggccaaccttcgaccttgcaactttgccagcaagtctttagtcaagtacagccctttgaagcctgaaatcgttgaaggtgtcgatttttgtgttgtacgagaacttactggaggttgttactttggtgagcgcactgaggacaacggatcgggttatgctatggacacttggccttacagtttggaagaagtttctcgtattgctcgtttggctgcttggttagctgaaacttccaaccctcctgctcccgtcacattactcgacaaagctaatgtctgcaggtcgatcgactctagaggatcagaaaattatcgccataaaagacagaataagtcatcagcggttgtttcatttcctatattttttttttatttttttattttttaataagggaaaatttaacgtctaaggatacagaagattgttagcacattaaagtaataaaggcttaagtagtaagtgccttagcatgttattgtatttcaaaggacataatctaaaataataacaatatcatttctcacaagttattcaattttcttttttttttctaataatatcaagaatgtattatttgtttgacataagtcaactaatttatttaatatgctggattaatcttgcagacatgtaaattaacaagttttagtcaaataacgttgaagtttcaatgaactcaaataatttctctttttttttatataaccataatctgatttatattttccgcagggatcaactgaagttatgacatttggattggatcacttataaccttggtcgccaaataatacaaaaatcagcgttataaaacaaagaaggtttttgttaagaaattaatcctctttcttgataagaaagttgaaccgaaattgcagatactgatatatgaaaataatacccacaattttgggaatagcgcaagcctcaatttaaacaataggtgaggacacatgataatgacctcaatgattgttagaagaaaagagcctcattacaaaatcgaaaaatgaatggttgggtacaagtttccaaaacatggtaaagtggactttgcgtatgagacgtaaatagaaaaaaacacttgttatatgttttctagaattattgttgtctctttatggttggatgatgcaaaatagtaatttcggttagttgctgtaaaacaccacgagacaaatagatatggatatttattaaatcaggaaaaacgtaactctcggctactggatggttcagtcacccaacgattactggggagagaaaacagggcaaaagcaaagcttaaaggaatccgattgtcattcggcaatgtgcagcgaaactaaaaaccggataatggacctgttaatcgaaacattgaagatggaagaggaatcctggcatatcatcaattgaataagttgaattaattatttcaatctcattctcactttctgacttatagtcgctttgttaaatcatatgtcgactctagaggatccccgggtaaaaggaatgtctcccttgccagtactgctagggtttttctttcaaactatggaagcccattcaagctgcatattacgattttgtttttcgcttttagaaagtggtttagatgagataatagaaaaattcttgatctccgacaacgagtacttttattttttttgctaatcactttactcaatattagctcgaaatcgtagaaacgtagacgggtgcgggataccgagtggtgtagttaagaatttttataaaccacgtggcccaaaaatatgaacccaaaacgtttatacatgagtatactttaagaaggctataccccttcgtgttagatgtagttttagctacccaacccgagtctatgagcttgacttcagatgtagaaggcattaaatcgttttgaatattaattaaaaaacgatgaaaattaaatatttaaaagcaatcatacgctgaaaatttagtgctgtggctaatccttcaacatggaaatgccataaaagtgactttgacaaaaaaaaaagtatatacaggtagtaaactcatctacttcattgactttgtttacagcatgtggaaggaggaatatttattgctaaatcgtagtttaacattcaataagtaatactattgaaattcgacaagattggccgcatggatgaaaaagaggcattttgctttgggagaattagtccaaattagaactgaaaaaaaaaactttacgaggcaaaaatgtcggattgagatcgtaaaagttcgctcgtcgtcttttgctttgtgattgttttcatggatacatcttgctggatatttaaattttagtactatgtataagatattctataaatgttttatcacccaaacctgttagcgccttcttaattctattcaatctggcttttgctctgagactacttcttggactttcactacttgttagttatacggaatttgtgtaattagaagtgaaataatcctttctattagtaatgcgagctcgatctacaaatcccactggctatatgtatgcatttgtgttaaaaaagtttgtatagattatttaatctactcagcattctttctctaaataggaatttgttacttaatggagaaaaaaatgtttcgatttacctagtgtatttgtttgtatactcacgtttaatttcaaacatccattctatcttgtgtaatttttggcatggtgaaaaagataatcagccttataatctttacaaaagtaagaaattctgtaaataagccttaatgcccttgctttaaattaaaatggttctttttcatgataatgtttgcactttgtgaatatattttagatagttctgtgaggtataattaagatgttttagagacttatacaattttgtctttataaattcttaattgattttaccatcccagtttaactatgcttcgtcggcatctctgcacatgtcgtgttttcttaccgtattgtcctaccaagaacctcttttttgcttggatcgaaattaaaggtttaaaagcaaagttatggatgctagagtatttcaaagctattcagctagagctgaggggatgaaaaatcccattgccaaggaattgttggctttgatggaagaaaagcaaagcaacttgtcagtcgcggtcgatttgacgaagaaatccgaaatcttagaattggtagataaaattggaccctatgtctgtgttatcaagacacatattgacgttgtcgaggatttcgaccaggatatggtagaaaaactggtggccttaggtaaaaagcatcgttttcttatctttgaggatcgcaaattcgcagacattggaaataccgtcaagctacaatatgcatctggtgtgtacaaaattgcttcttgggctcatatcacaaattgccatacagtgccaggcgagggtattatacaaggcctcaaagaagttggtttacctttgggacgtggtctcttgcttttggctgaaatgtcttccaaaggctctttggctactggttcctacacagagaaaaccttagaatggtttgagaagcataccgatttttgctttggctttatagctggtcgtcgatttcctaaccttcaaagcgactacataactatgtcccctggtatcggcttggatgttaaaggagacgggctgggacagcaatatcgtactcctgaagaagtgattgtaaactgcggtagcgatatcatcattgttggtcgtggagtctatggagctggtcgtaatcctgttgtcgaagccaagagatatagagaagctggttggaaggcatatcagcaaagactttctcagcattaaaaaaagactaatgtaaaatttttttggttggttattgaaaaagtcgatgccttgtttgcgtttgttttcctaggcgttttatgtcagaaggcatttagaattagtatacaagtactctttggtaaaattttatgtagcgactaaaatattaactattatagataaacaccttgggaataaaaagtaatttgctatagtaatttattaaacatgctcctacaacattaccacaatcttttctcttggattgacattgaataagaaaagagtgaatttttttagacttgtaatgataactatgtacaaagccaatgaaagatgtatgtagatgaatgtaaaataccatgtagacaaacaagataaaacttggttataaacattggtgttggaacagaataaattagatgtcaaaaagtttcgtcaatatcacgatttgatttgacctaatttttattgattaattgtgtcgcatatttattatcgttaaaaatgcaattgacaaaagcgtaaattttaaggctctaatgttttttcttttggtatgttatagtcaagcaaagatgtattaagttgcaaaagcatggaattgatttacgacatccgttcaactttatgaccgagttgtcgctgtgtacttactctttttttactatgaaaagtttcaaacataatatcattaattatttctttttaaacgaagtaacctgtaacataaataataggggaaaagaagaaaaaaaccataaaaattcgagaacgtaacaattattattgtaacgatgacaacccgattctttgagagagccagagacatgacttttgcattaccgtaattggtttctacttatgaattgtaaacttgtgtagggaatttcatccacagctatcgcgggctgctctctgcatcagtgttagcacattttatattaatgaagattttgctatgcaaaatacgtacatttctggcgcatatatttttatcaaacgaacatgactatcctttccactttgatggttcgcaagtattttgatttgtattaaatgcacatctaacaagccatttcgcccaaaatcaccaactggcacatgtacccaaccagttggttcctcgacttgaactgttgtaacaatttccaaatcttgaaaccctgtccgcggccgcccagctgcattaatgaatcggccaacgcgcggggagaggcggtttgcgtattgggcgctcttccgcttcctcgctcactgactcgctgcgctcggtcgttcggctgcggcgagcggtatcagctcactcaaaggcggtaatacggttatccacagaatcaggggataacgcaggaaagaacatgtgagcaaaaggccagcaaaaggccaggaaccgtaaaaaggccgcgttgctggcgtttttccataggctccgcccccctgacgagcatcacaaaaatcgacgctcaagtcagaggtggcgaaacccgacaggactataaagataccaggcgtttccccctggaagctccctcgtgcgctctcctgttccgaccctgccgcttaccggatacctgtccgcctttctcccttcgggaagcgtggcgctttctcatagctcacgctgtaggtatctcagttcggtgtaggtcgttcgctccaagctgggctgtgtgcacgaaccccccgttcagcccgaccgctgcgccttatccggtaactatcgtcttgagtccaacccggtaagacacgacttatcgccactggcagcagccactggtaacaggattagcagagcgaggtatgtaggcggtgctacagagttcttgaagtggtggcctaactacggctacactagaagaacagtatttggtatctgcgctctgctgaagccagttaccttcggaaaaagagttggtagctcttgatccggcaaacaaaccaccgctggtagcggtggtttttttgtttgcaagcagcagattacgcgcagaaaaaaaggatctcaagaagatcctttgatcttttctacggggtctgacgctcagtggaacgaaaactcacgttaagggattttggtcatgagattatcaaaaaggatcttcacctagatccttttaaattaaaaatgaagttttaaatcaatctaaagtatatatgagtaaacttggtctgacagttaccaatgcttaatcagtgaggcacctatctcagcgatctgtctatttcgttcatccatagttgcctgactccccgtcgtgtagataactacgatacgggagggcttaccatctggccccagtgctgcaatgataccgcgagacccacgctcaccggctccagatttatcagcaataaaccagccagccggaagggccgagcgcagaagtggtcctgcaactttatccgcctccatccagtctattaattgttgccgggaagctagagtaagtagttcgccagttaatagtttgcgcaacgttgttgccattgctacaggcatcgtggtgtcacgctcgtcgtttggtatggcttcattcagctccggttcccaacgatcaaggcgagttacatgatcccccatgttgtgcaaaaaagcggttagctccttcggtcctccgatcgttgtcagaagtaagttggccgcagtgttatcactcatggttatggcagcactgcataattctcttactgtcatgccatccgtaagatgcttttctgtgactggtgagtactcaaccaagtcattctgagaatagtgtatgcggcgaccgagttgctcttgcccggcgtcaatacgggataataccgcgccacatagcagaactttaaaagtgctcatcattggaaaacgttcttcggggcgaaaactctcaaggatcttaccgctgttgagatccagttcgatgtaacccactcgtgcacccaactgatcttcagcatcttttactttcaccagcgtttctgggtgagcaaaaacaggaaggcaaaatgccgcaaaaaagggaataagggcgacacggaaatgttgaatactcatactcttcctttttcaatattattgaagcattt

Leu1frag

Nmt81 prom

MCS

nmt term

Ura4 frag

beta-lactamase (AmpR)

**pINTL81PKC**

atcagggttattgtctcatgagcggatacatatttgaatgtatttagaaaaataaacaaataggggttccgcgcacatttccccgaaaagtgccacctgacgtctaaggcggccgcacattataatgcatattcgtactgtctattaaacatttttttgacattaaaaatttcgtttactaacgtagaaagcgctgataattgaggctactattttaatatctagaaacaaagattcattaattaattaagtaagctccaggatacttgtatatttcgttaaaaaagcgcaatttcaacaattcctatgaacatccatgatttcatcacaaaatcaaacatatcaggatttaacgtgaattatttgctttcttgtgattttcatctgatttctggtcatttacgttactgtaaacaagctttttaaaaaaaggattttttacgcaatattatcgatatcccaatctgtagtataaaaagtgcaacagtgttgagtttcccgaaaccaggcatttttgcggtagggtctgaaactttcacgaaaccatttcacaacaaaacttcttcaacaacatgtgtgcaaagaaaatcgtcgtcttaccaggagaccatattggccctgaaattgttgcttctgccttggaggttttgaaagtcgttgagaagaagcgacctgagttaaaactcgagtttgaagaacacaagattggaggtgcctctattgatgcctatggaacccctttgactgacgagactgtgaaggcttgtttggaagctgacggtgttcttttgggtgccgttggtggtcctgaatggaccaaccccaattgtcgtcctgagcaaggtttattgaagcttcgtaagagtatgggtgtttgggccaaccttcgaccttgcaactttgccagcaagtctttagtcaagtacagccctttgaagcctgaaatcgttgaaggtgtcgatttttgtgttgtacgagaacttactggaggttgttactttggtgagcgcactgaggacaacggatcgggttatgctatggacacttggccttacagtttggaagaagtttctcgtattgctcgtttggctgcttggttagctgaaacttccaaccctcctgctcccgtcacattactcgacaaagctaatgtctgcaggtcgatcgactctagaggatcagaaaattatcgccataaaagacagaataagtcatcagcggttgtttcatttcctatattttttttttatttttttattttttaataagggaaaatttaacgtctaaggatacagaagattgttagcacattaaagtaataaaggcttaagtagtaagtgccttagcatgttattgtatttcaaaggacataatctaaaataataacaatatcatttctcacaagttattcaattttcttttttttttctaataatatcaagaatgtattatttgtttgacataagtcaactaatttatttaatatgctggattaatcttgcagacatgtaaattaacaagttttagtcaaataacgttgaagtttcaatgaactcaaataatttctctttttttttatataaccataatctgatttatattttccgcagggatcaactgaagttatgacatttggattggatcacttataaccttggtcgccaaataatacaaaaatcagcgttataaaacaaagaaggtttttgttaagaaattaatcctctttcttgataagaaagttgaaccgaaattgcagatactgatatatgaaaataatacccacaattttgggaatagcgcaagcctcaatttaaacaataggtgaggacacatgataatgacctcaatgattgttagaagaaaagagcctcattacaaaatcgaaaaatgaatggttgggtacaagtttccaaaacatggtaaagtggactttgcgtatgagacgtaaatagaaaaaaacacttgttatatgttttctagaattattgttgtctctttatggttggatgatgcaaaatagtaatttcggttagttgctgtaaaacaccacgagacaaatagatatggatatttattaaatcaggaaaaacgtaactctcggctactggatggttcagtcacccaacgattactggggagagaaaacagggcaaaagcaaagcttaaaggaatccgattgtcattcggcaatgtgcagcgaaactaaaaaccggataatggacctgttaatcgaaacattgaagatggaagaggaatcctggcatatcatcaattgaataagttgaattaattatttcaatctcattctcactttctgacttatagtcgctttgttaaatcatatgtcgacatggtaccagatctctcgaggatccccgggtatgggtattcctaaccctttgcttggtcttgatggtattcctaaccctttgcttggtcttgattccatgggtattcctaaccctttgcttggtcttgatcattaaggatcgggtaaaaggaatgtctcccttgccagtactgctagggtttttctttcaaactatggaagcccattcaagctgcatattacgattttgtttttcgcttttagaaagtggtttagatgagataatagaaaaattcttgatctccgacaacgagtacttttattttttttgctaatcactttactcaatattagctcgaaatcgtagaaacgtagacgggtgcgggataccgagtggtgtagttaagaatttttataaaccacgtggcccaaaaatatgaacccaaaacgtttatacatgagtatactttaagaaggctataccccttcgtgttagatgtagttttagctacccaacccgagtctatgagcttgacttcagatgtagaaggcattaaatcgttttgaatattaattaaaaaacgatgaaaattaaatatttaaaagcaatcatacgctgaaaatttagtgctgtggctaatccttcaacatggaaatgccataaaagtgactttgacaaaaaaaaaagtatatacaggtagtaaactcatctacttcattgactttgtttacagcatgtggaaggaggaatatttattgctaaatcgtagtttaacattcaataagtaatactattgaaattcgacaagattggccgcatggatgaaaaagaggcattttgctttgggagaattagtccaaattagaactgaaaaaaaaaactttacgaggcaaaaatgtcggattgagatcgtaaaagttcgctcgtcgtcttttgctttgtgattgttttcatggatacatcttgctggatatttaaattttagtactatgtataagatattctataaatgttttatcacccaaacctgttagcgccttcttaattctattcaatctggcttttgctctgagactacttcttggactttcactacttgttagttatacggaatttgtgtaattagaagtgaaataatcctttctattagtaatgcgagctcgatctacaaatcccactggctatatgtatgcatttgtgttaaaaaagtttgtatagattatttaatctactcagcattctttctctaaataggaatttgttacttaatggagaaaaaaatgtttcgatttacctagtgtatttgtttgtatactcacgtttaatttcaaacatccattctatcttgtgtaatttttggcatggtgaaaaagataatcagccttataatctttacaaaagtaagaaattctgtaaataagccttaatgcccttgctttaaattaaaatggttctttttcatgataatgtttgcactttgtgaatatattttagatagttctgtgaggtataattaagatgttttagagacttatacaattttgtctttataaattcttaattgattttaccatcccagtttaactatgcttcgtcggcatctctgcacatgtcgtgttttcttaccgtattgtcctaccaagaacctcttttttgcttggatcgaaattaaaggtttaaaagcaaagttatggatgctagagtatttcaaagctattcagctagagctgaggggatgaaaaatcccattgccaaggaattgttggctttgatggaagaaaagcaaagcaacttgtcagtcgcggtcgatttgacgaagaaatccgaaatcttagaattggtagataaaattggaccctatgtctgtgttatcaagacacatattgacgttgtcgaggatttcgaccaggatatggtagaaaaactggtggccttaggtaaaaagcatcgttttcttatctttgaggatcgcaaattcgcagacattggaaataccgtcaagctacaatatgcatctggtgtgtacaaaattgcttcttgggctcatatcacaaattgccatacagtgccaggcgagggtattatacaaggcctcaaagaagttggtttacctttgggacgtggtctcttgcttttggctgaaatgtcttccaaaggctctttggctactggttcctacacagagaaaaccttagaatggtttgagaagcataccgatttttgctttggctttatagctggtcgtcgatttcctaaccttcaaagcgactacataactatgtcccctggtatcggcttggatgttaaaggagacgggctgggacagcaatatcgtactcctgaagaagtgattgtaaactgcggtagcgatatcatcattgttggtcgtggagtctatggagctggtcgtaatcctgttgtcgaagccaagagatatagagaagctggttggaaggcatatcagcaaagactttctcagcattaaaaaaagactaatgtaaaatttttttggttggttattgaaaaagtcgatgccttgtttgcgtttgttttcctaggcgttttatgtcagaaggcatttagaattagtatacaagtactctttggtaaaattttatgtagcgactaaaatattaactattatagataaacaccttgggaataaaaagtaatttgctatagtaatttattaaacatgctcctacaacattaccacaatcttttctcttggattgacattgaataagaaaagagtgaatttttttagacttgtaatgataactatgtacaaagccaatgaaagatgtatgtagatgaatgtaaaataccatgtagacaaacaagataaaacttggttataaacattggtgttggaacagaataaattagatgtcaaaaagtttcgtcaatatcacgatttgatttgacctaatttttattgattaattgtgtcgcatatttattatcgttaaaaatgcaattgacaaaagcgtaaattttaaggctctaatgttttttcttttggtatgttatagtcaagcaaagatgtattaagttgcaaaagcatggaattgatttacgacatccgttcaactttatgaccgagttgtcgctgtgtacttactctttttttactatgaaaagtttcaaacataatatcattaattatttctttttaaacgaagtaacctgtaacataaataataggggaaaagaagaaaaaaaccataaaaattcgagaacgtaacaattattattgtaacgatgacaacccgattctttgagagagccagagacatgacttttgcattaccgtaattggtttctacttatgaattgtaaacttgtgtagggaatttcatccacagctatcgcgggctgctctctgcatcagtgttagcacattttatattaatgaagattttgctatgcaaaatacgtacatttctggcgcatatatttttatcaaacgaacatgactatcctttccactttgatggttcgcaagtattttgatttgtattaaatgcacatctaacaagccatttcgcccaaaatcaccaactggcacatgtacccaaccagttggttcctcgacttgaactgttgtaacaatttccaaatcttgaaaccctgtccgcggccgcccagctgcattaatgaatcggccaacgcgcggggagaggcggtttgcgtattgggcgctcttccgcttcctcgctcactgactcgctgcgctcggtcgttcggctgcggcgagcggtatcagctcactcaaaggcggtaatacggttatccacagaatcaggggataacgcaggaaagaacatgtgagcaaaaggccagcaaaaggccaggaaccgtaaaaaggccgcgttgctggcgtttttccataggctccgcccccctgacgagcatcacaaaaatcgacgctcaagtcagaggtggcgaaacccgacaggactataaagataccaggcgtttccccctggaagctccctcgtgcgctctcctgttccgaccctgccgcttaccggatacctgtccgcctttctcccttcgggaagcgtggcgctttctcatagctcacgctgtaggtatctcagttcggtgtaggtcgttcgctccaagctgggctgtgtgcacgaaccccccgttcagcccgaccgctgcgccttatccggtaactatcgtcttgagtccaacccggtaagacacgacttatcgccactggcagcagccactggtaacaggattagcagagcgaggtatgtaggcggtgctacagagttcttgaagtggtggcctaactacggctacactagaagaacagtatttggtatctgcgctctgctgaagccagttaccttcggaaaaagagttggtagctcttgatccggcaaacaaaccaccgctggtagcggtggtttttttgtttgcaagcagcagattacgcgcagaaaaaaaggatctcaagaagatcctttgatcttttctacggggtctgacgctcagtggaacgaaaactcacgttaagggattttggtcatgagattatcaaaaaggatcttcacctagatccttttaaattaaaaatgaagttttaaatcaatctaaagtatatatgagtaaacttggtctgacagttaccaatgcttaatcagtgaggcacctatctcagcgatctgtctatttcgttcatccatagttgcctgactccccgtcgtgtagataactacgatacgggagggcttaccatctggccccagtgctgcaatgataccgcgagacccacgctcaccggctccagatttatcagcaataaaccagccagccggaagggccgagcgcagaagtggtcctgcaactttatccgcctccatccagtctattaattgttgccgggaagctagagtaagtagttcgccagttaatagtttgcgcaacgttgttgccattgctacaggcatcgtggtgtcacgctcgtcgtttggtatggcttcattcagctccggttcccaacgatcaaggcgagttacatgatcccccatgttgtgcaaaaaagcggttagctccttcggtcctccgatcgttgtcagaagtaagttggccgcagtgttatcactcatggttatggcagcactgcataattctcttactgtcatgccatccgtaagatgcttttctgtgactggtgagtactcaaccaagtcattctgagaatagtgtatgcggcgaccgagttgctcttgcccggcgtcaatacgggataataccgcgccacatagcagaactttaaaagtgctcatcattggaaaacgttcttcggggcgaaaactctcaaggatcttaccgctgttgagatccagttcgatgtaacccactcgtgcacccaactgatcttcagcatcttttactttcaccagcgtttctgggtgagcaaaaacaggaaggcaaaatgccgcaaaaaagggaataagggcgacacggaaatgttgaatactcatactcttcctttttcaatattattgaagcattt

Leu1frag

nmt81 prom

MCS

PK tag

nmt term

Ura4 frag

beta-lactamase (AmpR)

**pINTL41**

atcagggttattgtctcatgagcggatacatatttgaatgtatttagaaaaataaacaaataggggttccgcgcacatttccccgaaaagtgccacctgacgtctaaggcggccgcacattataatgcatattcgtactgtctattaaacatttttttgacattaaaaatttcgtttactaacgtagaaagcgctgataattgaggctactattttaatatctagaaacaaagattcattaattaattaagtaagctccaggatacttgtatatttcgttaaaaaagcgcaatttcaacaattcctatgaacatccatgatttcatcacaaaatcaaacatatcaggatttaacgtgaattatttgctttcttgtgattttcatctgatttctggtcatttacgttactgtaaacaagctttttaaaaaaaggattttttacgcaatattatcgatatcccaatctgtagtataaaaagtgcaacagtgttgagtttcccgaaaccaggcatttttgcggtagggtctgaaactttcacgaaaccatttcacaacaaaacttcttcaacaacatgtgtgcaaagaaaatcgtcgtcttaccaggagaccatattggccctgaaattgttgcttctgccttggaggttttgaaagtcgttgagaagaagcgacctgagttaaaactcgagtttgaagaacacaagattggaggtgcctctattgatgcctatggaacccctttgactgacgagactgtgaaggcttgtttggaagctgacggtgttcttttgggtgccgttggtggtcctgaatggaccaaccccaattgtcgtcctgagcaaggtttattgaagcttcgtaagagtatgggtgtttgggccaaccttcgaccttgcaactttgccagcaagtctttagtcaagtacagccctttgaagcctgaaatcgttgaaggtgtcgatttttgtgttgtacgagaacttactggaggttgttactttggtgagcgcactgaggacaacggatcgggttatgctatggacacttggccttacagtttggaagaagtttctcgtattgctcgtttggctgcttggttagctgaaacttccaaccctcctgctcccgtcacattactcgacaaagctaatgtctgcaggtcgatcgactctagaggatcagaaaattatcgccataaaagacagaataagtcatcagcggttgtttcatttcctatattttttttttatttttttattttttaataagggaaaatttaacgtctaaggatacagaagattgttagcacattaaagtaataaaggcttaagtagtaagtgccttagcatgttattgtatttcaaaggacataatctaaaataataacaatatcatttctcacaagttattcaattttcttttttttttctaataatatcaagaatgtattatttgtttgacataagtcaactaatttatttaatatgctggattaatcttgcagacatgtaaattaacaagttttagtcaaataacgttgaagtttcaatgaactcaaataatttctctttttttttatataaccataatctgatttatattttccgcagggatcaactgaagttatgacatttggattggatcacttataaccttggtcgccaaataatacaaaaatcagcgttataaaacaaagaaggtttttgttaagaaattaatcctctttcttgataagaaagttgaaccgaaattgcagatactgatatatgaaaataatacccacaattttgggaatagcgcaagcctcaatttaaacaataggtgaggacacatgataatgacctcaatgattgttagaagaaaagagcctcattacaaaatcgaaaaatgaatggttgggtacaagtttccaaaacatggtaaagtggactttgcgtatgagacgtaaatagaaaaaaacacttgttatatgttttctagaattattgttgtctctttatggttggatgatgcaaaatagtaatttcggttagttgctgtaaaacaccacgagacaaatagatatggatatttattaaatcaggaaaaacgtaactctcggctactggatggttcagtcacccaacgattactggggagagaaaacagggcaaaagcaaagcttaaaggaatccgattgtcattcggcaatgtgcagcgaaactaaaaaccggataatggacctgttaatcgaaacattgaagataaaggaagaggaatcctggcatatcatcaattgaataagttgaattaattatttcaatctcattctcactttctgacttatagtcgctttgttaaatcatatgtcgactctagaggatccccgggtaaaaggaatgtctcccttgccagtactgctagggtttttctttcaaactatggaagcccattcaagctgcatattacgattttgtttttcgcttttagaaagtggtttagatgagataatagaaaaattcttgatctccgacaacgagtacttttattttttttgctaatcactttactcaatattagctcgaaatcgtagaaacgtagacgggtgcgggataccgagtggtgtagttaagaatttttataaaccacgtggcccaaaaatatgaacccaaaacgtttatacatgagtatactttaagaaggctataccccttcgtgttagatgtagttttagctacccaacccgagtctatgagcttgacttcagatgtagaaggcattaaatcgttttgaatattaattaaaaaacgatgaaaattaaatatttaaaagcaatcatacgctgaaaatttagtgctgtggctaatccttcaacatggaaatgccataaaagtgactttgacaaaaaaaaaagtatatacaggtagtaaactcatctacttcattgactttgtttacagcatgtggaaggaggaatatttattgctaaatcgtagtttaacattcaataagtaatactattgaaattcgacaagattggccgcatggatgaaaaagaggcattttgctttgggagaattagtccaaattagaactgaaaaaaaaaactttacgaggcaaaaatgtcggattgagatcgtaaaagttcgctcgtcgtcttttgctttgtgattgttttcatggatacatcttgctggatatttaaattttagtactatgtataagatattctataaatgttttatcacccaaacctgttagcgccttcttaattctattcaatctggcttttgctctgagactacttcttggactttcactacttgttagttatacggaatttgtgtaattagaagtgaaataatcctttctattagtaatgcgagctcgatctacaaatcccactggctatatgtatgcatttgtgttaaaaaagtttgtatagattatttaatctactcagcattctttctctaaataggaatttgttacttaatggagaaaaaaatgtttcgatttacctagtgtatttgtttgtatactcacgtttaatttcaaacatccattctatcttgtgtaatttttggcatggtgaaaaagataatcagccttataatctttacaaaagtaagaaattctgtaaataagccttaatgcccttgctttaaattaaaatggttctttttcatgataatgtttgcactttgtgaatatattttagatagttctgtgaggtataattaagatgttttagagacttatacaattttgtctttataaattcttaattgattttaccatcccagtttaactatgcttcgtcggcatctctgcacatgtcgtgttttcttaccgtattgtcctaccaagaacctcttttttgcttggatcgaaattaaaggtttaaaagcaaagttatggatgctagagtatttcaaagctattcagctagagctgaggggatgaaaaatcccattgccaaggaattgttggctttgatggaagaaaagcaaagcaacttgtcagtcgcggtcgatttgacgaagaaatccgaaatcttagaattggtagataaaattggaccctatgtctgtgttatcaagacacatattgacgttgtcgaggatttcgaccaggatatggtagaaaaactggtggccttaggtaaaaagcatcgttttcttatctttgaggatcgcaaattcgcagacattggaaataccgtcaagctacaatatgcatctggtgtgtacaaaattgcttcttgggctcatatcacaaattgccatacagtgccaggcgagggtattatacaaggcctcaaagaagttggtttacctttgggacgtggtctcttgcttttggctgaaatgtcttccaaaggctctttggctactggttcctacacagagaaaaccttagaatggtttgagaagcataccgatttttgctttggctttatagctggtcgtcgatttcctaaccttcaaagcgactacataactatgtcccctggtatcggcttggatgttaaaggagacgggctgggacagcaatatcgtactcctgaagaagtgattgtaaactgcggtagcgatatcatcattgttggtcgtggagtctatggagctggtcgtaatcctgttgtcgaagccaagagatatagagaagctggttggaaggcatatcagcaaagactttctcagcattaaaaaaagactaatgtaaaatttttttggttggttattgaaaaagtcgatgccttgtttgcgtttgttttcctaggcgttttatgtcagaaggcatttagaattagtatacaagtactctttggtaaaattttatgtagcgactaaaatattaactattatagataaacaccttgggaataaaaagtaatttgctatagtaatttattaaacatgctcctacaacattaccacaatcttttctcttggattgacattgaataagaaaagagtgaatttttttagacttgtaatgataactatgtacaaagccaatgaaagatgtatgtagatgaatgtaaaataccatgtagacaaacaagataaaacttggttataaacattggtgttggaacagaataaattagatgtcaaaaagtttcgtcaatatcacgatttgatttgacctaatttttattgattaattgtgtcgcatatttattatcgttaaaaatgcaattgacaaaagcgtaaattttaaggctctaatgttttttcttttggtatgttatagtcaagcaaagatgtattaagttgcaaaagcatggaattgatttacgacatccgttcaactttatgaccgagttgtcgctgtgtacttactctttttttactatgaaaagtttcaaacataatatcattaattatttctttttaaacgaagtaacctgtaacataaataataggggaaaagaagaaaaaaaccataaaaattcgagaacgtaacaattattattgtaacgatgacaacccgattctttgagagagccagagacatgacttttgcattaccgtaattggtttctacttatgaattgtaaacttgtgtagggaatttcatccacagctatcgcgggctgctctctgcatcagtgttagcacattttatattaatgaagattttgctatgcaaaatacgtacatttctggcgcatatatttttatcaaacgaacatgactatcctttccactttgatggttcgcaagtattttgatttgtattaaatgcacatctaacaagccatttcgcccaaaatcaccaactggcacatgtacccaaccagttggttcctcgacttgaactgttgtaacaatttccaaatcttgaaaccctgtccgcggccgcccagctgcattaatgaatcggccaacgcgcggggagaggcggtttgcgtattgggcgctcttccgcttcctcgctcactgactcgctgcgctcggtcgttcggctgcggcgagcggtatcagctcactcaaaggcggtaatacggttatccacagaatcaggggataacgcaggaaagaacatgtgagcaaaaggccagcaaaaggccaggaaccgtaaaaaggccgcgttgctggcgtttttccataggctccgcccccctgacgagcatcacaaaaatcgacgctcaagtcagaggtggcgaaacccgacaggactataaagataccaggcgtttccccctggaagctccctcgtgcgctctcctgttccgaccctgccgcttaccggatacctgtccgcctttctcccttcgggaagcgtggcgctttctcatagctcacgctgtaggtatctcagttcggtgtaggtcgttcgctccaagctgggctgtgtgcacgaaccccccgttcagcccgaccgctgcgccttatccggtaactatcgtcttgagtccaacccggtaagacacgacttatcgccactggcagcagccactggtaacaggattagcagagcgaggtatgtaggcggtgctacagagttcttgaagtggtggcctaactacggctacactagaagaacagtatttggtatctgcgctctgctgaagccagttaccttcggaaaaagagttggtagctcttgatccggcaaacaaaccaccgctggtagcggtggtttttttgtttgcaagcagcagattacgcgcagaaaaaaaggatctcaagaagatcctttgatcttttctacggggtctgacgctcagtggaacgaaaactcacgttaagggattttggtcatgagattatcaaaaaggatcttcacctagatccttttaaattaaaaatgaagttttaaatcaatctaaagtatatatgagtaaacttggtctgacagttaccaatgcttaatcagtgaggcacctatctcagcgatctgtctatttcgttcatccatagttgcctgactccccgtcgtgtagataactacgatacgggagggcttaccatctggccccagtgctgcaatgataccgcgagacccacgctcaccggctccagatttatcagcaataaaccagccagccggaagggccgagcgcagaagtggtcctgcaactttatccgcctccatccagtctattaattgttgccgggaagctagagtaagtagttcgccagttaatagtttgcgcaacgttgttgccattgctacaggcatcgtggtgtcacgctcgtcgtttggtatggcttcattcagctccggttcccaacgatcaaggcgagttacatgatcccccatgttgtgcaaaaaagcggttagctccttcggtcctccgatcgttgtcagaagtaagttggccgcagtgttatcactcatggttatggcagcactgcataattctcttactgtcatgccatccgtaagatgcttttctgtgactggtgagtactcaaccaagtcattctgagaatagtgtatgcggcgaccgagttgctcttgcccggcgtcaatacgggataataccgcgccacatagcagaactttaaaagtgctcatcattggaaaacgttcttcggggcgaaaactctcaaggatcttaccgctgttgagatccagttcgatgtaacccactcgtgcacccaactgatcttcagcatcttttactttcaccagcgtttctgggtgagcaaaaacaggaaggcaaaatgccgcaaaaaagggaataagggcgacacggaaatgttgaatactcatactcttcctttttcaatattattgaagcattt

Leu1 frag

nmt41 prom

MCS

nmt term

Ura4 frag

beta-lactamase (AmpR)

**pINTL41PKN**

atcagggttattgtctcatgagcggatacatatttgaatgtatttagaaaaataaacaaataggggttccgcgcacatttccccgaaaagtgccacctgacgtctaaggcggccgcacattataatgcatattcgtactgtctattaaacatttttttgacattaaaaatttcgtttactaacgtagaaagcgctgataattgaggctactattttaatatctagaaacaaagattcattaattaattaagtaagctccaggatacttgtatatttcgttaaaaaagcgcaatttcaacaattcctatgaacatccatgatttcatcacaaaatcaaacatatcaggatttaacgtgaattatttgctttcttgtgattttcatctgatttctggtcatttacgttactgtaaacaagctttttaaaaaaaggattttttacgcaatattatcgatatcccaatctgtagtataaaaagtgcaacagtgttgagtttcccgaaaccaggcatttttgcggtagggtctgaaactttcacgaaaccatttcacaacaaaacttcttcaacaacatgtgtgcaaagaaaatcgtcgtcttaccaggagaccatattggccctgaaattgttgcttctgccttggaggttttgaaagtcgttgagaagaagcgacctgagttaaaactcgagtttgaagaacacaagattggaggtgcctctattgatgcctatggaacccctttgactgacgagactgtgaaggcttgtttggaagctgacggtgttcttttgggtgccgttggtggtcctgaatggaccaaccccaattgtcgtcctgagcaaggtttattgaagcttcgtaagagtatgggtgtttgggccaaccttcgaccttgcaactttgccagcaagtctttagtcaagtacagccctttgaagcctgaaatcgttgaaggtgtcgatttttgtgttgtacgagaacttactggaggttgttactttggtgagcgcactgaggacaacggatcgggttatgctatggacacttggccttacagtttggaagaagtttctcgtattgctcgtttggctgcttggttagctgaaacttccaaccctcctgctcccgtcacattactcgacaaagctaatgtctgcaggtcgatcgactctagaggatcagaaaattatcgccataaaagacagaataagtcatcagcggttgtttcatttcctatattttttttttatttttttattttttaataagggaaaatttaacgtctaaggatacagaagattgttagcacattaaagtaataaaggcttaagtagtaagtgccttagcatgttattgtatttcaaaggacataatctaaaataataacaatatcatttctcacaagttattcaattttcttttttttttctaataatatcaagaatgtattatttgtttgacataagtcaactaatttatttaatatgctggattaatcttgcagacatgtaaattaacaagttttagtcaaataacgttgaagtttcaatgaactcaaataatttctctttttttttatataaccataatctgatttatattttccgcagggatcaactgaagttatgacatttggattggatcacttataaccttggtcgccaaataatacaaaaatcagcgttataaaacaaagaaggtttttgttaagaaattaatcctctttcttgataagaaagttgaaccgaaattgcagatactgatatatgaaaataatacccacaattttgggaatagcgcaagcctcaatttaaacaataggtgaggacacatgataatgacctcaatgattgttagaagaaaagagcctcattacaaaatcgaaaaatgaatggttgggtacaagtttccaaaacatggtaaagtggactttgcgtatgagacgtaaatagaaaaaaacacttgttatatgttttctagaattattgttgtctctttatggttggatgatgcaaaatagtaatttcggttagttgctgtaaaacaccacgagacaaatagatatggatatttattaaatcaggaaaaacgtaactctcggctactggatggttcagtcacccaacgattactggggagagaaaacagggcaaaagcaaagcttaaaggaatccgattgtcattcggcaatgtgcagcgaaactaaaaaccggataatggacctgttaatcgaaacattgaagataaaggaagaggaatcctggcatatcatcaattgaataagttgaattaattatttcaatctcattctcactttctgacttatagtcgctttgttaaatcatatcgagctcatgggtattcctaaccctttgcttggtcttgatggtattcctaaccctttgcttggtcttgattccatgggtattcctaaccctttgcttggtcttgatcatatgtcgacaggtaccagatctcgagggatccccgggtaaaaggaatgtctcccttgccagtactgctagggtttttctttcaaactatggaagcccattcaagctgcatattacgattttgtttttcgcttttagaaagtggtttagatgagataatagaaaaattcttgatctccgacaacgagtacttttattttttttgctaatcactttactcaatattagctcgaaatcgtagaaacgtagacgggtgcgggataccgagtggtgtagttaagaatttttataaaccacgtggcccaaaaatatgaacccaaaacgtttatacatgagtatactttaagaaggctataccccttcgtgttagatgtagttttagctacccaacccgagtctatgagcttgacttcagatgtagaaggcattaaatcgttttgaatattaattaaaaaacgatgaaaattaaatatttaaaagcaatcatacgctgaaaatttagtgctgtggctaatccttcaacatggaaatgccataaaagtgactttgacaaaaaaaaaagtatatacaggtagtaaactcatctacttcattgactttgtttacagcatgtggaaggaggaatatttattgctaaatcgtagtttaacattcaataagtaatactattgaaattcgacaagattggccgcatggatgaaaaagaggcattttgctttgggagaattagttcaaattagaactgaaaaaaaaaactttacgaggcaaaaatgtcggattgagatcgtaaaagttcgctcgtcgtcttttgctttgtgattgttttcatggatacatcttgctggatatttaaattttagtactatgtataagatattctataaatgttttatcacccaaacctgttagcgccttcttaattctattcaatctggcttttgctctgagactacttcttggactttcactacttgttagttatacggaatttgtgtaattagaagtgaaataatcctttctattagtaatgcgagctcgatctacaaatcccactggctatatgtatgcatttgtgttaaaaaagtttgtatagattatttaatctactcagcattctttctctaaataggaatttgttacttaatggagaaaaaaatgtttcgatttacctagtgtatttgtttgtatactcacgtttaatttcaaacatccattctatcttgtgtaatttttggcatggtgaaaaagataatcagccttataatctttacaaaagtaagaaattctgtaaataagccttaatgcccttgctttaaattaaaatggttctttttcatgataatgtttgcactttgtgaatatattttagatagttctgtgaggtataattaagatgttttagagacttatacaattttgtctttataaattcttaattgattttaccatcccagtttaactatgcttcgtcggcatctctgcacatgtcgtgttttcttaccgtattgtcctaccaagaacctcttttttgcttggatcgaaattaaaggtttaaaagcaaagttatggatgctagagtatttcaaagctattcagctagagctgaggggatgaaaaatcccattgccaaggaattgttggctttgatggaagaaaagcaaagcaacttgtcagtcgcggtcgatttgacgaagaaatccgaaatcttagaattggtagataaaattggaccctatgtctgtgttatcaagacacatattgacgttgtcgaggatttcgaccaggatatggtagaaaaactggtggccttaggtaaaaagcatcgttttcttatctttgaggatcgcaaattcgcagacattggaaataccgtcaagctacaatatgcatctggtgtgtacaaaattgcttcttgggctcatatcacaaattgccatacagtgccaggcgagggtattatacaaggcctcaaagaagttggtttacctttgggacgtggtctcttgcttttggctgaaatgtcttccaaaggctctttggctactggttcctacacagagaaaaccttagaatggtttgagaagcataccgatttttgctttggctttatagctggtcgtcgatttcctaaccttcaaagcgactacataactatgtcccctggtatcggcttggatgttaaaggagacgggctgggacagcaatatcgtactcctgaagaagtgattgtaaactgcggtagcgatatcatcattgttggtcgtggagtctatggagctggtcgtaatcctgttgtcgaagccaagagatatagagaagctggttggaaggcatatcagcaaagactttctcagcattaaaaaaagactaatgtaaaatttttttggttggttattgaaaaagtcgatgccttgtttgcgtttgttttcctaggcgttttatgtcagaaggcatttagaattagtatacaagtactctttggtaaaattttatgtagcgactaaaatattaactattatagataaacaccttgggaataaaaagtaatttgctatagtaatttattaaacatgctcctacaacattaccacaatcttttctcttggattgacattgaataagaaaagagtgaatttttttagacttgtaatgataactatgtacaaagccaatgaaagatgtatgtagatgaatgtaaaataccatgtagacaaacaagataaaacttggttataaacattggtgttggaacagaataaattagatgtcaaaaagtttcgtcaatatcacgatttgatttgacctaatttttattgattaattgtgtcgcatatttattatcgttaaaaatgcaattgacaaaagcgtaaattttaaggctctaatgttttttcttttggtatgttatagtcaagcaaagatgtattaagttgcaaaagcatggaattgatttacgacatccgttcaactttatgaccgagttgtcgctgtgtacttactctttttttactatgaaaagtttcaaacataatatcattaattatttctttttaaacgaagtaacctgtaacataaataataggggaaaagaagaaaaaaaccataaaaattcgagaacgtaacaattattattgtaacgatgacaacccgattctttgagagagccagagacatgacttttgcattaccgtaattggtttctacttatgaattgtaaacttgtgtagggaatttcatccacagctatcgcgggctgctctctgcatcagtgttagcacattttatattaatgaagattttgctatgcaaaatacgtacatttctggcgcatatatttttatcaaacgaacatgactatcctttccactttgatggttcgcaagtattttgatttgtattaaatgcacatctaacaagccatttcgcccaaaatcaccaactggcacatgtacccaaccagttggttcctcgacttgaactgttgtaacaatttccaaatcttgaaaccctgtccgcggccgcccagctgcattaatgaatcggccaacgcgcggggagaggcggtttgcgtattgggcgctcttccgcttcctcgctcactgactcgctgcgctcggtcgttcggctgcggcgagcggtatcagctcactcaaaggcggtaatacggttatccacagaatcaggggataacgcaggaaagaacatgtgagcaaaaggccagcaaaaggccaggaaccgtaaaaaggccgcgttgctggcgtttttccataggctccgcccccctgacgagcatcacaaaaatcgacgctcaagtcagaggtggcgaaacccgacaggactataaagataccaggcgtttccccctggaagctccctcgtgcgctctcctgttccgaccctgccgcttaccggatacctgtccgcctttctcccttcgggaagcgtggcgctttctcatagctcacgctgtaggtatctcagttcggtgtaggtcgttcgctccaagctgggctgtgtgcacgaaccccccgttcagcccgaccgctgcgccttatccggtaactatcgtcttgagtccaacccggtaagacacgacttatcgccactggcagcagccactggtaacaggattagcagagcgaggtatgtaggcggtgctacagagttcttgaagtggtggcctaactacggctacactagaagaacagtatttggtatctgcgctctgctgaagccagttaccttcggaaaaagagttggtagctcttgatccggcaaacaaaccaccgctggtagcggtggtttttttgtttgcaagcagcagattacgcgcagaaaaaaaggatctcaagaagatcctttgatcttttctacggggtctgacgctcagtggaacgaaaactcacgttaagggattttggtcatgagattatcaaaaaggatcttcacctagatccttttaaattaaaaatgaagttttaaatcaatctaaagtatatatgagtaaacttggtctgacagttaccaatgcttaatcagtgaggcacctatctcagcgatctgtctatttcgttcatccatagttgcctgactccccgtcgtgtagataactacgatacgggagggcttaccatctggccccagtgctgcaatgataccgcgagacccacgctcaccggctccagatttatcagcaataaaccagccagccggaagggccgagcgcagaagtggtcctgcaactttatccgcctccatccagtctattaattgttgccgggaagctagagtaagtagttcgccagttaatagtttgcgcaacgttgttgccattgctacaggcatcgtggtgtcacgctcgtcgtttggtatggcttcattcagctccggttcccaacgatcaaggcgagttacatgatcccccatgttgtgcaaaaaagcggttagctccttcggtcctccgatcgttgtcagaagtaagttggccgcagtgttatcactcatggttatggcagcactgcataattctcttactgtcatgccatccgtaagatgcttttctgtgactggtgagtactcaaccaagtcattctgagaatagtgtatgcggcgaccgagttgctcttgcccggcgtcaatacgggataataccgcgccacatagcagaactttaaaagtgctcatcattggaaaacgttcttcggggcgaaaactctcaaggatcttaccgctgttgagatccagttcgatgtaacccactcgtgcacccaactgatcttcagcatcttttactttcaccagcgtttctgggtgagcaaaaacaggaaggcaaaatgccgcaaaaaagggaataagggcgacacggaaatgttgaatactcatactcttcctttttcaatattattgaagcattt

Leu1 frag

nmt41 prom

MCS

PK tag

nmt term

Ura4 frag

beta-lactamase (AmpR)

**pINTL41PKC**

atcagggttattgtctcatgagcggatacatatttgaatgtatttagaaaaataaacaaataggggttccgcgcacatttccccgaaaagtgccacctgacgtctaaggcggccgcacattataatgcatattcgtactgtctattaaacatttttttgacattaaaaatttcgtttactaacgtagaaagcgctgataattgaggctactattttaatatctagaaacaaagattcattaattaattaagtaagctccaggatacttgtatatttcgttaaaaaagcgcaatttcaacaattcctatgaacatccatgatttcatcacaaaatcaaacatatcaggatttaacgtgaattatttgctttcttgtgattttcatctgatttctggtcatttacgttactgtaaacaagctttttaaaaaaaggattttttacgcaatattatcgatatcccaatctgtagtataaaaagtgcaacagtgttgagtttcccgaaaccaggcatttttgcggtagggtctgaaactttcacgaaaccatttcacaacaaaacttcttcaacaacatgtgtgcaaagaaaatcgtcgtcttaccaggagaccatattggccctgaaattgttgcttctgccttggaggttttgaaagtcgttgagaagaagcgacctgagttaaaactcgagtttgaagaacacaagattggaggtgcctctattgatgcctatggaacccctttgactgacgagactgtgaaggcttgtttggaagctgacggtgttcttttgggtgccgttggtggtcctgaatggaccaaccccaattgtcgtcctgagcaaggtttattgaagcttcgtaagagtatgggtgtttgggccaaccttcgaccttgcaactttgccagcaagtctttagtcaagtacagccctttgaagcctgaaatcgttgaaggtgtcgatttttgtgttgtacgagaacttactggaggttgttactttggtgagcgcactgaggacaacggatcgggttatgctatggacacttggccttacagtttggaagaagtttctcgtattgctcgtttggctgcttggttagctgaaacttccaaccctcctgctcccgtcacattactcgacaaagctaatgtctgcaggtcgatcgactctagaggatcagaaaattatcgccataaaagacagaataagtcatcagcggttgtttcatttcctatattttttttttatttttttattttttaataagggaaaatttaacgtctaaggatacagaagattgttagcacattaaagtaataaaggcttaagtagtaagtgccttagcatgttattgtatttcaaaggacataatctaaaataataacaatatcatttctcacaagttattcaattttcttttttttttctaataatatcaagaatgtattatttgtttgacataagtcaactaatttatttaatatgctggattaatcttgcagacatgtaaattaacaagttttagtcaaataacgttgaagtttcaatgaactcaaataatttctctttttttttatataaccataatctgatttatattttccgcagggatcaactgaagttatgacatttggattggatcacttataaccttggtcgccaaataatacaaaaatcagcgttataaaacaaagaaggtttttgttaagaaattaatcctctttcttgataagaaagttgaaccgaaattgcagatactgatatatgaaaataatacccacaattttgggaatagcgcaagcctcaatttaaacaataggtgaggacacatgataatgacctcaatgattgttagaagaaaagagcctcattacaaaatcgaaaaatgaatggttgggtacaagtttccaaaacatggtaaagtggactttgcgtatgagacgtaaatagaaaaaaacacttgttatatgttttctagaattattgttgtctctttatggttggatgatgcaaaatagtaatttcggttagttgctgtaaaacaccacgagacaaatagatatggatatttattaaatcaggaaaaacgtaactctcggctactggatggttcagtcacccaacgattactggggagagaaaacagggcaaaagcaaagcttaaaggaatccgattgtcattcggcaatgtgcagcgaaactaaaaaccggataatggacctgttaatcgaaacattgaagataaaggaagaggaatcctggcatatcatcaattgaataagttgaattaattatttcaatctcattctcactttctgacttatagtcgctttgttaaatcatatgtcgacatggtaccagatctctcgaggatccccgggtatgggtattcctaaccctttgcttggtcttgatggtattcctaaccctttgcttggtcttgattccatgggtattcctaaccctttgcttggtcttgatcattaaggatcgggtaaaaggaatgtctcccttgccagtactgctagggtttttctttcaaactatggaagcccattcaagctgcatattacgattttgtttttcgcttttagaaagtggtttagatgagataatagaaaaattcttgatctccgacaacgagtacttttattttttttgctaatcactttactcaatattagctcgaaatcgtagaaacgtagacgggtgcgggataccgagtggtgtagttaagaatttttataaaccacgtggcccaaaaatatgaacccaaaacgtttatacatgagtatactttaagaaggctataccccttcgtgttagatgtagttttagctacccaacccgagtctatgagcttgacttcagatgtagaaggcattaaatcgttttgaatattaattaaaaaacgatgaaaattaaatatttaaaagcaatcatacgctgaaaatttagtgctgtggctaatccttcaacatggaaatgccataaaagtgactttgacaaaaaaaaaagtatatacaggtagtaaactcatctacttcattgactttgtttacagcatgtggaaggaggaatatttattgctaaatcgtagtttaacattcaataagtaatactattgaaattcgacaagattggccgcatggatgaaaaagaggcattttgctttgggagaattagtccaaattagaactgaaaaaaaaaactttacgaggcaaaaatgtcggattgagatcgtaaaagttcgctcgtcgtcttttgctttgtgattgttttcatggatacatcttgctggatatttaaattttagtactatgtataagatattctataaatgttttatcacccaaacctgttagcgccttcttaattctattcaatctggcttttgctctgagactacttcttggactttcactacttgttagttatacggaatttgtgtaattagaagtgaaataatcctttctattagtaatgcgagctcgatctacaaatcccactggctatatgtatgcatttgtgttaaaaaagtttgtatagattatttaatctactcagcattctttctctaaataggaatttgttacttaatggagaaaaaaatgtttcgatttacctagtgtatttgtttgtatactcacgtttaatttcaaacatccattctatcttgtgtaatttttggcatggtgaaaaagataatcagccttataatctttacaaaagtaagaaattctgtaaataagccttaatgcccttgctttaaattaaaatggttctttttcatgataatgtttgcactttgtgaatatattttagatagttctgtgaggtataattaagatgttttagagacttatacaattttgtctttataaattcttaattgattttaccatcccagtttaactatgcttcgtcggcatctctgcacatgtcgtgttttcttaccgtattgtcctaccaagaacctcttttttgcttggatcgaaattaaaggtttaaaagcaaagttatggatgctagagtatttcaaagctattcagctagagctgaggggatgaaaaatcccattgccaaggaattgttggctttgatggaagaaaagcaaagcaacttgtcagtcgcggtcgatttgacgaagaaatccgaaatcttagaattggtagataaaattggaccctatgtctgtgttatcaagacacatattgacgttgtcgaggatttcgaccaggatatggtagaaaaactggtggccttaggtaaaaagcatcgttttcttatctttgaggatcgcaaattcgcagacattggaaataccgtcaagctacaatatgcatctggtgtgtacaaaattgcttcttgggctcatatcacaaattgccatacagtgccaggcgagggtattatacaaggcctcaaagaagttggtttacctttgggacgtggtctcttgcttttggctgaaatgtcttccaaaggctctttggctactggttcctacacagagaaaaccttagaatggtttgagaagcataccgatttttgctttggctttatagctggtcgtcgatttcctaaccttcaaagcgactacataactatgtcccctggtatcggcttggatgttaaaggagacgggctgggacagcaatatcgtactcctgaagaagtgattgtaaactgcggtagcgatatcatcattgttggtcgtggagtctatggagctggtcgtaatcctgttgtcgaagccaagagatatagagaagctggttggaaggcatatcagcaaagactttctcagcattaaaaaaagactaatgtaaaatttttttggttggttattgaaaaagtcgatgccttgtttgcgtttgttttcctaggcgttttatgtcagaaggcatttagaattagtatacaagtactctttggtaaaattttatgtagcgactaaaatattaactattatagataaacaccttgggaataaaaagtaatttgctatagtaatttattaaacatgctcctacaacattaccacaatcttttctcttggattgacattgaataagaaaagagtgaatttttttagacttgtaatgataactatgtacaaagccaatgaaagatgtatgtagatgaatgtaaaataccatgtagacaaacaagataaaacttggttataaacattggtgttggaacagaataaattagatgtcaaaaagtttcgtcaatatcacgatttgatttgacctaatttttattgattaattgtgtcgcatatttattatcgttaaaaatgcaattgacaaaagcgtaaattttaaggctctaatgttttttcttttggtatgttatagtcaagcaaagatgtattaagttgcaaaagcatggaattgatttacgacatccgttcaactttatgaccgagttgtcgctgtgtacttactctttttttactatgaaaagtttcaaacataatatcattaattatttctttttaaacgaagtaacctgtaacataaataataggggaaaagaagaaaaaaaccataaaaattcgagaacgtaacaattattattgtaacgatgacaacccgattctttgagagagccagagacatgacttttgcattaccgtaattggtttctacttatgaattgtaaacttgtgtagggaatttcatccacagctatcgcgggctgctctctgcatcagtgttagcacattttatattaatgaagattttgctatgcaaaatacgtacatttctggcgcatatatttttatcaaacgaacatgactatcctttccactttgatggttcgcaagtattttgatttgtattaaatgcacatctaacaagccatttcgcccaaaatcaccaactggcacatgtacccaaccagttggttcctcgacttgaactgttgtaacaatttccaaatcttgaaaccctgtccgcggccgcccagctgcattaatgaatcggccaacgcgcggggagaggcggtttgcgtattgggcgctcttccgcttcctcgctcactgactcgctgcgctcggtcgttcggctgcggcgagcggtatcagctcactcaaaggcggtaatacggttatccacagaatcaggggataacgcaggaaagaacatgtgagcaaaaggccagcaaaaggccaggaaccgtaaaaaggccgcgttgctggcgtttttccataggctccgcccccctgacgagcatcacaaaaatcgacgctcaagtcagaggtggcgaaacccgacaggactataaagataccaggcgtttccccctggaagctccctcgtgcgctctcctgttccgaccctgccgcttaccggatacctgtccgcctttctcccttcgggaagcgtggcgctttctcatagctcacgctgtaggtatctcagttcggtgtaggtcgttcgctccaagctgggctgtgtgcacgaaccccccgttcagcccgaccgctgcgccttatccggtaactatcgtcttgagtccaacccggtaagacacgacttatcgccactggcagcagccactggtaacaggattagcagagcgaggtatgtaggcggtgctacagagttcttgaagtggtggcctaactacggctacactagaagaacagtatttggtatctgcgctctgctgaagccagttaccttcggaaaaagagttggtagctcttgatccggcaaacaaaccaccgctggtagcggtggtttttttgtttgcaagcagcagattacgcgcagaaaaaaaggatctcaagaagatcctttgatcttttctacggggtctgacgctcagtggaacgaaaactcacgttaagggattttggtcatgagattatcaaaaaggatcttcacctagatccttttaaattaaaaatgaagttttaaatcaatctaaagtatatatgagtaaacttggtctgacagttaccaatgcttaatcagtgaggcacctatctcagcgatctgtctatttcgttcatccatagttgcctgactccccgtcgtgtagataactacgatacgggagggcttaccatctggccccagtgctgcaatgataccgcgagacccacgctcaccggctccagatttatcagcaataaaccagccagccggaagggccgagcgcagaagtggtcctgcaactttatccgcctccatccagtctattaattgttgccgggaagctagagtaagtagttcgccagttaatagtttgcgcaacgttgttgccattgctacaggcatcgtggtgtcacgctcgtcgtttggtatggcttcattcagctccggttcccaacgatcaaggcgagttacatgatcccccatgttgtgcaaaaaagcggttagctccttcggtcctccgatcgttgtcagaagtaagttggccgcagtgttatcactcatggttatggcagcactgcataattctcttactgtcatgccatccgtaagatgcttttctgtgactggtgagtactcaaccaagtcattctgagaatagtgtatgcggcgaccgagttgctcttgcccggcgtcaatacgggataataccgcgccacatagcagaactttaaaagtgctcatcattggaaaacgttcttcggggcgaaaactctcaaggatcttaccgctgttgagatccagttcgatgtaacccactcgtgcacccaactgatcttcagcatcttttactttcaccagcgtttctgggtgagcaaaaacaggaaggcaaaatgccgcaaaaaagggaataagggcgacacggaaatgttgaatactcatactcttcctttttcaatattattgaagcattt

Leu1 frag

nmt41 prom

MCS

PK tag

nmt term

Ura4 frag

beta-lactamase (AmpR)

**pINTL41HAN**

atcagggttattgtctcatgagcggatacatatttgaatgtatttagaaaaataaacaaataggggttccgcgcacatttccccgaaaagtgccacctgacgtctaaggcggccgcacattataatgcatattcgtactgtctattaaacatttttttgacattaaaaatttcgtttactaacgtagaaagcgctgataattgaggctactattttaatatctagaaacaaagattcattaattaattaagtaagctccaggatacttgtatatttcgttaaaaaagcgcaatttcaacaattcctatgaacatccatgatttcatcacaaaatcaaacatatcaggatttaacgtgaattatttgctttcttgtgattttcatctgatttctggtcatttacgttactgtaaacaagctttttaaaaaaaggattttttacgcaatattatcgatatcccaatctgtagtataaaaagtgcaacagtgttgagtttcccgaaaccaggcatttttgcggtagggtctgaaactttcacgaaaccatttcacaacaaaacttcttcaacaacatgtgtgcaaagaaaatcgtcgtcttaccaggagaccatattggccctgaaattgttgcttctgccttggaggttttgaaagtcgttgagaagaagcgacctgagttaaaactcgagtttgaagaacacaagattggaggtgcctctattgatgcctatggaacccctttgactgacgagactgtgaaggcttgtttggaagctgacggtgttcttttgggtgccgttggtggtcctgaatggaccaaccccaattgtcgtcctgagcaaggtttattgaagcttcgtaagagtatgggtgtttgggccaaccttcgaccttgcaactttgccagcaagtctttagtcaagtacagccctttgaagcctgaaatcgttgaaggtgtcgatttttgtgttgtacgagaacttactggaggttgttactttggtgagcgcactgaggacaacggatcgggttatgctatggacacttggccttacagtttggaagaagtttctcgtattgctcgtttggctgcttggttagctgaaacttccaaccctcctgctcccgtcacattactcgacaaagctaatgtctgcaggtcgatcgactctagaggatcagaaaattatcgccataaaagacagaataagtcatcagcggttgtttcatttcctatattttttttttatttttttattttttaataagggaaaatttaacgtctaaggatacagaagattgttagcacattaaagtaataaaggcttaagtagtaagtgccttagcatgttattgtatttcaaaggacataatctaaaataataacaatatcatttctcacaagttattcaattttcttttttttttctaataatatcaagaatgtattatttgtttgacataagtcaactaatttatttaatatgctggattaatcttgcagacatgtaaattaacaagttttagtcaaataacgttgaagtttcaatgaactcaaataatttctctttttttttatataaccataatctgatttatattttccgcagggatcaactgaagttatgacatttggattggatcacttataaccttggtcgccaaataatacaaaaatcagcgttataaaacaaagaaggtttttgttaagaaattaatcctctttcttgataagaaagttgaaccgaaattgcagatactgatatatgaaaataatacccacaattttgggaatagcgcaagcctcaatttaaacaataggtgaggacacatgataatgacctcaatgattgttagaagaaaagagcctcattacaaaatcgaaaaatgaatggttgggtacaagtttccaaaacatggtaaagtggactttgcgtatgagacgtaaatagaaaaaaacacttgttatatgttttctagaattattgttgtctctttatggttggatgatgcaaaatagtaatttcggttagttgctgtaaaacaccacgagacaaatagatatggatatttattaaatcaggaaaaacgtaactctcggctactggatggttcagtcacccaacgattactggggagagaaaacagggcaaaagcaaagcttaaaggaatccgattgtcattcggcaatgtgcagcgaaactaaaaaccggataatggacctgttaatcgaaacattgaagataaaggaagaggaatcctggcatatcatcaattgaataagttgaattaattatttcaatctcattctcactttctgacttatagtcgctttgttaaatcccatggcatacccttacgatgttcctgactatgcgggctatccctatgacgtcccggactatgccatgggctacccttacgacgttccagattacgctcatatgtcgactctagaggatccccgggtaaaaggaatgtctcccttgccagtactgctagggtttttctttcaaactatggaagcccattcaagctgcatattacgattttgtttttcgcttttagaaagtggtttagatgagataatagaaaaattcttgatctccgacaacgagtacttttattttttttgctaatcactttactcaatattagctcgaaatcgtagaaacgtagacgggtgcgggataccgagtggtgtagttaagaatttttataaaccacgtggcccaaaaatatgaacccaaaacgtttatacatgagtatactttaagaaggctataccccttcgtgttagatgtagttttagctacccaacccgagtctatgagcttgacttcagatgtagaaggcattaaatcgttttgaatattaattaaaaaacgatgaaaattaaatatttaaaagcaatcatacgctgaaaatttagtgctgtggctaatccttcaacatggaaatgccataaaagtgactttgacaaaaaaaaaagtatatacaggtagtaaactcatctacttcattgactttgtttacagcatgtggaaggaggaatatttattgctaaatcgtagtttaacattcaataagtaatactattgaaattcgacaagattggccgcatggatgaaaaagaggcattttgctttgggagaattagtccaaattagaactgaaaaaaaaaactttacgaggcaaaaatgtcggattgagatcgtaaaagttcgctcgtcgtcttttgctttgtgattgttttcatggatacatcttgctggatatttaaattttagtactatgtataagatattctataaatgttttatcacccaaacctgttagcgccttcttaattctattcaatctggcttttgctctgagactacttcttggactttcactacttgttagttatacggaatttgtgtaattagaagtgaaataatcctttctattagtaatgcgagctcgatctacaaatcccactggctatatgtatgcatttgtgttaaaaaagtttgtatagattatttaatctactcagcattctttctctaaataggaatttgttacttaatggagaaaaaaatgtttcgatttacctagtgtatttgtttgtatactcacgtttaatttcaaacatccattctatcttgtgtaatttttggcatggtgaaaaagataatcagccttataatctttacaaaagtaagaaattctgtaaataagccttaatgcccttgctttaaattaaaatggttctttttcatgataatgtttgcactttgtgaatatattttagatagttctgtgaggtataattaagatgttttagagacttatacaattttgtctttataaattcttaattgattttaccatcccagtttaactatgcttcgtcggcatctctgcacatgtcgtgttttcttaccgtattgtcctaccaagaacctcttttttgcttggatcgaaattaaaggtttaaaagcaaagttatggatgctagagtatttcaaagctattcagctagagctgaggggatgaaaaatcccattgccaaggaattgttggctttgatggaagaaaagcaaagcaacttgtcagtcgcggtcgatttgacgaagaaatccgaaatcttagaattggtagataaaattggaccctatgtctgtgttatcaagacacatattgacgttgtcgaggatttcgaccaggatatggtagaaaaactggtggccttaggtaaaaagcatcgttttcttatctttgaggatcgcaaattcgcagacattggaaataccgtcaagctacaatatgcatctggtgtgtacaaaattgcttcttgggctcatatcacaaattgccatacagtgccaggcgagggtattatacaaggcctcaaagaagttggtttacctttgggacgtggtctcttgcttttggctgaaatgtcttccaaaggctctttggctactggttcctacacagagaaaaccttagaatggtttgagaagcataccgatttttgctttggctttatagctggtcgtcgatttcctaaccttcaaagcgactacataactatgtcccctggtatcggcttggatgttaaaggagacgggctgggacagcaatatcgtactcctgaagaagtgattgtaaactgcggtagcgatatcatcattgttggtcgtggagtctatggagctggtcgtaatcctgttgtcgaagccaagagatatagagaagctggttggaaggcatatcagcaaagactttctcagcattaaaaaaagactaatgtaaaatttttttggttggttattgaaaaagtcgatgccttgtttgcgtttgttttcctaggcgttttatgtcagaaggcatttagaattagtatacaagtactctttggtaaaattttatgtagcgactaaaatattaactattatagataaacaccttgggaataaaaagtaatttgctatagtaatttattaaacatgctcctacaacattaccacaatcttttctcttggattgacattgaataagaaaagagtgaatttttttagacttgtaatgataactatgtacaaagccaatgaaagatgtatgtagatgaatgtaaaataccatgtagacaaacaagataaaacttggttataaacattggtgttggaacagaataaattagatgtcaaaaagtttcgtcaatatcacgatttgatttgacctaatttttattgattaattgtgtcgcatatttattatcgttaaaaatgcaattgacaaaagcgtaaattttaaggctctaatgttttttcttttggtatgttatagtcaagcaaagatgtattaagttgcaaaagcatggaattgatttacgacatccgttcaactttatgaccgagttgtcgctgtgtacttactctttttttactatgaaaagtttcaaacataatatcattaattatttctttttaaacgaagtaacctgtaacataaataataggggaaaagaagaaaaaaaccataaaaattcgagaacgtaacaattattattgtaacgatgacaacccgattctttgagagagccagagacatgacttttgcattaccgtaattggtttctacttatgaattgtaaacttgtgtagggaatttcatccacagctatcgcgggctgctctctgcatcagtgttagcacattttatattaatgaagattttgctatgcaaaatacgtacatttctggcgcatatatttttatcaaacgaacatgactatcctttccactttgatggttcgcaagtattttgatttgtattaaatgcacatctaacaagccatttcgcccaaaatcaccaactggcacatgtacccaaccagttggttcctcgacttgaactgttgtaacaatttccaaatcttgaaaccctgtccgcggccgcccagctgcattaatgaatcggccaacgcgcggggagaggcggtttgcgtattgggcgctcttccgcttcctcgctcactgactcgctgcgctcggtcgttcggctgcggcgagcggtatcagctcactcaaaggcggtaatacggttatccacagaatcaggggataacgcaggaaagaacatgtgagcaaaaggccagcaaaaggccaggaaccgtaaaaaggccgcgttgctggcgtttttccataggctccgcccccctgacgagcatcacaaaaatcgacgctcaagtcagaggtggcgaaacccgacaggactataaagataccaggcgtttccccctggaagctccctcgtgcgctctcctgttccgaccctgccgcttaccggatacctgtccgcctttctcccttcgggaagcgtggcgctttctcatagctcacgctgtaggtatctcagttcggtgtaggtcgttcgctccaagctgggctgtgtgcacgaaccccccgttcagcccgaccgctgcgccttatccggtaactatcgtcttgagtccaacccggtaagacacgacttatcgccactggcagcagccactggtaacaggattagcagagcgaggtatgtaggcggtgctacagagttcttgaagtggtggcctaactacggctacactagaagaacagtatttggtatctgcgctctgctgaagccagttaccttcggaaaaagagttggtagctcttgatccggcaaacaaaccaccgctggtagcggtggtttttttgtttgcaagcagcagattacgcgcagaaaaaaaggatctcaagaagatcctttgatcttttctacggggtctgacgctcagtggaacgaaaactcacgttaagggattttggtcatgagattatcaaaaaggatcttcacctagatccttttaaattaaaaatgaagttttaaatcaatctaaagtatatatgagtaaacttggtctgacagttaccaatgcttaatcagtgaggcacctatctcagcgatctgtctatttcgttcatccatagttgcctgactccccgtcgtgtagataactacgatacgggagggcttaccatctggccccagtgctgcaatgataccgcgagacccacgctcaccggctccagatttatcagcaataaaccagccagccggaagggccgagcgcagaagtggtcctgcaactttatccgcctccatccagtctattaattgttgccgggaagctagagtaagtagttcgccagttaatagtttgcgcaacgttgttgccattgctacaggcatcgtggtgtcacgctcgtcgtttggtatggcttcattcagctccggttcccaacgatcaaggcgagttacatgatcccccatgttgtgcaaaaaagcggttagctccttcggtcctccgatcgttgtcagaagtaagttggccgcagtgttatcactcatggttatggcagcactgcataattctcttactgtcatgccatccgtaagatgcttttctgtgactggtgagtactcaaccaagtcattctgagaatagtgtatgcggcgaccgagttgctcttgcccggcgtcaatacgggataataccgcgccacatagcagaactttaaaagtgctcatcattggaaaacgttcttcggggcgaaaactctcaaggatcttaccgctgttgagatccagttcgatgtaacccactcgtgcacccaactgatcttcagcatcttttactttcaccagcgtttctgggtgagcaaaaacaggaaggcaaaatgccgcaaaaaagggaataagggcgacacggaaatgttgaatactcatactcttcctttttcaatattattgaagcattt

Leu1 frag

nmt41 prom

MCS

HA tag

nmt term

Ura4 frag

beta-lactamase (AmpR)

**pINTL41HMN**

atcagggttattgtctcatgagcggatacatatttgaatgtatttagaaaaataaacaaataggggttccgcgcacatttccccgaaaagtgccacctgacgtctaaggcggccgcacattataatgcatattcgtactgtctattaaacatttttttgacattaaaaatttcgtttactaacgtagaaagcgctgataattgaggctactattttaatatctagaaacaaagattcattaattaattaagtaagctccaggatacttgtatatttcgttaaaaaagcgcaatttcaacaattcctatgaacatccatgatttcatcacaaaatcaaacatatcaggatttaacgtgaattatttgctttcttgtgattttcatctgatttctggtcatttacgttactgtaaacaagctttttaaaaaaaggattttttacgcaatattatcgatatcccaatctgtagtataaaaagtgcaacagtgttgagtttcccgaaaccaggcatttttgcggtagggtctgaaactttcacgaaaccatttcacaacaaaacttcttcaacaacatgtgtgcaaagaaaatcgtcgtcttaccaggagaccatattggccctgaaattgttgcttctgccttggaggttttgaaagtcgttgagaagaagcgacctgagttaaaactcgagtttgaagaacacaagattggaggtgcctctattgatgcctatggaacccctttgactgacgagactgtgaaggcttgtttggaagctgacggtgttcttttgggtgccgttggtggtcctgaatggaccaaccccaattgtcgtcctgagcaaggtttattgaagcttcgtaagagtatgggtgtttgggccaaccttcgaccttgcaactttgccagcaagtctttagtcaagtacagccctttgaagcctgaaatcgttgaaggtgtcgatttttgtgttgtacgagaacttactggaggttgttactttggtgagcgcactgaggacaacggatcgggttatgctatggacacttggccttacagtttggaagaagtttctcgtattgctcgtttggctgcttggttagctgaaacttccaaccctcctgctcccgtcacattactcgacaaagctaatgtctgcaggtcgatcgactctagaggatcagaaaattatcgccataaaagacagaataagtcatcagcggttgtttcatttcctatattttttttttatttttttattttttaataagggaaaatttaacgtctaaggatacagaagattgttagcacattaaagtaataaaggcttaagtagtaagtgccttagcatgttattgtatttcaaaggacataatctaaaataataacaatatcatttctcacaagttattcaattttcttttttttttctaataatatcaagaatgtattatttgtttgacataagtcaactaatttatttaatatgctggattaatcttgcagacatgtaaattaacaagttttagtcaaataacgttgaagtttcaatgaactcaaataatttctctttttttttatataaccataatctgatttatattttccgcagggatcaactgaagttatgacatttggattggatcacttataaccttggtcgccaaataatacaaaaatcagcgttataaaacaaagaaggtttttgttaagaaattaatcctctttcttgataagaaagttgaaccgaaattgcagatactgatatatgaaaataatacccacaattttgggaatagcgcaagcctcaatttaaacaataggtgaggacacatgataatgacctcaatgattgttagaagaaaagagcctcattacaaaatcgaaaaatgaatggttgggtacaagtttccaaaacatggtaaagtggactttgcgtatgagacgtaaatagaaaaaaacacttgttatatgttttctagaattattgttgtctctttatggttggatgatgcaaaatagtaatttcggttagttgctgtaaaacaccacgagacaaatagatatggatatttattaaatcaggaaaaacgtaactctcggctactggatggttcagtcacccaacgattactggggagagaaaacagggcaaaagcaaagcttaaaggaatccgattgtcattcggcaatgtgcagcgaaactaaaaaccggataatggacctgttaatcgaaacattgaagataaaggaagaggaatcctggcatatcatcaattgaataagttgaattaattatttcaatctcattctcactttctgacttatagtcgctttgttaaatcccatgggtagcagccaccatcatcaccatcatgctgaggagcaaaagttaatttctgaagaagatttgtccatggctgaagaacaaaaattgatcagcgaggaggacttacatatgtcgactctagaggatccccgggtaaaaggaatgtctcccttgccagtactgctagggtttttctttcaaactatggaagcccattcaagctgcatattacgattttgtttttcgcttttagaaagtggtttagatgagataatagaaaaattcttgatctccgacaacgagtacttttattttttttgctaatcactttactcaatattagctcgaaatcgtagaaacgtagacgggtgcgggataccgagtggtgtagttaagaatttttataaaccacgtggcccaaaaatatgaacccaaaacgtttatacatgagtatactttaagaaggctataccccttcgtgttagatgtagttttagctacccaacccgagtctatgagcttgacttcagatgtagaaggcattaaatcgttttgaatattaattaaaaaacgatgaaaattaaatatttaaaagcaatcatacgctgaaaatttagtgctgtggctaatccttcaacatggaaatgccataaaagtgactttgacaaaaaaaaaagtatatacaggtagtaaactcatctacttcattgactttgtttacagcatgtggaaggaggaatatttattgctaaatcgtagtttaacattcaataagtaatactattgaaattcgacaagattggccgcatggatgaaaaagaggcattttgctttgggagaattagtccaaattagaactgaaaaaaaaaactttacgaggcaaaaatgtcggattgagatcgtaaaagttcgctcgtcgtcttttgctttgtgattgttttcatggatacatcttgctggatatttaaattttagtactatgtataagatattctataaatgttttatcacccaaacctgttagcgccttcttaattctattcaatctggcttttgctctgagactacttcttggactttcactacttgttagttatacggaatttgtgtaattagaagtgaaataatcctttctattagtaatgcgagctcgatctacaaatcccactggctatatgtatgcatttgtgttaaaaaagtttgtatagattatttaatctactcagcattctttctctaaataggaatttgttacttaatggagaaaaaaatgtttcgatttacctagtgtatttgtttgtatactcacgtttaatttcaaacatccattctatcttgtgtaatttttggcatggtgaaaaagataatcagccttataatctttacaaaagtaagaaattctgtaaataagccttaatgcccttgctttaaattaaaatggttctttttcatgataatgtttgcactttgtgaatatattttagatagttctgtgaggtataattaagatgttttagagacttatacaattttgtctttataaattcttaattgattttaccatcccagtttaactatgcttcgtcggcatctctgcacatgtcgtgttttcttaccgtattgtcctaccaagaacctcttttttgcttggatcgaaattaaaggtttaaaagcaaagttatggatgctagagtatttcaaagctattcagctagagctgaggggatgaaaaatcccattgccaaggaattgttggctttgatggaagaaaagcaaagcaacttgtcagtcgcggtcgatttgacgaagaaatccgaaatcttagaattggtagataaaattggaccctatgtctgtgttatcaagacacatattgacgttgtcgaggatttcgaccaggatatggtagaaaaactggtggccttaggtaaaaagcatcgttttcttatctttgaggatcgcaaattcgcagacattggaaataccgtcaagctacaatatgcatctggtgtgtacaaaattgcttcttgggctcatatcacaaattgccatacagtgccaggcgagggtattatacaaggcctcaaagaagttggtttacctttgggacgtggtctcttgcttttggctgaaatgtcttccaaaggctctttggctactggttcctacacagagaaaaccttagaatggtttgagaagcataccgatttttgctttggctttatagctggtcgtcgatttcctaaccttcaaagcgactacataactatgtcccctggtatcggcttggatgttaaaggagacgggctgggacagcaatatcgtactcctgaagaagtgattgtaaactgcggtagcgatatcatcattgttggtcgtggagtctatggagctggtcgtaatcctgttgtcgaagccaagagatatagagaagctggttggaaggcatatcagcaaagactttctcagcattaaaaaaagactaatgtaaaatttttttggttggttattgaaaaagtcgatgccttgtttgcgtttgttttcctaggcgttttatgtcagaaggcatttagaattagtatacaagtactctttggtaaaattttatgtagcgactaaaatattaactattatagataaacaccttgggaataaaaagtaatttgctatagtaatttattaaacatgctcctacaacattaccacaatcttttctcttggattgacattgaataagaaaagagtgaatttttttagacttgtaatgataactatgtacaaagccaatgaaagatgtatgtagatgaatgtaaaataccatgtagacaaacaagataaaacttggttataaacattggtgttggaacagaataaattagatgtcaaaaagtttcgtcaatatcacgatttgatttgacctaatttttattgattaattgtgtcgcatatttattatcgttaaaaatgcaattgacaaaagcgtaaattttaaggctctaatgttttttcttttggtatgttatagtcaagcaaagatgtattaagttgcaaaagcatggaattgatttacgacatccgttcaactttatgaccgagttgtcgctgtgtacttactctttttttactatgaaaagtttcaaacataatatcattaattatttctttttaaacgaagtaacctgtaacataaataataggggaaaagaagaaaaaaaccataaaaattcgagaacgtaacaattattattgtaacgatgacaacccgattctttgagagagccagagacatgacttttgcattaccgtaattggtttctacttatgaattgtaaacttgtgtagggaatttcatccacagctatcgcgggctgctctctgcatcagtgttagcacattttatattaatgaagattttgctatgcaaaatacgtacatttctggcgcatatatttttatcaaacgaacatgactatcctttccactttgatggttcgcaagtattttgatttgtattaaatgcacatctaacaagccatttcgcccaaaatcaccaactggcacatgtacccaaccagttggttcctcgacttgaactgttgtaacaatttccaaatcttgaaaccctgtccgcggccgcccagctgcattaatgaatcggccaacgcgcggggagaggcggtttgcgtattgggcgctcttccgcttcctcgctcactgactcgctgcgctcggtcgttcggctgcggcgagcggtatcagctcactcaaaggcggtaatacggttatccacagaatcaggggataacgcaggaaagaacatgtgagcaaaaggccagcaaaaggccaggaaccgtaaaaaggccgcgttgctggcgtttttccataggctccgcccccctgacgagcatcacaaaaatcgacgctcaagtcagaggtggcgaaacccgacaggactataaagataccaggcgtttccccctggaagctccctcgtgcgctctcctgttccgaccctgccgcttaccggatacctgtccgcctttctcccttcgggaagcgtggcgctttctcatagctcacgctgtaggtatctcagttcggtgtaggtcgttcgctccaagctgggctgtgtgcacgaaccccccgttcagcccgaccgctgcgccttatccggtaactatcgtcttgagtccaacccggtaagacacgacttatcgccactggcagcagccactggtaacaggattagcagagcgaggtatgtaggcggtgctacagagttcttgaagtggtggcctaactacggctacactagaagaacagtatttggtatctgcgctctgctgaagccagttaccttcggaaaaagagttggtagctcttgatccggcaaacaaaccaccgctggtagcggtggtttttttgtttgcaagcagcagattacgcgcagaaaaaaaggatctcaagaagatcctttgatcttttctacggggtctgacgctcagtggaacgaaaactcacgttaagggattttggtcatgagattatcaaaaaggatcttcacctagatccttttaaattaaaaatgaagttttaaatcaatctaaagtatatatgagtaaacttggtctgacagttaccaatgcttaatcagtgaggcacctatctcagcgatctgtctatttcgttcatccatagttgcctgactccccgtcgtgtagataactacgatacgggagggcttaccatctggccccagtgctgcaatgataccgcgagacccacgctcaccggctccagatttatcagcaataaaccagccagccggaagggccgagcgcagaagtggtcctgcaactttatccgcctccatccagtctattaattgttgccgggaagctagagtaagtagttcgccagttaatagtttgcgcaacgttgttgccattgctacaggcatcgtggtgtcacgctcgtcgtttggtatggcttcattcagctccggttcccaacgatcaaggcgagttacatgatcccccatgttgtgcaaaaaagcggttagctccttcggtcctccgatcgttgtcagaagtaagttggccgcagtgttatcactcatggttatggcagcactgcataattctcttactgtcatgccatccgtaagatgcttttctgtgactggtgagtactcaaccaagtcattctgagaatagtgtatgcggcgaccgagttgctcttgcccggcgtcaatacgggataataccgcgccacatagcagaactttaaaagtgctcatcattggaaaacgttcttcggggcgaaaactctcaaggatcttaccgctgttgagatccagttcgatgtaacccactcgtgcacccaactgatcttcagcatcttttactttcaccagcgtttctgggtgagcaaaaacaggaaggcaaaatgccgcaaaaaagggaataagggcgacacggaaatgttgaatactcatactcttcctttttcaatattattgaagcattt

Leu1 frag

nmt41 prom

MCS

HM tag

nmt term

Ura4 frag

beta-lactamase (AmpR)

**pINTL41EGFPN**

atcagggttattgtctcatgagcggatacatatttgaatgtatttagaaaaataaacaaataggggttccgcgcacatttccccgaaaagtgccacctgacgtctaaggcggccgcacattataatgcatattcgtactgtctattaaacatttttttgacattaaaaatttcgtttactaacgtagaaagcgctgataattgaggctactattttaatatctagaaacaaagattcattaattaattaagtaagctccaggatacttgtatatttcgttaaaaaagcgcaatttcaacaattcctatgaacatccatgatttcatcacaaaatcaaacatatcaggatttaacgtgaattatttgctttcttgtgattttcatctgatttctggtcatttacgttactgtaaacaagctttttaaaaaaaggattttttacgcaatattatcgatatcccaatctgtagtataaaaagtgcaacagtgttgagtttcccgaaaccaggcatttttgcggtagggtctgaaactttcacgaaaccatttcacaacaaaacttcttcaacaacatgtgtgcaaagaaaatcgtcgtcttaccaggagaccatattggccctgaaattgttgcttctgccttggaggttttgaaagtcgttgagaagaagcgacctgagttaaaactcgagtttgaagaacacaagattggaggtgcctctattgatgcctatggaacccctttgactgacgagactgtgaaggcttgtttggaagctgacggtgttcttttgggtgccgttggtggtcctgaatggaccaaccccaattgtcgtcctgagcaaggtttattgaagcttcgtaagagtatgggtgtttgggccaaccttcgaccttgcaactttgccagcaagtctttagtcaagtacagccctttgaagcctgaaatcgttgaaggtgtcgatttttgtgttgtacgagaacttactggaggttgttactttggtgagcgcactgaggacaacggatcgggttatgctatggacacttggccttacagtttggaagaagtttctcgtattgctcgtttggctgcttggttagctgaaacttccaaccctcctgctcccgtcacattactcgacaaagctaatgtctgcaggtcgatcgactctagaggatcagaaaattatcgccataaaagacagaataagtcatcagcggttgtttcatttcctatattttttttttatttttttattttttaataagggaaaatttaacgtctaaggatacagaagattgttagcacattaaagtaataaaggcttaagtagtaagtgccttagcatgttattgtatttcaaaggacataatctaaaataataacaatatcatttctcacaagttattcaattttcttttttttttctaataatatcaagaatgtattatttgtttgacataagtcaactaatttatttaatatgctggattaatcttgcagacatgtaaattaacaagttttagtcaaataacgttgaagtttcaatgaactcaaataatttctctttttttttatataaccataatctgatttatattttccgcagggatcaactgaagttatgacatttggattggatcacttataaccttggtcgccaaataatacaaaaatcagcgttataaaacaaagaaggtttttgttaagaaattaatcctctttcttgataagaaagttgaaccgaaattgcagatactgatatatgaaaataatacccacaattttgggaatagcgcaagcctcaatttaaacaataggtgaggacacatgataatgacctcaatgattgttagaagaaaagagcctcattacaaaatcgaaaaatgaatggttgggtacaagtttccaaaacatggtaaagtggactttgcgtatgagacgtaaatagaaaaaaacacttgttatatgttttctagaattattgttgtctctttatggttggatgatgcaaaatagtaatttcggttagttgctgtaaaacaccacgagacaaatagatatggatatttattaaatcaggaaaaacgtaactctcggctactggatggttcagtcacccaacgattactggggagagaaaacagggcaaaagcaaagcttaaaggaatccgattgtcattcggcaatgtgcagcgaaactaaaaaccggataatggacctgttaatcgaaacattgaagataaaggaagaggaatcctggcatatcatcaattgaataagttgaattaattatttcaatctcattctcactttctgacttatagtcgctttgttaaatcatgagtaaaggagaagaacttttcactggagttgtcccaattcttgttgaattagatggtgatgttaatgggcacaaattttctgtcagtggagagggtgaaggtgatgcaacatacggaaaacttacccttaaatttatttgcactactggaaaactacctgttccatggccaacacttgtcactactttgacttatggtgttcaatgcttttcaagatacccagatcacatgaaacagcatgactttttcaagagtgccatgcccgaaggttatgtacaggaaagaactatatttttcaaagatgacgggaactacaagacacgtgctgaagtcaagtttgaaggtgatacccttgttaatagaatcgagttaaaaggtattgattttaaagaagatggaaacattcttggacacaaattggaatacaactataactcacacaatgtatacatcatggcagacaaacaaaagaatggaatcaaagttaacttcaaaattagacacaacattgaagatggaagcgttcaactagcagaccattatcaacaaaatactccaattggcgatggccctgtccttttaccagacaaccattacctgtccacacaatctgccctttcgaaagatcccaacgaaaagagagaccacatggtccttcttgagtttgtaacagctgctgggattacacatggcatggatgaactatacaaacatatgtcgactctagaggatccccgggtaaaaggaatgtctcccttgccagtactgctagggtttttctttcaaactatggaagcccattcaagctgcatattacgattttgtttttcgcttttagaaagtggtttagatgagataatagaaaaattcttgatctccgacaacgagtacttttattttttttgctaatcactttactcaatattagctcgaaatcgtagaaacgtagacgggtgcgggataccgagtggtgtagttaagaatttttataaaccacgtggcccaaaaatatgaacccaaaacgtttatacatgagtatactttaagaaggctataccccttcgtgttagatgtagttttagctacccaacccgagtctatgagcttgacttcagatgtagaaggcattaaatcgttttgaatattaattaaaaaacgatgaaaattaaatatttaaaagcaatcatacgctgaaaatttagtgctgtggctaatccttcaacatggaaatgccataaaagtgactttgacaaaaaaaaaagtatatacaggtagtaaactcatctacttcattgactttgtttacagcatgtggaaggaggaatatttattgctaaatcgtagtttaacattcaataagtaatactattgaaattcgacaagattggccgcatggatgaaaaagaggcattttgctttgggagaattagtccaaattagaactgaaaaaaaaaactttacgaggcaaaaatgtcggattgagatcgtaaaagttcgctcgtcgtcttttgctttgtgattgttttcatggatacatcttgctggatatttaaattttagtactatgtataagatattctataaatgttttatcacccaaacctgttagcgccttcttaattctattcaatctggcttttgctctgagactacttcttggactttcactacttgttagttatacggaatttgtgtaattagaagtgaaataatcctttctattagtaatgcgagctcgatctacaaatcccactggctatatgtatgcatttgtgttaaaaaagtttgtatagattatttaatctactcagcattctttctctaaataggaatttgttacttaatggagaaaaaaatgtttcgatttacctagtgtatttgtttgtatactcacgtttaatttcaaacatccattctatcttgtgtaatttttggcatggtgaaaaagataatcagccttataatctttacaaaagtaagaaattctgtaaataagccttaatgcccttgctttaaattaaaatggttctttttcatgataatgtttgcactttgtgaatatattttagatagttctgtgaggtataattaagatgttttagagacttatacaattttgtctttataaattcttaattgattttaccatcccagtttaactatgcttcgtcggcatctctgcacatgtcgtgttttcttaccgtattgtcctaccaagaacctcttttttgcttggatcgaaattaaaggtttaaaagcaaagttatggatgctagagtatttcaaagctattcagctagagctgaggggatgaaaaatcccattgccaaggaattgttggctttgatggaagaaaagcaaagcaacttgtcagtcgcggtcgatttgacgaagaaatccgaaatcttagaattggtagataaaattggaccctatgtctgtgttatcaagacacatattgacgttgtcgaggatttcgaccaggatatggtagaaaaactggtggccttaggtaaaaagcatcgttttcttatctttgaggatcgcaaattcgcagacattggaaataccgtcaagctacaatatgcatctggtgtgtacaaaattgcttcttgggctcatatcacaaattgccatacagtgccaggcgagggtattatacaaggcctcaaagaagttggtttacctttgggacgtggtctcttgcttttggctgaaatgtcttccaaaggctctttggctactggttcctacacagagaaaaccttagaatggtttgagaagcataccgatttttgctttggctttatagctggtcgtcgatttcctaaccttcaaagcgactacataactatgtcccctggtatcggcttggatgttaaaggagacgggctgggacagcaatatcgtactcctgaagaagtgattgtaaactgcggtagcgatatcatcattgttggtcgtggagtctatggagctggtcgtaatcctgttgtcgaagccaagagatatagagaagctggttggaaggcatatcagcaaagactttctcagcattaaaaaaagactaatgtaaaatttttttggttggttattgaaaaagtcgatgccttgtttgcgtttgttttcctaggcgttttatgtcagaaggcatttagaattagtatacaagtactctttggtaaaattttatgtagcgactaaaatattaactattatagataaacaccttgggaataaaaagtaatttgctatagtaatttattaaacatgctcctacaacattaccacaatcttttctcttggattgacattgaataagaaaagagtgaatttttttagacttgtaatgataactatgtacaaagccaatgaaagatgtatgtagatgaatgtaaaataccatgtagacaaacaagataaaacttggttataaacattggtgttggaacagaataaattagatgtcaaaaagtttcgtcaatatcacgatttgatttgacctaatttttattgattaattgtgtcgcatatttattatcgttaaaaatgcaattgacaaaagcgtaaattttaaggctctaatgttttttcttttggtatgttatagtcaagcaaagatgtattaagttgcaaaagcatggaattgatttacgacatccgttcaactttatgaccgagttgtcgctgtgtacttactctttttttactatgaaaagtttcaaacataatatcattaattatttctttttaaacgaagtaacctgtaacataaataataggggaaaagaagaaaaaaaccataaaaattcgagaacgtaacaattattattgtaacgatgacaacccgattctttgagagagccagagacatgacttttgcattaccgtaattggtttctacttatgaattgtaaacttgtgtagggaatttcatccacagctatcgcgggctgctctctgcatcagtgttagcacattttatattaatgaagattttgctatgcaaaatacgtacatttctggcgcatatatttttatcaaacgaacatgactatcctttccactttgatggttcgcaagtattttgatttgtattaaatgcacatctaacaagccatttcgcccaaaatcaccaactggcacatgtacccaaccagttggttcctcgacttgaactgttgtaacaatttccaaatcttgaaaccctgtccgcggccgcccagctgcattaatgaatcggccaacgcgcggggagaggcggtttgcgtattgggcgctcttccgcttcctcgctcactgactcgctgcgctcggtcgttcggctgcggcgagcggtatcagctcactcaaaggcggtaatacggttatccacagaatcaggggataacgcaggaaagaacatgtgagcaaaaggccagcaaaaggccaggaaccgtaaaaaggccgcgttgctggcgtttttccataggctccgcccccctgacgagcatcacaaaaatcgacgctcaagtcagaggtggcgaaacccgacaggactataaagataccaggcgtttccccctggaagctccctcgtgcgctctcctgttccgaccctgccgcttaccggatacctgtccgcctttctcccttcgggaagcgtggcgctttctcatagctcacgctgtaggtatctcagttcggtgtaggtcgttcgctccaagctgggctgtgtgcacgaaccccccgttcagcccgaccgctgcgccttatccggtaactatcgtcttgagtccaacccggtaagacacgacttatcgccactggcagcagccactggtaacaggattagcagagcgaggtatgtaggcggtgctacagagttcttgaagtggtggcctaactacggctacactagaagaacagtatttggtatctgcgctctgctgaagccagttaccttcggaaaaagagttggtagctcttgatccggcaaacaaaccaccgctggtagcggtggtttttttgtttgcaagcagcagattacgcgcagaaaaaaaggatctcaagaagatcctttgatcttttctacggggtctgacgctcagtggaacgaaaactcacgttaagggattttggtcatgagattatcaaaaaggatcttcacctagatccttttaaattaaaaatgaagttttaaatcaatctaaagtatatatgagtaaacttggtctgacagttaccaatgcttaatcagtgaggcacctatctcagcgatctgtctatttcgttcatccatagttgcctgactccccgtcgtgtagataactacgatacgggagggcttaccatctggccccagtgctgcaatgataccgcgagacccacgctcaccggctccagatttatcagcaataaaccagccagccggaagggccgagcgcagaagtggtcctgcaactttatccgcctccatccagtctattaattgttgccgggaagctagagtaagtagttcgccagttaatagtttgcgcaacgttgttgccattgctacaggcatcgtggtgtcacgctcgtcgtttggtatggcttcattcagctccggttcccaacgatcaaggcgagttacatgatcccccatgttgtgcaaaaaagcggttagctccttcggtcctccgatcgttgtcagaagtaagttggccgcagtgttatcactcatggttatggcagcactgcataattctcttactgtcatgccatccgtaagatgcttttctgtgactggtgagtactcaaccaagtcattctgagaatagtgtatgcggcgaccgagttgctcttgcccggcgtcaatacgggataataccgcgccacatagcagaactttaaaagtgctcatcattggaaaacgttcttcggggcgaaaactctcaaggatcttaccgctgttgagatccagttcgatgtaacccactcgtgcacccaactgatcttcagcatcttttactttcaccagcgtttctgggtgagcaaaaacaggaaggcaaaatgccgcaaaaaagggaataagggcgacacggaaatgttgaatactcatactcttcctttttcaatattattgaagcattt

Leu1 frag

nmt41 prom

MCS

eGFP tag

nmt term

Ura4 frag

beta-lactamase (AmpR)

**pINTL41EGFPC**

atcagggttattgtctcatgagcggatacatatttgaatgtatttagaaaaataaacaaataggggttccgcgcacatttccccgaaaagtgccacctgacgtctaaggcggccgcacattataatgcatattcgtactgtctattaaacatttttttgacattaaaaatttcgtttactaacgtagaaagcgctgataattgaggctactattttaatatctagaaacaaagattcattaattaattaagtaagctccaggatacttgtatatttcgttaaaaaagcgcaatttcaacaattcctatgaacatccatgatttcatcacaaaatcaaacatatcaggatttaacgtgaattatttgctttcttgtgattttcatctgatttctggtcatttacgttactgtaaacaagctttttaaaaaaaggattttttacgcaatattatcgatatcccaatctgtagtataaaaagtgcaacagtgttgagtttcccgaaaccaggcatttttgcggtagggtctgaaactttcacgaaaccatttcacaacaaaacttcttcaacaacatgtgtgcaaagaaaatcgtcgtcttaccaggagaccatattggccctgaaattgttgcttctgccttggaggttttgaaagtcgttgagaagaagcgacctgagttaaaactcgagtttgaagaacacaagattggaggtgcctctattgatgcctatggaacccctttgactgacgagactgtgaaggcttgtttggaagctgacggtgttcttttgggtgccgttggtggtcctgaatggaccaaccccaattgtcgtcctgagcaaggtttattgaagcttcgtaagagtatgggtgtttgggccaaccttcgaccttgcaactttgccagcaagtctttagtcaagtacagccctttgaagcctgaaatcgttgaaggtgtcgatttttgtgttgtacgagaacttactggaggttgttactttggtgagcgcactgaggacaacggatcgggttatgctatggacacttggccttacagtttggaagaagtttctcgtattgctcgtttggctgcttggttagctgaaacttccaaccctcctgctcccgtcacattactcgacaaagctaatgtctgcaggtcgatcgactctagaggatcagaaaattatcgccataaaagacagaataagtcatcagcggttgtttcatttcctatattttttttttatttttttattttttaataagggaaaatttaacgtctaaggatacagaagattgttagcacattaaagtaataaaggcttaagtagtaagtgccttagcatgttattgtatttcaaaggacataatctaaaataataacaatatcatttctcacaagttattcaattttcttttttttttctaataatatcaagaatgtattatttgtttgacataagtcaactaatttatttaatatgctggattaatcttgcagacatgtaaattaacaagttttagtcaaataacgttgaagtttcaatgaactcaaataatttctctttttttttatataaccataatctgatttatattttccgcagggatcaactgaagttatgacatttggattggatcacttataaccttggtcgccaaataatacaaaaatcagcgttataaaacaaagaaggtttttgttaagaaattaatcctctttcttgataagaaagttgaaccgaaattgcagatactgatatatgaaaataatacccacaattttgggaatagcgcaagcctcaatttaaacaataggtgaggacacatgataatgacctcaatgattgttagaagaaaagagcctcattacaaaatcgaaaaatgaatggttgggtacaagtttccaaaacatggtaaagtggactttgcgtatgagacgtaaatagaaaaaaacacttgttatatgttttctagaattattgttgtctctttatggttggatgatgcaaaatagtaatttcggttagttgctgtaaaacaccacgagacaaatagatatggatatttattaaatcaggaaaaacgtaactctcggctactggatggttcagtcacccaacgattactggggagagaaaacagggcaaaagcaaagcttaaaggaatccgattgtcattcggcaatgtgcagcgaaactaaaaaccggataatggacctgttaatcgaaacattgaagataaaggaagaggaatcctggcatatcatcaattgaataagttgaattaattatttcaatctcattctcactttctgacttatagtcgctttgttaaatcatatgtcgacatggtaccagatctctcgaggatccccgggtatgagtaaaggagaagaacttttcactggagttgtcccaattcttgttgaattagatggtgatgttaatgggcacaaattttctgtcagtggagagggtgaaggtgatgcaacatacggaaaacttacccttaaatttatttgcactactggaaaactacctgttccatggccaacacttgtcactactttgacttatggtgttcaatgcttttcaagatacccagatcacatgaaacagcatgactttttcaagagtgccatgcccgaaggttatgtacaggaaagaactatatttttcaaagatgacgggaactacaagacacgtgctgaagtcaagtttgaaggtgatacccttgttaatagaatcgagttaaaaggtattgattttaaagaagatggaaacattcttggacacaaattggaatacaactataactcacacaatgtatacatcatggcagacaaacaaaagaatggaatcaaagttaacttcaaaattagacacaacattgaagatggaagcgttcaactagcagaccattatcaacaaaatactccaattggcgatggccctgtccttttaccagacaaccattacctgtccacacaatctgccctttcgaaagatcccaacgaaaagagagaccacatggtccttcttgagtttgtaacagctgctgggattacacatggcatggatgaactatacaaacatacgtaaccatggggtaaaaggaatgtctcccttgccagtactgctagggtttttctttcaaactatggaagcccattcaagctgcatattacgattttgtttttcgcttttagaaagtggtttagatgagataatagaaaaattcttgatctccgacaacgagtacttttattttttttgctaatcactttactcaatattagctcgaaatcgtagaaacgtagacgggtgcgggataccgagtggtgtagttaagaatttttataaaccacgtggcccaaaaatatgaacccaaaacgtttatacatgagtatactttaagaaggctataccccttcgtgttagatgtagttttagctacccaacccgagtctatgagcttgacttcagatgtagaaggcattaaatcgttttgaatattaattaaaaaacgatgaaaattaaatatttaaaagcaatcatacgctgaaaatttagtgctgtggctaatccttcaacatggaaatgccataaaagtgactttgacaaaaaaaaaagtatatacaggtagtaaactcatctacttcattgactttgtttacagcatgtggaaggaggaatatttattgctaaatcgtagtttaacattcaataagtaatactattgaaattcgacaagattggccgcatggatgaaaaagaggcattttgctttgggagaattagtccaaattagaactgaaaaaaaaaactttacgaggcaaaaatgtcggattgagatcgtaaaagttcgctcgtcgtcttttgctttgtgattgttttcatggatacatcttgctggatatttaaattttagtactatgtataagatattctataaatgttttatcacccaaacctgttagcgccttcttaattctattcaatctggcttttgctctgagactacttcttggactttcactacttgttagttatacggaatttgtgtaattagaagtgaaataatcctttctattagtaatgcgagctcgatctacaaatcccactggctatatgtatgcatttgtgttaaaaaagtttgtatagattatttaatctactcagcattctttctctaaataggaatttgttacttaatggagaaaaaaatgtttcgatttacctagtgtatttgtttgtatactcacgtttaatttcaaacatccattctatcttgtgtaatttttggcatggtgaaaaagataatcagccttataatctttacaaaagtaagaaattctgtaaataagccttaatgcccttgctttaaattaaaatggttctttttcatgataatgtttgcactttgtgaatatattttagatagttctgtgaggtataattaagatgttttagagacttatacaattttgtctttataaattcttaattgattttaccatcccagtttaactatgcttcgtcggcatctctgcacatgtcgtgttttcttaccgtattgtcctaccaagaacctcttttttgcttggatcgaaattaaaggtttaaaagcaaagttatggatgctagagtatttcaaagctattcagctagagctgaggggatgaaaaatcccattgccaaggaattgttggctttgatggaagaaaagcaaagcaacttgtcagtcgcggtcgatttgacgaagaaatccgaaatcttagaattggtagataaaattggaccctatgtctgtgttatcaagacacatattgacgttgtcgaggatttcgaccaggatatggtagaaaaactggtggccttaggtaaaaagcatcgttttcttatctttgaggatcgcaaattcgcagacattggaaataccgtcaagctacaatatgcatctggtgtgtacaaaattgcttcttgggctcatatcacaaattgccatacagtgccaggcgagggtattatacaaggcctcaaagaagttggtttacctttgggacgtggtctcttgcttttggctgaaatgtcttccaaaggctctttggctactggttcctacacagagaaaaccttagaatggtttgagaagcataccgatttttgctttggctttatagctggtcgtcgatttcctaaccttcaaagcgactacataactatgtcccctggtatcggcttggatgttaaaggagacgggctgggacagcaatatcgtactcctgaagaagtgattgtaaactgcggtagcgatatcatcattgttggtcgtggagtctatggagctggtcgtaatcctgttgtcgaagccaagagatatagagaagctggttggaaggcatatcagcaaagactttctcagcattaaaaaaagactaatgtaaaatttttttggttggttattgaaaaagtcgatgccttgtttgcgtttgttttcctaggcgttttatgtcagaaggcatttagaattagtatacaagtactctttggtaaaattttatgtagcgactaaaatattaactattatagataaacaccttgggaataaaaagtaatttgctatagtaatttattaaacatgctcctacaacattaccacaatcttttctcttggattgacattgaataagaaaagagtgaatttttttagacttgtaatgataactatgtacaaagccaatgaaagatgtatgtagatgaatgtaaaataccatgtagacaaacaagataaaacttggttataaacattggtgttggaacagaataaattagatgtcaaaaagtttcgtcaatatcacgatttgatttgacctaatttttattgattaattgtgtcgcatatttattatcgttaaaaatgcaattgacaaaagcgtaaattttaaggctctaatgttttttcttttggtatgttatagtcaagcaaagatgtattaagttgcaaaagcatggaattgatttacgacatccgttcaactttatgaccgagttgtcgctgtgtacttactctttttttactatgaaaagtttcaaacataatatcattaattatttctttttaaacgaagtaacctgtaacataaataataggggaaaagaagaaaaaaaccataaaaattcgagaacgtaacaattattattgtaacgatgacaacccgattctttgagagagccagagacatgacttttgcattaccgtaattggtttctacttatgaattgtaaacttgtgtagggaatttcatccacagctatcgcgggctgctctctgcatcagtgttagcacattttatattaatgaagattttgctatgcaaaatacgtacatttctggcgcatatatttttatcaaacgaacatgactatcctttccactttgatggttcgcaagtattttgatttgtattaaatgcacatctaacaagccatttcgcccaaaatcaccaactggcacatgtacccaaccagttggttcctcgacttgaactgttgtaacaatttccaaatcttgaaaccctgtccgcggccgcccagctgcattaatgaatcggccaacgcgcggggagaggcggtttgcgtattgggcgctcttccgcttcctcgctcactgactcgctgcgctcggtcgttcggctgcggcgagcggtatcagctcactcaaaggcggtaatacggttatccacagaatcaggggataacgcaggaaagaacatgtgagcaaaaggccagcaaaaggccaggaaccgtaaaaaggccgcgttgctggcgtttttccataggctccgcccccctgacgagcatcacaaaaatcgacgctcaagtcagaggtggcgaaacccgacaggactataaagataccaggcgtttccccctggaagctccctcgtgcgctctcctgttccgaccctgccgcttaccggatacctgtccgcctttctcccttcgggaagcgtggcgctttctcatagctcacgctgtaggtatctcagttcggtgtaggtcgttcgctccaagctgggctgtgtgcacgaaccccccgttcagcccgaccgctgcgccttatccggtaactatcgtcttgagtccaacccggtaagacacgacttatcgccactggcagcagccactggtaacaggattagcagagcgaggtatgtaggcggtgctacagagttcttgaagtggtggcctaactacggctacactagaagaacagtatttggtatctgcgctctgctgaagccagttaccttcggaaaaagagttggtagctcttgatccggcaaacaaaccaccgctggtagcggtggtttttttgtttgcaagcagcagattacgcgcagaaaaaaaggatctcaagaagatcctttgatcttttctacggggtctgacgctcagtggaacgaaaactcacgttaagggattttggtcatgagattatcaaaaaggatcttcacctagatccttttaaattaaaaatgaagttttaaatcaatctaaagtatatatgagtaaacttggtctgacagttaccaatgcttaatcagtgaggcacctatctcagcgatctgtctatttcgttcatccatagttgcctgactccccgtcgtgtagataactacgatacgggagggcttaccatctggccccagtgctgcaatgataccgcgagacccacgctcaccggctccagatttatcagcaataaaccagccagccggaagggccgagcgcagaagtggtcctgcaactttatccgcctccatccagtctattaattgttgccgggaagctagagtaagtagttcgccagttaatagtttgcgcaacgttgttgccattgctacaggcatcgtggtgtcacgctcgtcgtttggtatggcttcattcagctccggttcccaacgatcaaggcgagttacatgatcccccatgttgtgcaaaaaagcggttagctccttcggtcctccgatcgttgtcagaagtaagttggccgcagtgttatcactcatggttatggcagcactgcataattctcttactgtcatgccatccgtaagatgcttttctgtgactggtgagtactcaaccaagtcattctgagaatagtgtatgcggcgaccgagttgctcttgcccggcgtcaatacgggataataccgcgccacatagcagaactttaaaagtgctcatcattggaaaacgttcttcggggcgaaaactctcaaggatcttaccgctgttgagatccagttcgatgtaacccactcgtgcacccaactgatcttcagcatcttttactttcaccagcgtttctgggtgagcaaaaacaggaaggcaaaatgccgcaaaaaagggaataagggcgacacggaaatgttgaatactcatactcttcctttttcaatattattgaagcattt

Leu1 frag

nmt41 prom

MCS

eGFP tag

nmt term

Ura4 frag

beta-lactamase (AmpR)

**pINTL41GST**

atcagggttattgtctcatgagcggatacatatttgaatgtatttagaaaaataaacaaataggggttccgcgcacatttccccgaaaagtgccacctgacgtctaaggcggccgcacattataatgcatattcgtactgtctattaaacatttttttgacattaaaaatttcgtttactaacgtagaaagcgctgataattgaggctactattttaatatctagaaacaaagattcattaattaattaagtaagctccaggatacttgtatatttcgttaaaaaagcgcaatttcaacaattcctatgaacatccatgatttcatcacaaaatcaaacatatcaggatttaacgtgaattatttgctttcttgtgattttcatctgatttctggtcatttacgttactgtaaacaagctttttaaaaaaaggattttttacgcaatattatcgatatcccaatctgtagtataaaaagtgcaacagtgttgagtttcccgaaaccaggcatttttgcggtagggtctgaaactttcacgaaaccatttcacaacaaaacttcttcaacaacatgtgtgcaaagaaaatcgtcgtcttaccaggagaccatattggccctgaaattgttgcttctgccttggaggttttgaaagtcgttgagaagaagcgacctgagttaaaactcgagtttgaagaacacaagattggaggtgcctctattgatgcctatggaacccctttgactgacgagactgtgaaggcttgtttggaagctgacggtgttcttttgggtgccgttggtggtcctgaatggaccaaccccaattgtcgtcctgagcaaggtttattgaagcttcgtaagagtatgggtgtttgggccaaccttcgaccttgcaactttgccagcaagtctttagtcaagtacagccctttgaagcctgaaatcgttgaaggtgtcgatttttgtgttgtacgagaacttactggaggttgttactttggtgagcgcactgaggacaacggatcgggttatgctatggacacttggccttacagtttggaagaagtttctcgtattgctcgtttggctgcttggttagctgaaacttccaaccctcctgctcccgtcacattactcgacaaagctaatgtctgcaggtcgatcgactctagaggatcagaaaattatcgccataaaagacagaataagtcatcagcggttgtttcatttcctatattttttttttatttttttattttttaataagggaaaatttaacgtctaaggatacagaagattgttagcacattaaagtaataaaggcttaagtagtaagtgccttagcatgttattgtatttcaaaggacataatctaaaataataacaatatcatttctcacaagttattcaattttcttttttttttctaataatatcaagaatgtattatttgtttgacataagtcaactaatttatttaatatgctggattaatcttgcagacatgtaaattaacaagttttagtcaaataacgttgaagtttcaatgaactcaaataatttctctttttttttatataaccataatctgatttatattttccgcagggatcaactgaagttatgacatttggattggatcacttataaccttggtcgccaaataatacaaaaatcagcgttataaaacaaagaaggtttttgttaagaaattaatcctctttcttgataagaaagttgaaccgaaattgcagatactgatatatgaaaataatacccacaattttgggaatagcgcaagcctcaatttaaacaataggtgaggacacatgataatgacctcaatgattgttagaagaaaagagcctcattacaaaatcgaaaaatgaatggttgggtacaagtttccaaaacatggtaaagtggactttgcgtatgagacgtaaatagaaaaaaacacttgttatatgttttctagaattattgttgtctctttatggttggatgatgcaaaatagtaatttcggttagttgctgtaaaacaccacgagacaaatagatatggatatttattaaatcaggaaaaacgtaactctcggctactggatggttcagtcacccaacgattactggggagagaaaacagggcaaaagcaaagcttaaaggaatccgattgtcattcggcaatgtgcagcgaaactaaaaaccggataatggacctgttaatcgaaacattgaagataaaggaagaggaatcctggcatatcatcaattgaataagttgaattaattatttcaatctcattctcactttctgacttatagtcgctttgttaaatcatatgtcccctatactaggttattggaaaattaagggccttgtgcaacccactcgacttcttttggaatatcttgaagaaaaatatgaagagcatttgtatgagcgcgatgaaggtgataaatggcgaaacaaaaagtttgaattgggtttggagtttcccaatcttccttattatattgatggtgatgttaaattaacacagtctatggccatcatacgttatatagctgacaagcacaacatgttgggtggttgtccaaaagagcgtgcagagatttcaatgcttgaaggagcggttttggatattagatacggtgtttcgagaattgcatatagtaaagactttgaaactctcaaagttgattttcttagcaagctacctgaaatgctgaaaatgttcgaagatcgtttatgtcataaaacatatttaaatggtgatcatgtaacccatcctgacttcatgttgtatgacgctcttgatgttgttttatacatggacccaatgtgcctggatgcgttcccaaaattagtttgttttaaaaaacgtattgaagctatcccacaaattgataagtacttgaaatccagcaagtatatagcatggcctttgcagggctggcaagccacgtttggtggtggcgaccatcctccaaaatcggatctggttccgcgtggatccccgggtaaaaggaatgtctcccttgccagtactgctagggtttttctttcaaactatggaagcccattcaagctgcatattacgattttgtttttcgcttttagaaagtggtttagatgagataatagaaaaattcttgatctccgacaacgagtacttttattttttttgctaatcactttactcaatattagctcgaaatcgtagaaacgtagacgggtgcgggataccgagtggtgtagttaagaatttttataaaccacgtggcccaaaaatatgaacccaaaacgtttatacatgagtatactttaagaaggctataccccttcgtgttagatgtagttttagctacccaacccgagtctatgagcttgacttcagatgtagaaggcattaaatcgttttgaatattaattaaaaaacgatgaaaattaaatatttaaaagcaatcatacgctgaaaatttagtgctgtggctaatccttcaacatggaaatgccataaaagtgactttgacaaaaaaaaaagtatatacaggtagtaaactcatctacttcattgactttgtttacagcatgtggaaggaggaatatttattgctaaatcgtagtttaacattcaataagtaatactattgaaattcgacaagattggccgcatggatgaaaaagaggcattttgctttgggagaattagtccaaattagaactgaaaaaaaaaactttacgaggcaaaaatgtcggattgagatcgtaaaagttcgctcgtcgtcttttgctttgtgattgttttcatggatacatcttgctggatatttaaattttagtactatgtataagatattctataaatgttttatcacccaaacctgttagcgccttcttaattctattcaatctggcttttgctctgagactacttcttggactttcactacttgttagttatacggaatttgtgtaattagaagtgaaataatcctttctattagtaatgcgagctcgatctacaaatcccactggctatatgtatgcatttgtgttaaaaaagtttgtatagattatttaatctactcagcattctttctctaaataggaatttgttacttaatggagaaaaaaatgtttcgatttacctagtgtatttgtttgtatactcacgtttaatttcaaacatccattctatcttgtgtaatttttggcatggtgaaaaagataatcagccttataatctttacaaaagtaagaaattctgtaaataagccttaatgcccttgctttaaattaaaatggttctttttcatgataatgtttgcactttgtgaatatattttagatagttctgtgaggtataattaagatgttttagagacttatacaattttgtctttataaattcttaattgattttaccatcccagtttaactatgcttcgtcggcatctctgcacatgtcgtgttttcttaccgtattgtcctaccaagaacctcttttttgcttggatcgaaattaaaggtttaaaagcaaagttatggatgctagagtatttcaaagctattcagctagagctgaggggatgaaaaatcccattgccaaggaattgttggctttgatggaagaaaagcaaagcaacttgtcagtcgcggtcgatttgacgaagaaatccgaaatcttagaattggtagataaaattggaccctatgtctgtgttatcaagacacatattgacgttgtcgaggatttcgaccaggatatggtagaaaaactggtggccttaggtaaaaagcatcgttttcttatctttgaggatcgcaaattcgcagacattggaaataccgtcaagctacaatatgcatctggtgtgtacaaaattgcttcttgggctcatatcacaaattgccatacagtgccaggcgagggtattatacaaggcctcaaagaagttggtttacctttgggacgtggtctcttgcttttggctgaaatgtcttccaaaggctctttggctactggttcctacacagagaaaaccttagaatggtttgagaagcataccgatttttgctttggctttatagctggtcgtcgatttcctaaccttcaaagcgactacataactatgtcccctggtatcggcttggatgttaaaggagacgggctgggacagcaatatcgtactcctgaagaagtgattgtaaactgcggtagcgatatcatcattgttggtcgtggagtctatggagctggtcgtaatcctgttgtcgaagccaagagatatagagaagctggttggaaggcatatcagcaaagactttctcagcattaaaaaaagactaatgtaaaatttttttggttggttattgaaaaagtcgatgccttgtttgcgtttgttttcctaggcgttttatgtcagaaggcatttagaattagtatacaagtactctttggtaaaattttatgtagcgactaaaatattaactattatagataaacaccttgggaataaaaagtaatttgctatagtaatttattaaacatgctcctacaacattaccacaatcttttctcttggattgacattgaataagaaaagagtgaatttttttagacttgtaatgataactatgtacaaagccaatgaaagatgtatgtagatgaatgtaaaataccatgtagacaaacaagataaaacttggttataaacattggtgttggaacagaataaattagatgtcaaaaagtttcgtcaatatcacgatttgatttgacctaatttttattgattaattgtgtcgcatatttattatcgttaaaaatgcaattgacaaaagcgtaaattttaaggctctaatgttttttcttttggtatgttatagtcaagcaaagatgtattaagttgcaaaagcatggaattgatttacgacatccgttcaactttatgaccgagttgtcgctgtgtacttactctttttttactatgaaaagtttcaaacataatatcattaattatttctttttaaacgaagtaacctgtaacataaataataggggaaaagaagaaaaaaaccataaaaattcgagaacgtaacaattattattgtaacgatgacaacccgattctttgagagagccagagacatgacttttgcattaccgtaattggtttctacttatgaattgtaaacttgtgtagggaatttcatccacagctatcgcgggctgctctctgcatcagtgttagcacattttatattaatgaagattttgctatgcaaaatacgtacatttctggcgcatatatttttatcaaacgaacatgactatcctttccactttgatggttcgcaagtattttgatttgtattaaatgcacatctaacaagccatttcgcccaaaatcaccaactggcacatgtacccaaccagttggttcctcgacttgaactgttgtaacaatttccaaatcttgaaaccctgtccgcggccgcccagctgcattaatgaatcggccaacgcgcggggagaggcggtttgcgtattgggcgctcttccgcttcctcgctcactgactcgctgcgctcggtcgttcggctgcggcgagcggtatcagctcactcaaaggcggtaatacggttatccacagaatcaggggataacgcaggaaagaacatgtgagcaaaaggccagcaaaaggccaggaaccgtaaaaaggccgcgttgctggcgtttttccataggctccgcccccctgacgagcatcacaaaaatcgacgctcaagtcagaggtggcgaaacccgacaggactataaagataccaggcgtttccccctggaagctccctcgtgcgctctcctgttccgaccctgccgcttaccggatacctgtccgcctttctcccttcgggaagcgtggcgctttctcatagctcacgctgtaggtatctcagttcggtgtaggtcgttcgctccaagctgggctgtgtgcacgaaccccccgttcagcccgaccgctgcgccttatccggtaactatcgtcttgagtccaacccggtaagacacgacttatcgccactggcagcagccactggtaacaggattagcagagcgaggtatgtaggcggtgctacagagttcttgaagtggtggcctaactacggctacactagaagaacagtatttggtatctgcgctctgctgaagccagttaccttcggaaaaagagttggtagctcttgatccggcaaacaaaccaccgctggtagcggtggtttttttgtttgcaagcagcagattacgcgcagaaaaaaaggatctcaagaagatcctttgatcttttctacggggtctgacgctcagtggaacgaaaactcacgttaagggattttggtcatgagattatcaaaaaggatcttcacctagatccttttaaattaaaaatgaagttttaaatcaatctaaagtatatatgagtaaacttggtctgacagttaccaatgcttaatcagtgaggcacctatctcagcgatctgtctatttcgttcatccatagttgcctgactccccgtcgtgtagataactacgatacgggagggcttaccatctggccccagtgctgcaatgataccgcgagacccacgctcaccggctccagatttatcagcaataaaccagccagccggaagggccgagcgcagaagtggtcctgcaactttatccgcctccatccagtctattaattgttgccgggaagctagagtaagtagttcgccagttaatagtttgcgcaacgttgttgccattgctacaggcatcgtggtgtcacgctcgtcgtttggtatggcttcattcagctccggttcccaacgatcaaggcgagttacatgatcccccatgttgtgcaaaaaagcggttagctccttcggtcctccgatcgttgtcagaagtaagttggccgcagtgttatcactcatggttatggcagcactgcataattctcttactgtcatgccatccgtaagatgcttttctgtgactggtgagtactcaaccaagtcattctgagaatagtgtatgcggcgaccgagttgctcttgcccggcgtcaatacgggataataccgcgccacatagcagaactttaaaagtgctcatcattggaaaacgttcttcggggcgaaaactctcaaggatcttaccgctgttgagatccagttcgatgtaacccactcgtgcacccaactgatcttcagcatcttttactttcaccagcgtttctgggtgagcaaaaacaggaaggcaaaatgccgcaaaaaagggaataagggcgacacggaaatgttgaatactcatactcttcctttttcaatattattgaagcattt

Leu1 frag

nmt41 prom

MCS

GST tag

nmt term

Ura4 frag

beta-lactamase (AmpR)

**pINTL1**

atcagggttattgtctcatgagcggatacatatttgaatgtatttagaaaaataaacaaataggggttccgcgcacatttccccgaaaagtgccacctgacgtctaaggcggccgcacattataatgcatattcgtactgtctattaaacatttttttgacattaaaaatttcgtttactaacgtagaaagcgctgataattgaggctactattttaatatctagaaacaaagattcattaattaattaagtaagctccaggatacttgtatatttcgttaaaaaagcgcaatttcaacaattcctatgaacatccatgatttcatcacaaaatcaaacatatcaggatttaacgtgaattatttgctttcttgtgattttcatctgatttctggtcatttacgttactgtaaacaagctttttaaaaaaaggattttttacgcaatattatcgatatcccaatctgtagtataaaaagtgcaacagtgttgagtttcccgaaaccaggcatttttgcggtagggtctgaaactttcacgaaaccatttcacaacaaaacttcttcaacaacatgtgtgcaaagaaaatcgtcgtcttaccaggagaccatattggccctgaaattgttgcttctgccttggaggttttgaaagtcgttgagaagaagcgacctgagttaaaactcgagtttgaagaacacaagattggaggtgcctctattgatgcctatggaacccctttgactgacgagactgtgaaggcttgtttggaagctgacggtgttcttttgggtgccgttggtggtcctgaatggaccaaccccaattgtcgtcctgagcaaggtttattgaagcttcgtaagagtatgggtgtttgggccaaccttcgaccttgcaactttgccagcaagtctttagtcaagtacagccctttgaagcctgaaatcgttgaaggtgtcgatttttgtgttgtacgagaacttactggaggttgttactttggtgagcgcactgaggacaacggatcgggttatgctatggacacttggccttacagtttggaagaagtttctcgtattgctcgtttggctgcttggttagctgaaacttccaaccctcctgctcccgtcacattactcgacaaagctaatgtctgcaggtcgatcgactctagaggatcagaaaattatcgccataaaagacagaataagtcatcagcggttgtttcatttcctatattttttttttatttttttattttttaataagggaaaatttaacgtctaaggatacagaagattgttagcacattaaagtaataaaggcttaagtagtaagtgccttagcatgttattgtatttcaaaggacataatctaaaataataacaatatcatttctcacaagttattcaattttcttttttttttctaataatatcaagaatgtattatttgtttgacataagtcaactaatttatttaatatgctggattaatcttgcagacatgtaaattaacaagttttagtcaaataacgttgaagtttcaatgaactcaaataatttctctttttttttatataaccataatctgatttatattttccgcagggatcaactgaagttatgacatttggattggatcacttataaccttggtcgccaaataatacaaaaatcagcgttataaaacaaagaaggtttttgttaagaaattaatcctctttcttgataagaaagttgaaccgaaattgcagatactgatatatgaaaataatacccacaattttgggaatagcgcaagcctcaatttaaacaataggtgaggacacatgataatgacctcaatgattgttagaagaaaagagcctcattacaaaatcgaaaaatgaatggttgggtacaagtttccaaaacatggtaaagtggactttgcgtatgagacgtaaatagaaaaaaacacttgttatatgttttctagaattattgttgtctctttatggttggatgatgcaaaatagtaatttcggttagttgctgtaaaacaccacgagacaaatagatatggatatttattaaatcaggaaaaacgtaactctcggctactggatggttcagtcacccaacgattactggggagagaaaacagggcaaaagcaaagcttaaaggaatccgattgtcattcggcaatgtgcagcgaaactaaaaaccggataatggacctgttaatcgaaacattgaagatatataaaggaagaggaatcctggcatatcatcaattgaataagttgaattaattatttcaatctcattctcactttctgacttatagtcgctttgttaaatcatatgtcgactctagaggatccccgggtaaaaggaatgtctcccttgccagtactgctagggtttttctttcaaactatggaagcccattcaagctgcatattacgattttgtttttcgcttttagaaagtggtttagatgagataatagaaaaattcttgatctccgacaacgagtacttttattttttttgctaatcactttactcaatattagctcgaaatcgtagaaacgtagacgggtgcgggataccgagtggtgtagttaagaatttttataaaccacgtggcccaaaaatatgaacccaaaacgtttatacatgagtatactttaagaaggctataccccttcgtgttagatgtagttttagctacccaacccgagtctatgagcttgacttcagatgtagaaggcattaaatcgttttgaatattaattaaaaaacgatgaaaattaaatatttaaaagcaatcatacgctgaaaatttagtgctgtggctaatccttcaacatggaaatgccataaaagtgactttgacaaaaaaaaaagtatatacaggtagtaaactcatctacttcattgactttgtttacagcatgtggaaggaggaatatttattgctaaatcgtagtttaacattcaataagtaatactattgaaattcgacaagattggccgcatggatgaaaaagaggcattttgctttgggagaattagtccaaattagaactgaaaaaaaaaactttacgaggcaaaaatgtcggattgagatcgtaaaagttcgctcgtcgtcttttgctttgtgattgttttcatggatacatcttgctggatatttaaattttagtactatgtataagatattctataaatgttttatcacccaaacctgttagcgccttcttaattctattcaatctggcttttgctctgagactacttcttggactttcactacttgttagttatacggaatttgtgtaattagaagtgaaataatcctttctattagtaatgcgagctcgatctacaaatcccactggctatatgtatgcatttgtgttaaaaaagtttgtatagattatttaatctactcagcattctttctctaaataggaatttgttacttaatggagaaaaaaatgtttcgatttacctagtgtatttgtttgtatactcacgtttaatttcaaacatccattctatcttgtgtaatttttggcatggtgaaaaagataatcagccttataatctttacaaaagtaagaaattctgtaaataagccttaatgcccttgctttaaattaaaatggttctttttcatgataatgtttgcactttgtgaatatattttagatagttctgtgaggtataattaagatgttttagagacttatacaattttgtctttataaattcttaattgattttaccatcccagtttaactatgcttcgtcggcatctctgcacatgtcgtgttttcttaccgtattgtcctaccaagaacctcttttttgcttggatcgaaattaaaggtttaaaagcaaagttatggatgctagagtatttcaaagctattcagctagagctgaggggatgaaaaatcccattgccaaggaattgttggctttgatggaagaaaagcaaagcaacttgtcagtcgcggtcgatttgacgaagaaatccgaaatcttagaattggtagataaaattggaccctatgtctgtgttatcaagacacatattgacgttgtcgaggatttcgaccaggatatggtagaaaaactggtggccttaggtaaaaagcatcgttttcttatctttgaggatcgcaaattcgcagacattggaaataccgtcaagctacaatatgcatctggtgtgtacaaaattgcttcttgggctcatatcacaaattgccatacagtgccaggcgagggtattatacaaggcctcaaagaagttggtttacctttgggacgtggtctcttgcttttggctgaaatgtcttccaaaggctctttggctactggttcctacacagagaaaaccttagaatggtttgagaagcataccgatttttgctttggctttatagctggtcgtcgatttcctaaccttcaaagcgactacataactatgtcccctggtatcggcttggatgttaaaggagacgggctgggacagcaatatcgtactcctgaagaagtgattgtaaactgcggtagcgatatcatcattgttggtcgtggagtctatggagctggtcgtaatcctgttgtcgaagccaagagatatagagaagctggttggaaggcatatcagcaaagactttctcagcattaaaaaaagactaatgtaaaatttttttggttggttattgaaaaagtcgatgccttgtttgcgtttgttttcctaggcgttttatgtcagaaggcatttagaattagtatacaagtactctttggtaaaattttatgtagcgactaaaatattaactattatagataaacaccttgggaataaaaagtaatttgctatagtaatttattaaacatgctcctacaacattaccacaatcttttctcttggattgacattgaataagaaaagagtgaatttttttagacttgtaatgataactatgtacaaagccaatgaaagatgtatgtagatgaatgtaaaataccatgtagacaaacaagataaaacttggttataaacattggtgttggaacagaataaattagatgtcaaaaagtttcgtcaatatcacgatttgatttgacctaatttttattgattaattgtgtcgcatatttattatcgttaaaaatgcaattgacaaaagcgtaaattttaaggctctaatgttttttcttttggtatgttatagtcaagcaaagatgtattaagttgcaaaagcatggaattgatttacgacatccgttcaactttatgaccgagttgtcgctgtgtacttactctttttttactatgaaaagtttcaaacataatatcattaattatttctttttaaacgaagtaacctgtaacataaataataggggaaaagaagaaaaaaaccataaaaattcgagaacgtaacaattattattgtaacgatgacaacccgattctttgagagagccagagacatgacttttgcattaccgtaattggtttctacttatgaattgtaaacttgtgtagggaatttcatccacagctatcgcgggctgctctctgcatcagtgttagcacattttatattaatgaagattttgctatgcaaaatacgtacatttctggcgcatatatttttatcaaacgaacatgactatcctttccactttgatggttcgcaagtattttgatttgtattaaatgcacatctaacaagccatttcgcccaaaatcaccaactggcacatgtacccaaccagttggttcctcgacttgaactgttgtaacaatttccaaatcttgaaaccctgtccgcggccgcccagctgcattaatgaatcggccaacgcgcggggagaggcggtttgcgtattgggcgctcttccgcttcctcgctcactgactcgctgcgctcggtcgttcggctgcggcgagcggtatcagctcactcaaaggcggtaatacggttatccacagaatcaggggataacgcaggaaagaacatgtgagcaaaaggccagcaaaaggccaggaaccgtaaaaaggccgcgttgctggcgtttttccataggctccgcccccctgacgagcatcacaaaaatcgacgctcaagtcagaggtggcgaaacccgacaggactataaagataccaggcgtttccccctggaagctccctcgtgcgctctcctgttccgaccctgccgcttaccggatacctgtccgcctttctcccttcgggaagcgtggcgctttctcatagctcacgctgtaggtatctcagttcggtgtaggtcgttcgctccaagctgggctgtgtgcacgaaccccccgttcagcccgaccgctgcgccttatccggtaactatcgtcttgagtccaacccggtaagacacgacttatcgccactggcagcagccactggtaacaggattagcagagcgaggtatgtaggcggtgctacagagttcttgaagtggtggcctaactacggctacactagaagaacagtatttggtatctgcgctctgctgaagccagttaccttcggaaaaagagttggtagctcttgatccggcaaacaaaccaccgctggtagcggtggtttttttgtttgcaagcagcagattacgcgcagaaaaaaaggatctcaagaagatcctttgatcttttctacggggtctgacgctcagtggaacgaaaactcacgttaagggattttggtcatgagattatcaaaaaggatcttcacctagatccttttaaattaaaaatgaagttttaaatcaatctaaagtatatatgagtaaacttggtctgacagttaccaatgcttaatcagtgaggcacctatctcagcgatctgtctatttcgttcatccatagttgcctgactccccgtcgtgtagataactacgatacgggagggcttaccatctggccccagtgctgcaatgataccgcgagacccacgctcaccggctccagatttatcagcaataaaccagccagccggaagggccgagcgcagaagtggtcctgcaactttatccgcctccatccagtctattaattgttgccgggaagctagagtaagtagttcgccagttaatagtttgcgcaacgttgttgccattgctacaggcatcgtggtgtcacgctcgtcgtttggtatggcttcattcagctccggttcccaacgatcaaggcgagttacatgatcccccatgttgtgcaaaaaagcggttagctccttcggtcctccgatcgttgtcagaagtaagttggccgcagtgttatcactcatggttatggcagcactgcataattctcttactgtcatgccatccgtaagatgcttttctgtgactggtgagtactcaaccaagtcattctgagaatagtgtatgcggcgaccgagttgctcttgcccggcgtcaatacgggataataccgcgccacatagcagaactttaaaagtgctcatcattggaaaacgttcttcggggcgaaaactctcaaggatcttaccgctgttgagatccagttcgatgtaacccactcgtgcacccaactgatcttcagcatcttttactttcaccagcgtttctgggtgagcaaaaacaggaaggcaaaatgccgcaaaaaagggaataagggcgacacggaaatgttgaatactcatactcttcctttttcaatattattgaagcattt

Leu1 frag

nmt1 prom

MCS

nmt term

Ura4 frag

beta-lactamase (AmpR)
