# Supplementary material for: Extending the Schizosaccharomyces pombe Molecular Genetic Toolbox
Source: PLoS One. 2014 May 21;9(5):e97683. doi: 10.1371/journal.pone.0097683 (PMC4029729; doi:10.1371/journal.pone.0097683)
Supplement: Figure S2 — DNA sequence of pINTK. (DOCX) [file pone.0097683.s002.docx]

**pINTHA**

**gacgaaagggcctcgtgatacgcctatttttataggttaatgtcatgataataatggtttcttagacgtcaggtggcacttttcggggaaatgtgcgcggaacccctatttgtttatttttctaaatacattcaaatatgtatccgctcatgagacaataaccctgataaatgcttcaataatattgaaaaaggaagagtatgagtattcaacatttccgtgtcgcccttattcccttttttgcggcattttgccttcctgtttttgctcacccagaaacgctggtgaaagtaaaagatgctgaagatcagttgggtgcacgagtgggttacatcgaactggatctcaacagcggtaagatccttgagagttttcgccccgaagaacgttttccaatgatgagcacttttaaagttctgctatgtggcgcggtattatcccgtattgacgccgggcaagagcaactcggtcgccgcatacactattctcagaatgacttggttgagtactcaccagtcacagaaaagcatcttacggatggcatgacagtaagagaattatgcagtgctgccataaccatgagtgataacactgcggccaacttacttctgacaacgatcggaggaccgaaggagctaaccgcttttttgcacaacatgggggatcatgtaactcgccttgatcgttgggaaccggagctgaatgaagccataccaaacgacgagcgtgacaccacgatgcctgtagcaatggcaacaacgttgcgcaaactattaactggcgaactacttactctagcttcccggcaacaattaatagactggatggaggcggataaagttgcaggaccacttctgcgctcggcccttccggctggctggtttattgctgataaatctggagccggtgagcgtgggtctcgcggtatcattgcagcactggggccagatggtaagccctcccgtatcgtagttatctacacgacggggagtcaggcaactatggatgaacgaaatagacagatcgctgagataggtgcctcactgattaagcattggtaactgtcagaccaagtttactcatatatactttagattgatttaaaacttcatttttaatttaaaaggatctaggtgaagatcctttttgataatctcatgaccaaaatcccttaacgtgagttttcgttccactgagcgtcagaccccgtagaaaagatcaaaggatcttcttgagatcctttttttctgcgcgtaatctgctgcttgcaaacaaaaaaaccaccgctaccagcggtggtttgtttgccggatcaagagctaccaactctttttccgaaggtaactggcttcagcagagcgcagataccaaatactgttcttctagtgtagccgtagttaggccaccacttcaagaactctgtagcaccgcctacatacctcgctctgctaatcctgttaccagtggctgctgccagtggcgataagtcgtgtcttaccgggttggactcaagacgatagttaccggataaggcgcagcggtcgggctgaacggggggttcgtgcacacagcccagcttggagcgaacgacctacaccgaactgagatacctacagcgtgagctatgagaaagcgccacgcttcccgaagggagaaaggcggacaggtatccggtaagcggcagggtcggaacaggagagcgcacgagggagcttccagggggaaacgcctggtatctttatagtcctgtcgggtttcgccacctctgacttgagcgtcgatttttgtgatgctcgtcaggggggcggagcctatggaaaaacgccagcaacgcggcctttttacggttcctggccttttgctggccttttgctcacatgttctttcctgcgttatcccctgattctgtggataaccgtattaccgcctttgagtgagctgataccgctcgccgcagccgaacgaccgagcgcagcgagtcagtgagcgaggaagcggaagagcgcccaatacgcaaaccgcctctccccgcgcgttggccgattcattaatgcagctggcacgacaggtttcccgactggaaagcgggcagtgagcgcaacgcaattaatgtgagttagctcactcattaggcaccccaggctttacactttatgcttccggctcgtatgttgtgtggaattgtgagcggataacaatttcacacaggaaacagctatgaccatgattacgccaagcttgcggccgcgttaattaaggcgcgccagatctgtttagcttgcctcgtccccgccgggtcacccggccagcgacatggaggcccagaataccctccttgacagtcttgacgtgcgcagctcaggggcatgatgtgactgtcgcccgtacatttagcccatacatccccatgtataatcatttgcatccatacattttgatggccgcacggcgcgaagcaaaaattacggctcctcgctgcggacctgcgagcagggaaacgctcccctcacagacgcgttgaattgtccccacgccgcgcccctgtagagaaatataaaaggttaggatttgccactgaggttcttctttcatatacttccttttaaaatcttgctaggatacagttctcacatcacatccgaacataaacaaccatgggtaaaaagcctgaactcaccgcgacgtctgtcgagaagtttctgatcgaaaagttcgacagcgtctccgacctgatgcggctctcggagggcgaagaatctcgtgctttcagcttcgatgtaggagggcgtggatatgtcctgcgggtaaatagctgcgccgatggtttctacaaagatcgttatgtttatcggcactttgcatcggccgcgctcccgattccggaagtgcttgacattggggaattcagcgagagcctgacctattgcatctcccgccgtgcacagggtgtcacgttgcaagacctgcctgaaaccgaacctgcccgctgttctgcaaccggtcgcggaggccatggatgcgatcgctgcggccgatcttagccagacgagcgggttcggcccattcggaccgcaaggaatcctgcagtgcatattggagctcggcgcgccagatctgtttagcttgcctcgtccccgccgggtcacccggccagcgacatggaggcccagaataccctccttgacagtcttgacgtgcgcagctcaggggcatgatgtgactgtcgcccgtacatttagcccatacatccccatgtataatcatttgcatccatacattttgatggccgcacggcgcgaagcaaaaattacggctcctcgctgccgacctgcgagcagggaaacgctcccctcacagacgcgttgaattgtccccacgccgcgcccctgtagagaaatataaaaggttaggatttgccactgaggttcttctttcatatacttcctttttaaatcttgctaggatacagttctcacatcacatccgaacataaacaaccatgggtaccactcttgacgacacggcttaccggtaccgcaccagtgtcccaggggacgccgaggccatcgaggcactggatgggtccttcaccaccgacaccgtcttccgcgtcaccgccaccggggacggcttcaccctgcgggaggtgccggtggacccgcccctgaccaaggtgttccccgacgacgaatcggacgacgaatcggacgacggggaggacggcgacccggactctcggacgttcgtcgcgtacggggacgacggcgacctggcgggcttcgtggtcgtctcgtactccggctggaaccgccggctgaccgtcgaggacatcgaggtcgccccggagcaccgggggcacggggtcgggcgcgcgttgatggggctcgtgacggagttcgcccgcgagcggggtgccgggcacctctggctggaggtcaccaacgtcaacgcaccggcgatccacgcgtaccggcggatggggttcaccctctgcggcctggacaccgccctgtacgacggcaccgcctcggacggcgagcaggcgctctacatgagcatgccctgcccctaatcagtactgacaataaaaagattcttgttttcaagaacttgtcatttgtatagtttttttatattgtagttgttctattttaatcaaatgttagcgtgatttatatttttttcgcctcgacatcatctgcccagatgcgaagttaagtgcgcagaaagtaatatcatgcgtcaatcgtatgtgaatgctggtcgctatactgctgtcgattcgatactaacgccgccatccagaattcggtcaatacactacatggcgtgatttcatttgcgcgattgctgatccccatgtgtatcactggcaaactgtgatggacgacaccgtcggtgcgtccgtcgcgcaggctctcgatgagctgatgctttgggccgaggactgccccgaagtccggcacctcgtgcacgcggatttcggctccaacaatgtcctgacggacaatggccgcataacagcggtcattgactggagcgaggcgatgttcggggattcccaatacgaggtcgccaacatcttcttctggaggccgtggttggcttgtatggagcagcagacgcgctacttcgagcggaggcatccggagcttgcaggatcgccgcggctccgggcgtatatgctccgcattggtcttgaccaactctatcagagcttggttgacggcaatttcgatgatgcagcttgggcgcagggtcgatgcgacgcaatcgtccgatccggagccgggactgtcgggcgtacacaaatcgcccgcagaagcgcggccgtctggaccgatggctgtgtagaagtactcgccgatagtggaaaccgacgccccagcactcgtccgagggcaaaggaataatcagtactgacaataaaaagattcttgttttcaagaacttgtcatttgtatagtttttttatattgtagttgttctattttaatcaaatgttagcgtgatttatattttttttcgcctcgacatcatctgcccagatgcgaagttaagtgcgcagaaagtaatatcatgcgtcaatcgtatgtgaatgctggtcgctatactgctgtcgattcgatactaacgccgccatccagtttaaagcggccgcgaattcactggccgtcgttttacaacgtcgtgactgggaaaaccctggcgttacccaacttaatcgccttgcagcacatccccctttcgccagctggcgtaatagcgaagaggcccgcaccgatcgcccttcccaacagttgcgcagcctgaatggcgaatggcgcctgatgcggtattttctccttacgcatctgtgcggtatttcacaccgcatatatggtgcactctcagtacaatctgctctgatgccgcatagttaagccagccccgacacccgccaacacccgctgacgcgccctgacgggcttgtctgctcccggcatccgcttacagacaagctgtgaccgtctccgggagctgcatgtgtcagaggttttcaccgtcatcaccgaaacgcgcga**

hphMX6

ClonNat

beta-lactamase (AmpR)

**pINTH81**

gacgaaagggcctcgtgatacgcctatttttataggttaatgtcatgataataatggtttcttagacgtcaggtggcacttttcggggaaatgtgcgcggaacccctatttgtttatttttctaaatacattcaaatatgtatccgctcatgagacaataaccctgataaatgcttcaataatattgaaaaaggaagagtatgagtattcaacatttccgtgtcgcccttattcccttttttgcggcattttgccttcctgtttttgctcacccagaaacgctggtgaaagtaaaagatgctgaagatcagttgggtgcacgagtgggttacatcgaactggatctcaacagcggtaagatccttgagagttttcgccccgaagaacgttttccaatgatgagcacttttaaagttctgctatgtggcgcggtattatcccgtattgacgccgggcaagagcaactcggtcgccgcatacactattctcagaatgacttggttgagtactcaccagtcacagaaaagcatcttacggatggcatgacagtaagagaattatgcagtgctgccataaccatgagtgataacactgcggccaacttacttctgacaacgatcggaggaccgaaggagctaaccgcttttttgcacaacatgggggatcatgtaactcgccttgatcgttgggaaccggagctgaatgaagccataccaaacgacgagcgtgacaccacgatgcctgtagcaatggcaacaacgttgcgcaaactattaactggcgaactacttactctagcttcccggcaacaattaatagactggatggaggcggataaagttgcaggaccacttctgcgctcggcccttccggctggctggtttattgctgataaatctggagccggtgagcgtgggtctcgcggtatcattgcagcactggggccagatggtaagccctcccgtatcgtagttatctacacgacggggagtcaggcaactatggatgaacgaaatagacagatcgctgagataggtgcctcactgattaagcattggtaactgtcagaccaagtttactcatatatactttagattgatttaaaacttcatttttaatttaaaaggatctaggtgaagatcctttttgataatctcatgaccaaaatcccttaacgtgagttttcgttccactgagcgtcagaccccgtagaaaagatcaaaggatcttcttgagatcctttttttctgcgcgtaatctgctgcttgcaaacaaaaaaaccaccgctaccagcggtggtttgtttgccggatcaagagctaccaactctttttccgaaggtaactggcttcagcagagcgcagataccaaatactgttcttctagtgtagccgtagttaggccaccacttcaagaactctgtagcaccgcctacatacctcgctctgctaatcctgttaccagtggctgctgccagtggcgataagtcgtgtcttaccgggttggactcaagacgatagttaccggataaggcgcagcggtcgggctgaacggggggttcgtgcacacagcccagcttggagcgaacgacctacaccgaactgagatacctacagcgtgagctatgagaaagcgccacgcttcccgaagggagaaaggcggacaggtatccggtaagcggcagggtcggaacaggagagcgcacgagggagcttccagggggaaacgcctggtatctttatagtcctgtcgggtttcgccacctctgacttgagcgtcgatttttgtgatgctcgtcaggggggcggagcctatggaaaaacgccagcaacgcggcctttttacggttcctggccttttgctggccttttgctcacatgttctttcctgcgttatcccctgattctgtggataaccgtattaccgcctttgagtgagctgataccgctcgccgcagccgaacgaccgagcgcagcgagtcagtgagcgaggaagcggaagagcgcccaatacgcaaaccgcctctccccgcgcgttggccgattcattaatgcagctggcacgacaggtttcccgactggaaagcgggcagtgagcgcaacgcaattaatgtgagttagctcactcattaggcaccccaggctttacactttatgcttccggctcgtatgttgtgtggaattgtgagcggataacaatttcacacaggaaacagctatgaccatgattacgccaagcttgcggccgcgttaattaaggcgcgccagatctgtttagcttgcctcgtccccgccgggtcacccggccagcgacatggaggcccagaataccctccttgacagtcttgacgtgcgcagctcaggggcatgatgtgactgtcgcccgtacatttagcccatacatccccatgtataatcatttgcatccatacattttgatggccgcacggcgcgaagcaaaaattacggctcctcgctgcggacctgcgagcagggaaacgctcccctcacagacgcgttgaattgtccccacgccgcgcccctgtagagaaatataaaaggttaggatttgccactgaggttcttctttcatatacttccttttaaaatcttgctaggatacagttctcacatcacatccgaacataaacaaccatgggtaaaaagcctgaactcaccgcgacgtctgtcgagaagtttctgatcgaaaagttcgacagcgtctccgacctgatgcggctctcggagggcgaagaatctcgtgctttcagcttcgatgtaggagggcgtggatatgtcctgcgggtaaatagctgcgccgatggtttctacaaagatcgttatgtttatcggcactttgcatcggccgcgctcccgattccggaagtgcttgacattggggaattcagcgagagcctgacctattgcatctcccgccgtgcacagggtgtcacgttgcaagacctgcctgaaaccgaacctgcccgctgttctgcaaccggtcgcggaggccatggatgcgatcgctgcggccgatcttagccagacgagcgggttcggcccattcggaccgcaaggaatcctgcaggtcgatcgactctagaggatcagaaaattatcgccataaaagacagaataagtcatcagcggttgtttcatttcctatattttttttttatttttttattttttaataagggaaaatttaacgtctaaggatacagaagattgttagcacattaaagtaataaaggcttaagtagtaagtgccttagcatgttattgtatttcaaaggacataatctaaaataataacaatatcatttctcacaagttattcaattttcttttttttttctaataatatcaagaatgtattatttgtttgacataagtcaactaatttatttaatatgctggattaatcttgcagacatgtaaattaacaagttttagtcaaataacgttgaagtttcaatgaactcaaataatttctctttttttttatataaccataatctgatttatattttccgcagggatcaactgaagttatgacatttggattggatcacttataaccttggtcgccaaataatacaaaaatcagcgttataaaacaaagaaggtttttgttaagaaattaatcctctttcttgataagaaagttgaaccgaaattgcagatactgatatatgaaaataatacccacaattttgggaatagcgcaagcctcaatttaaacaataggtgaggacacatgataatgacctcaatgattgttagaagaaaagagcctcattacaaaatcgaaaaatgaatggttgggtacaagtttccaaaacatggtaaagtggactttgcgtatgagacgtaaatagaaaaaaacacttgttatatgttttctagaattattgttgtctctttatggttggatgatgcaaaatagtaatttcggttagttgctgtaaaacaccacgagacaaatagatatggatatttattaaatcaggaaaaacgtaactctcggctactggatggttcagtcacccaacgattactggggagagaaaacagggcaaaagcaaagcttaaaggaatccgattgtcattcggcaatgtgcagcgaaactaaaaaccggataatggacctgttaatcgaaacattgaagatggaagaggaatcctggcatatcatcaattgaataagttgaattaattatttcaatctcattctcactttctgacttatagtcgctttgttaaatcatatgtcgactctagaggatccccgggtaaaaggaatgtctcccttgccagtactgctagggtttttctttcaaactatggaagcccattcaagctgcatattacgattttgtttttcgcttttagaaagtggtttagatgagataatagaaaaattcttgatctccgacaacgagtacttttattttttttgctaatcactttactcaatattagctcgaaatcgtagaaacgtagacgggtgcgggataccgagtggtgtagttaagaatttttataaaccacgtggcccaaaaatatgaacccaaaacgtttatacatgagtatactttaagaaggctataccccttcgtgttagatgtagttttagctacccaacccgagtctatgagcttgacttcagatgtagaaggcattaaatcgttttgaatattaattaaaaaacgatgaaaattaaatatttaaaagcaatcatacgctgaaaatttagtgctgtggctaatccttcaacatggaaatgccataaaagtgactttgacaaaaaaaaaagtatatacaggtagtaaactcatctacttcattgactttgtttacagcatgtggaaggaggaatatttattgctaaatcgtagtttaacattcaataagtaatactattgaaattcgacaagattggccgcatggatgaaaaagaggcattttgctttgggagaattagttcaaattagaactgaaaaaaaaaactttacgaggcaaaaatgtcggattgagatcgtaaaagttcgctcgtcgtcttttgctttgtgattgttttcatggatacatcttgctggatatttaaattttagtactatgtataagatattctataaatgttttatcacccaaacctgttagcgccttcttaattctattcaatctggcttttgctctgagactacttcttggactttcactacttgttagttatacggaatttgtgtaattagaagtgaaataatcctttctattagtaatgcgagctcggcgcgccagatctgtttagcttgcctcgtccccgccgggtcacccggccagcgacatggaggcccagaataccctccttgacagtcttgacgtgcgcagctcaggggcatgatgtgactgtcgcccgtacatttagcccatacatccccatgtataatcatttgcatccatacattttgatggccgcacggcgcgaagcaaaaattacggctcctcgctgccgacctgcgagcagggaaacgctcccctcacagacgcgttgaattgtccccacgccgcgcccctgtagagaaatataaaaggttaggatttgccactgaggttcttctttcatatacttcctttttaaatcttgctaggatacagttctcacatcacatccgaacataaacaaccatgggtaccactcttgacgacacggcttaccggtaccgcaccagtgtcccaggggacgccgaggccatcgaggcactggatgggtccttcaccaccgacaccgtcttccgcgtcaccgccaccggggacggcttcaccctgcgggaggtgccggtggacccgcccctgaccaaggtgttccccgacgacgaatcggacgacgaatcggacgacggggaggacggcgacccggactctcggacgttcgtcgcgtacggggacgacggcgacctggcgggcttcgtggtcgtctcgtactccggctggaaccgccggctgaccgtcgaggacatcgaggtcgccccggagcaccgggggcacggggtcgggcgcgcgttgatggggctcgtgacggagttcgcccgcgagcggggtgccgggcacctctggctggaggtcaccaacgtcaacgcaccggcgatccacgcgtaccggcggatggggttcaccctctgcggcctggacaccgccctgtacgacggcaccgcctcggacggcgagcaggcgctctacatgagcatgccctgcccctaatcagtactgacaataaaaagattcttgttttcaagaacttgtcatttgtatagtttttttatattgtagttgttctattttaatcaaatgttagcgtgatttatatttttttcgcctcgacatcatctgcccagatgcgaagttaagtgcgcagaaagtaatatcatgcgtcaatcgtatgtgaatgctggtcgctatactgctgtcgattcgatactaacgccgccatccagaattcggtcaatacactacatggcgtgatttcatttgcgcgattgctgatccccatgtgtatcactggcaaactgtgatggacgacaccgtcggtgcgtccgtcgcgcaggctctcgatgagctgatgctttgggccgaggactgccccgaagtccggcacctcgtgcacgcggatttcggctccaacaatgtcctgacggacaatggccgcataacagcggtcattgactggagcgaggcgatgttcggggattcccaatacgaggtcgccaacatcttcttctggaggccgtggttggcttgtatggagcagcagacgcgctacttcgagcggaggcatccggagcttgcaggatcgccgcggctccgggcgtatatgctccgcattggtcttgaccaactctatcagagcttggttgacggcaatttcgatgatgcagcttgggcgcagggtcgatgcgacgcaatcgtccgatccggagccgggactgtcgggcgtacacaaatcgcccgcagaagcgcggccgtctggaccgatggctgtgtagaagtactcgccgatagtggaaaccgacgccccagcactcgtccgagggcaaaggaataatcagtactgacaataaaaagattcttgttttcaagaacttgtcatttgtatagtttttttatattgtagttgttctattttaatcaaatgttagcgtgatttatattttttttcgcctcgacatcatctgcccagatgcgaagttaagtgcgcagaaagtaatatcatgcgtcaatcgtatgtgaatgctggtcgctatactgctgtcgattcgatactaacgccgccatccagtttaaagcggccgcgaattcactggccgtcgttttacaacgtcgtgactgggaaaaccctggcgttacccaacttaatcgccttgcagcacatccccctttcgccagctggcgtaatagcgaagaggcccgcaccgatcgcccttcccaacagttgcgcagcctgaatggcgaatggcgcccgggagctgcatgtgtcagaggttttcaccgtcatcaccgaaacgcgcga

hphMX6

nmt81 prom

MCS

nmt term

ClonNat

beta-lactamase (AmpR)

**pINTH81PkN**

gacgaaagggcctcgtgatacgcctatttttataggttaatgtcatgataataatggtttcttagacgtcaggtggcacttttcggggaaatgtgcgcggaacccctatttgtttatttttctaaatacattcaaatatgtatccgctcatgagacaataaccctgataaatgcttcaataatattgaaaaaggaagagtatgagtattcaacatttccgtgtcgcccttattcccttttttgcggcattttgccttcctgtttttgctcacccagaaacgctggtgaaagtaaaagatgctgaagatcagttgggtgcacgagtgggttacatcgaactggatctcaacagcggtaagatccttgagagttttcgccccgaagaacgttttccaatgatgagcacttttaaagttctgctatgtggcgcggtattatcccgtattgacgccgggcaagagcaactcggtcgccgcatacactattctcagaatgacttggttgagtactcaccagtcacagaaaagcatcttacggatggcatgacagtaagagaattatgcagtgctgccataaccatgagtgataacactgcggccaacttacttctgacaacgatcggaggaccgaaggagctaaccgcttttttgcacaacatgggggatcatgtaactcgccttgatcgttgggaaccggagctgaatgaagccataccaaacgacgagcgtgacaccacgatgcctgtagcaatggcaacaacgttgcgcaaactattaactggcgaactacttactctagcttcccggcaacaattaatagactggatggaggcggataaagttgcaggaccacttctgcgctcggcccttccggctggctggtttattgctgataaatctggagccggtgagcgtgggtctcgcggtatcattgcagcactggggccagatggtaagccctcccgtatcgtagttatctacacgacggggagtcaggcaactatggatgaacgaaatagacagatcgctgagataggtgcctcactgattaagcattggtaactgtcagaccaagtttactcatatatactttagattgatttaaaacttcatttttaatttaaaaggatctaggtgaagatcctttttgataatctcatgaccaaaatcccttaacgtgagttttcgttccactgagcgtcagaccccgtagaaaagatcaaaggatcttcttgagatcctttttttctgcgcgtaatctgctgcttgcaaacaaaaaaaccaccgctaccagcggtggtttgtttgccggatcaagagctaccaactctttttccgaaggtaactggcttcagcagagcgcagataccaaatactgttcttctagtgtagccgtagttaggccaccacttcaagaactctgtagcaccgcctacatacctcgctctgctaatcctgttaccagtggctgctgccagtggcgataagtcgtgtcttaccgggttggactcaagacgatagttaccggataaggcgcagcggtcgggctgaacggggggttcgtgcacacagcccagcttggagcgaacgacctacaccgaactgagatacctacagcgtgagctatgagaaagcgccacgcttcccgaagggagaaaggcggacaggtatccggtaagcggcagggtcggaacaggagagcgcacgagggagcttccagggggaaacgcctggtatctttatagtcctgtcgggtttcgccacctctgacttgagcgtcgatttttgtgatgctcgtcaggggggcggagcctatggaaaaacgccagcaacgcggcctttttacggttcctggccttttgctggccttttgctcacatgttctttcctgcgttatcccctgattctgtggataaccgtattaccgcctttgagtgagctgataccgctcgccgcagccgaacgaccgagcgcagcgagtcagtgagcgaggaagcggaagagcgcccaatacgcaaaccgcctctccccgcgcgttggccgattcattaatgcagctggcacgacaggtttcccgactggaaagcgggcagtgagcgcaacgcaattaatgtgagttagctcactcattaggcaccccaggctttacactttatgcttccggctcgtatgttgtgtggaattgtgagcggataacaatttcacacaggaaacagctatgaccatgattacgccaagcttgcggccgcgttaattaaggcgcgccagatctgtttagcttgcctcgtccccgccgggtcacccggccagcgacatggaggcccagaataccctccttgacagtcttgacgtgcgcagctcaggggcatgatgtgactgtcgcccgtacatttagcccatacatccccatgtataatcatttgcatccatacattttgatggccgcacggcgcgaagcaaaaattacggctcctcgctgcggacctgcgagcagggaaacgctcccctcacagacgcgttgaattgtccccacgccgcgcccctgtagagaaatataaaaggttaggatttgccactgaggttcttctttcatatacttccttttaaaatcttgctaggatacagttctcacatcacatccgaacataaacaaccatgggtaaaaagcctgaactcaccgcgacgtctgtcgagaagtttctgatcgaaaagttcgacagcgtctccgacctgatgcggctctcggagggcgaagaatctcgtgctttcagcttcgatgtaggagggcgtggatatgtcctgcgggtaaatagctgcgccgatggtttctacaaagatcgttatgtttatcggcactttgcatcggccgcgctcccgattccggaagtgcttgacattggggaattcagcgagagcctgacctattgcatctcccgccgtgcacagggtgtcacgttgcaagacctgcctgaaaccgaacctgcccgctgttctgcaaccggtcgcggaggccatggatgcgatcgctgcggccgatcttagccagacgagcgggttcggcccattcggaccgcaaggaatcctgcaggtcgatcgactctagaggatcagaaaattatcgccataaaagacagaataagtcatcagcggttgtttcatttcctatattttttttttatttttttattttttaataagggaaaatttaacgtctaaggatacagaagattgttagcacattaaagtaataaaggcttaagtagtaagtgccttagcatgttattgtatttcaaaggacataatctaaaataataacaatatcatttctcacaagttattcaattttcttttttttttctaataatatcaagaatgtattatttgtttgacataagtcaactaatttatttaatatgctggattaatcttgcagacatgtaaattaacaagttttagtcaaataacgttgaagtttcaatgaactcaaataatttctctttttttttatataaccataatctgatttatattttccgcagggatcaactgaagttatgacatttggattggatcacttataaccttggtcgccaaataatacaaaaatcagcgttataaaacaaagaaggtttttgttaagaaattaatcctctttcttgataagaaagttgaaccgaaattgcagatactgatatatgaaaataatacccacaattttgggaatagcgcaagcctcaatttaaacaataggtgaggacacatgataatgacctcaatgattgttagaagaaaagagcctcattacaaaatcgaaaaatgaatggttgggtacaagtttccaaaacatggtaaagtggactttgcgtatgagacgtaaatagaaaaaaacacttgttatatgttttctagaattattgttgtctctttatggttggatgatgcaaaatagtaatttcggttagttgctgtaaaacaccacgagacaaatagatatggatatttattaaatcaggaaaaacgtaactctcggctactggatggttcagtcacccaacgattactggggagagaaaacagggcaaaagcaaagcttaaaggaatccgattgtcattcggcaatgtgcagcgaaactaaaaaccggataatggacctgttaatcgaaacattgaagatggaagaggaatcctggcatatcatcaattgaataagttgaattaattatttcaatctcattctcactttctgacttatagtcgctttgttaaatcatatcgagctcatgggtattcctaaccctttgcttggtcttgatggtattcctaaccctttgcttggtcttgattccatgggtattcctaaccctttgcttggtcttgatcatatgtcgacaggtaccagatctcgagggatccccgggtaaaaggaatgtctcccttgccagtactgctagggtttttctttcaaactatggaagcccattcaagctgcatattacgattttgtttttcgcttttagaaagtggtttagatgagataatagaaaaattcttgatctccgacaacgagtacttttattttttttgctaatcactttactcaatattagctcgaaatcgtagaaacgtagacgggtgcgggataccgagtggtgtagttaagaatttttataaaccacgtggcccaaaaatatgaacccaaaacgtttatacatgagtatactttaagaaggctataccccttcgtgttagatgtagttttagctacccaacccgagtctatgagcttgacttcagatgtagaaggcattaaatcgttttgaatattaattaaaaaacgatgaaaattaaatatttaaaagcaatcatacgctgaaaatttagtgctgtggctaatccttcaacatggaaatgccataaaagtgactttgacaaaaaaaaaagtatatacaggtagtaaactcatctacttcattgactttgtttacagcatgtggaaggaggaatatttattgctaaatcgtagtttaacattcaataagtaatactattgaaattcgacaagattggccgcatggatgaaaaagaggcattttgctttgggagaattagttcaaattagaactgaaaaaaaaaactttacgaggcaaaaatgtcggattgagatcgtaaaagttcgctcgtcgtcttttgctttgtgattgttttcatggatacatcttgctggatatttaaattttagtactatgtataagatattctataaatgttttatcacccaaacctgttagcgccttcttaattctattcaatctggcttttgctctgagactacttcttggactttcactacttgttagttatacggaatttgtgtaattagaagtgaaataatcctttctattagtaatgcgagctcggcgcgccagatctgtttagcttgcctcgtccccgccgggtcacccggccagcgacatggaggcccagaataccctccttgacagtcttgacgtgcgcagctcaggggcatgatgtgactgtcgcccgtacatttagcccatacatccccatgtataatcatttgcatccatacattttgatggccgcacggcgcgaagcaaaaattacggctcctcgctgccgacctgcgagcagggaaacgctcccctcacagacgcgttgaattgtccccacgccgcgcccctgtagagaaatataaaaggttaggatttgccactgaggttcttctttcatatacttcctttttaaatcttgctaggatacagttctcacatcacatccgaacataaacaaccatgggtaccactcttgacgacacggcttaccggtaccgcaccagtgtcccaggggacgccgaggccatcgaggcactggatgggtccttcaccaccgacaccgtcttccgcgtcaccgccaccggggacggcttcaccctgcgggaggtgccggtggacccgcccctgaccaaggtgttccccgacgacgaatcggacgacgaatcggacgacggggaggacggcgacccggactctcggacgttcgtcgcgtacggggacgacggcgacctggcgggcttcgtggtcgtctcgtactccggctggaaccgccggctgaccgtcgaggacatcgaggtcgccccggagcaccgggggcacggggtcgggcgcgcgttgatggggctcgtgacggagttcgcccgcgagcggggtgccgggcacctctggctggaggtcaccaacgtcaacgcaccggcgatccacgcgtaccggcggatggggttcaccctctgcggcctggacaccgccctgtacgacggcaccgcctcggacggcgagcaggcgctctacatgagcatgccctgcccctaatcagtactgacaataaaaagattcttgttttcaagaacttgtcatttgtatagtttttttatattgtagttgttctattttaatcaaatgttagcgtgatttatatttttttcgcctcgacatcatctgcccagatgcgaagttaagtgcgcagaaagtaatatcatgcgtcaatcgtatgtgaatgctggtcgctatactgctgtcgattcgatactaacgccgccatccagaattcggtcaatacactacatggcgtgatttcatttgcgcgattgctgatccccatgtgtatcactggcaaactgtgatggacgacaccgtcggtgcgtccgtcgcgcaggctctcgatgagctgatgctttgggccgaggactgccccgaagtccggcacctcgtgcacgcggatttcggctccaacaatgtcctgacggacaatggccgcataacagcggtcattgactggagcgaggcgatgttcggggattcccaatacgaggtcgccaacatcttcttctggaggccgtggttggcttgtatggagcagcagacgcgctacttcgagcggaggcatccggagcttgcaggatcgccgcggctccgggcgtatatgctccgcattggtcttgaccaactctatcagagcttggttgacggcaatttcgatgatgcagcttgggcgcagggtcgatgcgacgcaatcgtccgatccggagccgggactgtcgggcgtacacaaatcgcccgcagaagcgcggccgtctggaccgatggctgtgtagaagtactcgccgatagtggaaaccgacgccccagcactcgtccgagggcaaaggaataatcagtactgacaataaaaagattcttgttttcaagaacttgtcatttgtatagtttttttatattgtagttgttctattttaatcaaatgttagcgtgatttatattttttttcgcctcgacatcatctgcccagatgcgaagttaagtgcgcagaaagtaatatcatgcgtcaatcgtatgtgaatgctggtcgctatactgctgtcgattcgatactaacgccgccatccagtttaaagcggccgcgaattcactggccgtcgttttacaacgtcgtgactgggaaaaccctggcgttacccaacttaatcgccttgcagcacatccccctttcgccagctggcgtaatagcgaagaggcccgcaccgatcgcccttcccaacagttgcgcagcctgaatggcgaatggcgcctgatgcggtattttctccttacgcatctgtgcggtatttcacaccgcatatatggtgcactctcagtacaatctgctctgatgccgcatagttaagccagccccgacacccgccaacacccgctgacgcgccctgacgggcttgtctgctcccggcatccgcttacagacaagctgtgaccgtctccgggagctgcatgtgtcagaggttttcaccgtcatcaccgaaacgcgcga

hphMX6

nmt81 prom

MCS

PK tag

nmt term

ClonNat

beta-lactamase (AmpR)

**pINTH81PkC**

gacgaaagggcctcgtgatacgcctatttttataggttaatgtcatgataataatggtttcttagacgtcaggtggcacttttcggggaaatgtgcgcggaacccctatttgtttatttttctaaatacattcaaatatgtatccgctcatgagacaataaccctgataaatgcttcaataatattgaaaaaggaagagtatgagtattcaacatttccgtgtcgcccttattcccttttttgcggcattttgccttcctgtttttgctcacccagaaacgctggtgaaagtaaaagatgctgaagatcagttgggtgcacgagtgggttacatcgaactggatctcaacagcggtaagatccttgagagttttcgccccgaagaacgttttccaatgatgagcacttttaaagttctgctatgtggcgcggtattatcccgtattgacgccgggcaagagcaactcggtcgccgcatacactattctcagaatgacttggttgagtactcaccagtcacagaaaagcatcttacggatggcatgacagtaagagaattatgcagtgctgccataaccatgagtgataacactgcggccaacttacttctgacaacgatcggaggaccgaaggagctaaccgcttttttgcacaacatgggggatcatgtaactcgccttgatcgttgggaaccggagctgaatgaagccataccaaacgacgagcgtgacaccacgatgcctgtagcaatggcaacaacgttgcgcaaactattaactggcgaactacttactctagcttcccggcaacaattaatagactggatggaggcggataaagttgcaggaccacttctgcgctcggcccttccggctggctggtttattgctgataaatctggagccggtgagcgtgggtctcgcggtatcattgcagcactggggccagatggtaagccctcccgtatcgtagttatctacacgacggggagtcaggcaactatggatgaacgaaatagacagatcgctgagataggtgcctcactgattaagcattggtaactgtcagaccaagtttactcatatatactttagattgatttaaaacttcatttttaatttaaaaggatctaggtgaagatcctttttgataatctcatgaccaaaatcccttaacgtgagttttcgttccactgagcgtcagaccccgtagaaaagatcaaaggatcttcttgagatcctttttttctgcgcgtaatctgctgcttgcaaacaaaaaaaccaccgctaccagcggtggtttgtttgccggatcaagagctaccaactctttttccgaaggtaactggcttcagcagagcgcagataccaaatactgttcttctagtgtagccgtagttaggccaccacttcaagaactctgtagcaccgcctacatacctcgctctgctaatcctgttaccagtggctgctgccagtggcgataagtcgtgtcttaccgggttggactcaagacgatagttaccggataaggcgcagcggtcgggctgaacggggggttcgtgcacacagcccagcttggagcgaacgacctacaccgaactgagatacctacagcgtgagctatgagaaagcgccacgcttcccgaagggagaaaggcggacaggtatccggtaagcggcagggtcggaacaggagagcgcacgagggagcttccagggggaaacgcctggtatctttatagtcctgtcgggtttcgccacctctgacttgagcgtcgatttttgtgatgctcgtcaggggggcggagcctatggaaaaacgccagcaacgcggcctttttacggttcctggccttttgctggccttttgctcacatgttctttcctgcgttatcccctgattctgtggataaccgtattaccgcctttgagtgagctgataccgctcgccgcagccgaacgaccgagcgcagcgagtcagtgagcgaggaagcggaagagcgcccaatacgcaaaccgcctctccccgcgcgttggccgattcattaatgcagctggcacgacaggtttcccgactggaaagcgggcagtgagcgcaacgcaattaatgtgagttagctcactcattaggcaccccaggctttacactttatgcttccggctcgtatgttgtgtggaattgtgagcggataacaatttcacacaggaaacagctatgaccatgattacgccaagcttgcggccgcgttaattaaggcgcgccagatctgtttagcttgcctcgtccccgccgggtcacccggccagcgacatggaggcccagaataccctccttgacagtcttgacgtgcgcagctcaggggcatgatgtgactgtcgcccgtacatttagcccatacatccccatgtataatcatttgcatccatacattttgatggccgcacggcgcgaagcaaaaattacggctcctcgctgcggacctgcgagcagggaaacgctcccctcacagacgcgttgaattgtccccacgccgcgcccctgtagagaaatataaaaggttaggatttgccactgaggttcttctttcatatacttccttttaaaatcttgctaggatacagttctcacatcacatccgaacataaacaaccatgggtaaaaagcctgaactcaccgcgacgtctgtcgagaagtttctgatcgaaaagttcgacagcgtctccgacctgatgcggctctcggagggcgaagaatctcgtgctttcagcttcgatgtaggagggcgtggatatgtcctgcgggtaaatagctgcgccgatggtttctacaaagatcgttatgtttatcggcactttgcatcggccgcgctcccgattccggaagtgcttgacattggggaattcagcgagagcctgacctattgcatctcccgccgtgcacagggtgtcacgttgcaagacctgcctgaaaccgaacctgcccgctgttctgcaaccggtcgcggaggccatggatgcgatcgctgcggccgatcttagccagacgagcgggttcggcccattcggaccgcaaggaatcctgcaggtcgatcgactctagaggatcagaaaattatcgccataaaagacagaataagtcatcagcggttgtttcatttcctatattttttttttatttttttattttttaataagggaaaatttaacgtctaaggatacagaagattgttagcacattaaagtaataaaggcttaagtagtaagtgccttagcatgttattgtatttcaaaggacataatctaaaataataacaatatcatttctcacaagttattcaattttcttttttttttctaataatatcaagaatgtattatttgtttgacataagtcaactaatttatttaatatgctggattaatcttgcagacatgtaaattaacaagttttagtcaaataacgttgaagtttcaatgaactcaaataatttctctttttttttatataaccataatctgatttatattttccgcagggatcaactgaagttatgacatttggattggatcacttataaccttggtcgccaaataatacaaaaatcagcgttataaaacaaagaaggtttttgttaagaaattaatcctctttcttgataagaaagttgaaccgaaattgcagatactgatatatgaaaataatacccacaattttgggaatagcgcaagcctcaatttaaacaataggtgaggacacatgataatgacctcaatgattgttagaagaaaagagcctcattacaaaatcgaaaaatgaatggttgggtacaagtttccaaaacatggtaaagtggactttgcgtatgagacgtaaatagaaaaaaacacttgttatatgttttctagaattattgttgtctctttatggttggatgatgcaaaatagtaatttcggttagttgctgtaaaacaccacgagacaaatagatatggatatttattaaatcaggaaaaacgtaactctcggctactggatggttcagtcacccaacgattactggggagagaaaacagggcaaaagcaaagcttaaaggaatccgattgtcattcggcaatgtgcagcgaaactaaaaaccggataatggacctgttaatcgaaacattgaagatggaagaggaatcctggcatatcatcaattgaataagttgaattaattatttcaatctcattctcactttctgacttatagtcgctttgttaaatcatatgtcgacatggtaccagatctctcgaggatccccgggtatgggtattcctaaccctttgcttggtcttgatggtattcctaaccctttgcttggtcttgattccatgggtattcctaaccctttgcttggtcttgatcattaaggatcgggtaaaaggaatgtctcccttgccagtactgctagggtttttctttcaaactatggaagcccattcaagctgcatattacgattttgtttttcgcttttagaaagtggtttagatgagataatagaaaaattcttgatctccgacaacgagtacttttattttttttgctaatcactttactcaatattagctcgaaatcgtagaaacgtagacgggtgcgggataccgagtggtgtagttaagaatttttataaaccacgtggcccaaaaatatgaacccaaaacgtttatacatgagtatactttaagaaggctataccccttcgtgttagatgtagttttagctacccaacccgagtctatgagcttgacttcagatgtagaaggcattaaatcgttttgaatattaattaaaaaacgatgaaaattaaatatttaaaagcaatcatacgctgaaaatttagtgctgtggctaatccttcaacatggaaatgccataaaagtgactttgacaaaaaaaaaagtatatacaggtagtaaactcatctacttcattgactttgtttacagcatgtggaaggaggaatatttattgctaaatcgtagtttaacattcaataagtaatactattgaaattcgacaagattggccgcatggatgaaaaagaggcattttgctttgggagaattagttcaaattagaactgaaaaaaaaaactttacgaggcaaaaatgtcggattgagatcgtaaaagttcgctcgtcgtcttttgctttgtgattgttttcatggatacatcttgctggatatttaaattttagtactatgtataagatattctataaatgttttatcacccaaacctgttagcgccttcttaattctattcaatctggcttttgctctgagactacttcttggactttcactacttgttagttatacggaatttgtgtaattagaagtgaaataatcctttctattagtaatgcgagctcggcgcgccagatctgtttagcttgcctcgtccccgccgggtcacccggccagcgacatggaggcccagaataccctccttgacagtcttgacgtgcgcagctcaggggcatgatgtgactgtcgcccgtacatttagcccatacatccccatgtataatcatttgcatccatacattttgatggccgcacggcgcgaagcaaaaattacggctcctcgctgccgacctgcgagcagggaaacgctcccctcacagacgcgttgaattgtccccacgccgcgcccctgtagagaaatataaaaggttaggatttgccactgaggttcttctttcatatacttcctttttaaatcttgctaggatacagttctcacatcacatccgaacataaacaaccatgggtaccactcttgacgacacggcttaccggtaccgcaccagtgtcccaggggacgccgaggccatcgaggcactggatgggtccttcaccaccgacaccgtcttccgcgtcaccgccaccggggacggcttcaccctgcgggaggtgccggtggacccgcccctgaccaaggtgttccccgacgacgaatcggacgacgaatcggacgacggggaggacggcgacccggactctcggacgttcgtcgcgtacggggacgacggcgacctggcgggcttcgtggtcgtctcgtactccggctggaaccgccggctgaccgtcgaggacatcgaggtcgccccggagcaccgggggcacggggtcgggcgcgcgttgatggggctcgtgacggagttcgcccgcgagcggggtgccgggcacctctggctggaggtcaccaacgtcaacgcaccggcgatccacgcgtaccggcggatggggttcaccctctgcggcctggacaccgccctgtacgacggcaccgcctcggacggcgagcaggcgctctacatgagcatgccctgcccctaatcagtactgacaataaaaagattcttgttttcaagaacttgtcatttgtatagtttttttatattgtagttgttctattttaatcaaatgttagcgtgatttatatttttttcgcctcgacatcatctgcccagatgcgaagttaagtgcgcagaaagtaatatcatgcgtcaatcgtatgtgaatgctggtcgctatactgctgtcgattcgatactaacgccgccatccagaattcggtcaatacactacatggcgtgatttcatttgcgcgattgctgatccccatgtgtatcactggcaaactgtgatggacgacaccgtcggtgcgtccgtcgcgcaggctctcgatgagctgatgctttgggccgaggactgccccgaagtccggcacctcgtgcacgcggatttcggctccaacaatgtcctgacggacaatggccgcataacagcggtcattgactggagcgaggcgatgttcggggattcccaatacgaggtcgccaacatcttcttctggaggccgtggttggcttgtatggagcagcagacgcgctacttcgagcggaggcatccggagcttgcaggatcgccgcggctccgggcgtatatgctccgcattggtcttgaccaactctatcagagcttggttgacggcaatttcgatgatgcagcttgggcgcagggtcgatgcgacgcaatcgtccgatccggagccgggactgtcgggcgtacacaaatcgcccgcagaagcgcggccgtctggaccgatggctgtgtagaagtactcgccgatagtggaaaccgacgccccagcactcgtccgagggcaaaggaataatcagtactgacaataaaaagattcttgttttcaagaacttgtcatttgtatagtttttttatattgtagttgttctattttaatcaaatgttagcgtgatttatattttttttcgcctcgacatcatctgcccagatgcgaagttaagtgcgcagaaagtaatatcatgcgtcaatcgtatgtgaatgctggtcgctatactgctgtcgattcgatactaacgccgccatccagtttaaagcggccgcgaattcactggccgtcgttttacaacgtcgtgactgggaaaaccctggcgttacccaacttaatcgccttgcagcacatccccctttcgccagctggcgtaatagcgaagaggcccgcaccgatcgcccttcccaacagttgcgcagcctgaatggcgaatggcgcccgggagctgcatgtgtcagaggttttcaccgtcatcaccgaaacgcgcga

hphMX6

nmt81 prom

MCS

PK tag

nmt term

ClonNat

beta-lactamase (AmpR)

**pINTH41**

gacgaaagggcctcgtgatacgcctatttttataggttaatgtcatgataataatggtttcttagacgtcaggtggcacttttcggggaaatgtgcgcggaacccctatttgtttatttttctaaatacattcaaatatgtatccgctcatgagacaataaccctgataaatgcttcaataatattgaaaaaggaagagtatgagtattcaacatttccgtgtcgcccttattcccttttttgcggcattttgccttcctgtttttgctcacccagaaacgctggtgaaagtaaaagatgctgaagatcagttgggtgcacgagtgggttacatcgaactggatctcaacagcggtaagatccttgagagttttcgccccgaagaacgttttccaatgatgagcacttttaaagttctgctatgtggcgcggtattatcccgtattgacgccgggcaagagcaactcggtcgccgcatacactattctcagaatgacttggttgagtactcaccagtcacagaaaagcatcttacggatggcatgacagtaagagaattatgcagtgctgccataaccatgagtgataacactgcggccaacttacttctgacaacgatcggaggaccgaaggagctaaccgcttttttgcacaacatgggggatcatgtaactcgccttgatcgttgggaaccggagctgaatgaagccataccaaacgacgagcgtgacaccacgatgcctgtagcaatggcaacaacgttgcgcaaactattaactggcgaactacttactctagcttcccggcaacaattaatagactggatggaggcggataaagttgcaggaccacttctgcgctcggcccttccggctggctggtttattgctgataaatctggagccggtgagcgtgggtctcgcggtatcattgcagcactggggccagatggtaagccctcccgtatcgtagttatctacacgacggggagtcaggcaactatggatgaacgaaatagacagatcgctgagataggtgcctcactgattaagcattggtaactgtcagaccaagtttactcatatatactttagattgatttaaaacttcatttttaatttaaaaggatctaggtgaagatcctttttgataatctcatgaccaaaatcccttaacgtgagttttcgttccactgagcgtcagaccccgtagaaaagatcaaaggatcttcttgagatcctttttttctgcgcgtaatctgctgcttgcaaacaaaaaaaccaccgctaccagcggtggtttgtttgccggatcaagagctaccaactctttttccgaaggtaactggcttcagcagagcgcagataccaaatactgttcttctagtgtagccgtagttaggccaccacttcaagaactctgtagcaccgcctacatacctcgctctgctaatcctgttaccagtggctgctgccagtggcgataagtcgtgtcttaccgggttggactcaagacgatagttaccggataaggcgcagcggtcgggctgaacggggggttcgtgcacacagcccagcttggagcgaacgacctacaccgaactgagatacctacagcgtgagctatgagaaagcgccacgcttcccgaagggagaaaggcggacaggtatccggtaagcggcagggtcggaacaggagagcgcacgagggagcttccagggggaaacgcctggtatctttatagtcctgtcgggtttcgccacctctgacttgagcgtcgatttttgtgatgctcgtcaggggggcggagcctatggaaaaacgccagcaacgcggcctttttacggttcctggccttttgctggccttttgctcacatgttctttcctgcgttatcccctgattctgtggataaccgtattaccgcctttgagtgagctgataccgctcgccgcagccgaacgaccgagcgcagcgagtcagtgagcgaggaagcggaagagcgcccaatacgcaaaccgcctctccccgcgcgttggccgattcattaatgcagctggcacgacaggtttcccgactggaaagcgggcagtgagcgcaacgcaattaatgtgagttagctcactcattaggcaccccaggctttacactttatgcttccggctcgtatgttgtgtggaattgtgagcggataacaatttcacacaggaaacagctatgaccatgattacgccaagcttgcggccgcgttaattaaggcgcgccagatctgtttagcttgcctcgtccccgccgggtcacccggccagcgacatggaggcccagaataccctccttgacagtcttgacgtgcgcagctcaggggcatgatgtgactgtcgcccgtacatttagcccatacatccccatgtataatcatttgcatccatacattttgatggccgcacggcgcgaagcaaaaattacggctcctcgctgcggacctgcgagcagggaaacgctcccctcacagacgcgttgaattgtccccacgccgcgcccctgtagagaaatataaaaggttaggatttgccactgaggttcttctttcatatacttccttttaaaatcttgctaggatacagttctcacatcacatccgaacataaacaaccatgggtaaaaagcctgaactcaccgcgacgtctgtcgagaagtttctgatcgaaaagttcgacagcgtctccgacctgatgcggctctcggagggcgaagaatctcgtgctttcagcttcgatgtaggagggcgtggatatgtcctgcgggtaaatagctgcgccgatggtttctacaaagatcgttatgtttatcggcactttgcatcggccgcgctcccgattccggaagtgcttgacattggggaattcagcgagagcctgacctattgcatctcccgccgtgcacagggtgtcacgttgcaagacctgcctgaaaccgaacctgcccgctgttctgcaaccggtcgcggaggccatggatgcgatcgctgcggccgatcttagccagacgagcgggttcggcccattcggaccgcaaggaatcctgcaggtcgatcgactctagaggatcagaaaattatcgccataaaagacagaataagtcatcagcggttgtttcatttcctatattttttttttatttttttattttttaataagggaaaatttaacgtctaaggatacagaagattgttagcacattaaagtaataaaggcttaagtagtaagtgccttagcatgttattgtatttcaaaggacataatctaaaataataacaatatcatttctcacaagttattcaattttcttttttttttctaataatatcaagaatgtattatttgtttgacataagtcaactaatttatttaatatgctggattaatcttgcagacatgtaaattaacaagttttagtcaaataacgttgaagtttcaatgaactcaaataatttctctttttttttatataaccataatctgatttatattttccgcagggatcaactgaagttatgacatttggattggatcacttataaccttggtcgccaaataatacaaaaatcagcgttataaaacaaagaaggtttttgttaagaaattaatcctctttcttgataagaaagttgaaccgaaattgcagatactgatatatgaaaataatacccacaattttgggaatagcgcaagcctcaatttaaacaataggtgaggacacatgataatgacctcaatgattgttagaagaaaagagcctcattacaaaatcgaaaaatgaatggttgggtacaagtttccaaaacatggtaaagtggactttgcgtatgagacgtaaatagaaaaaaacacttgttatatgttttctagaattattgttgtctctttatggttggatgatgcaaaatagtaatttcggttagttgctgtaaaacaccacgagacaaatagatatggatatttattaaatcaggaaaaacgtaactctcggctactggatggttcagtcacccaacgattactggggagagaaaacagggcaaaagcaaagcttaaaggaatccgattgtcattcggcaatgtgcagcgaaactaaaaaccggataatggacctgttaatcgaaacattgaagataaaggaagaggaatcctggcatatcatcaattgaataagttgaattaattatttcaatctcattctcactttctgacttatagtcgctttgttaaatcatatgtcgactctagaggatccccgggtaaaaggaatgtctcccttgccagtactgctagggtttttctttcaaactatggaagcccattcaagctgcatattacgattttgtttttcgcttttagaaagtggtttagatgagataatagaaaaattcttgatctccgacaacgagtacttttattttttttgctaatcactttactcaatattagctcgaaatcgtagaaacgtagacgggtgcgggataccgagtggtgtagttaagaatttttataaaccacgtggcccaaaaatatgaacccaaaacgtttatacatgagtatactttaagaaggctataccccttcgtgttagatgtagttttagctacccaacccgagtctatgagcttgacttcagatgtagaaggcattaaatcgttttgaatattaattaaaaaacgatgaaaattaaatatttaaaagcaatcatacgctgaaaatttagtgctgtggctaatccttcaacatggaaatgccataaaagtgactttgacaaaaaaaaaagtatatacaggtagtaaactcatctacttcattgactttgtttacagcatgtggaaggaggaatatttattgctaaatcgtagtttaacattcaataagtaatactattgaaattcgacaagattggccgcatggatgaaaaagaggcattttgctttgggagaattagttcaaattagaactgaaaaaaaaaactttacgaggcaaaaatgtcggattgagatcgtaaaagttcgctcgtcgtcttttgctttgtgattgttttcatggatacatcttgctggatatttaaattttagtactatgtataagatattctataaatgttttatcacccaaacctgttagcgccttcttaattctattcaatctggcttttgctctgagactacttcttggactttcactacttgttagttatacggaatttgtgtaattagaagtgaaataatcctttctattagtaatgcgagctcggcgcgccagatctgtttagcttgcctcgtccccgccgggtcacccggccagcgacatggaggcccagaataccctccttgacagtcttgacgtgcgcagctcaggggcatgatgtgactgtcgcccgtacatttagcccatacatccccatgtataatcatttgcatccatacattttgatggccgcacggcgcgaagcaaaaattacggctcctcgctgccgacctgcgagcagggaaacgctcccctcacagacgcgttgaattgtccccacgccgcgcccctgtagagaaatataaaaggttaggatttgccactgaggttcttctttcatatacttcctttttaaatcttgctaggatacagttctcacatcacatccgaacataaacaaccatgggtaccactcttgacgacacggcttaccggtaccgcaccagtgtcccaggggacgccgaggccatcgaggcactggatgggtccttcaccaccgacaccgtcttccgcgtcaccgccaccggggacggcttcaccctgcgggaggtgccggtggacccgcccctgaccaaggtgttccccgacgacgaatcggacgacgaatcggacgacggggaggacggcgacccggactctcggacgttcgtcgcgtacggggacgacggcgacctggcgggcttcgtggtcgtctcgtactccggctggaaccgccggctgaccgtcgaggacatcgaggtcgccccggagcaccgggggcacggggtcgggcgcgcgttgatggggctcgtgacggagttcgcccgcgagcggggtgccgggcacctctggctggaggtcaccaacgtcaacgcaccggcgatccacgcgtaccggcggatggggttcaccctctgcggcctggacaccgccctgtacgacggcaccgcctcggacggcgagcaggcgctctacatgagcatgccctgcccctaatcagtactgacaataaaaagattcttgttttcaagaacttgtcatttgtatagtttttttatattgtagttgttctattttaatcaaatgttagcgtgatttatatttttttcgcctcgacatcatctgcccagatgcgaagttaagtgcgcagaaagtaatatcatgcgtcaatcgtatgtgaatgctggtcgctatactgctgtcgattcgatactaacgccgccatccagaattcggtcaatacactacatggcgtgatttcatttgcgcgattgctgatccccatgtgtatcactggcaaactgtgatggacgacaccgtcggtgcgtccgtcgcgcaggctctcgatgagctgatgctttgggccgaggactgccccgaagtccggcacctcgtgcacgcggatttcggctccaacaatgtcctgacggacaatggccgcataacagcggtcattgactggagcgaggcgatgttcggggattcccaatacgaggtcgccaacatcttcttctggaggccgtggttggcttgtatggagcagcagacgcgctacttcgagcggaggcatccggagcttgcaggatcgccgcggctccgggcgtatatgctccgcattggtcttgaccaactctatcagagcttggttgacggcaatttcgatgatgcagcttgggcgcagggtcgatgcgacgcaatcgtccgatccggagccgggactgtcgggcgtacacaaatcgcccgcagaagcgcggccgtctggaccgatggctgtgtagaagtactcgccgatagtggaaaccgacgccccagcactcgtccgagggcaaaggaataatcagtactgacaataaaaagattcttgttttcaagaacttgtcatttgtatagtttttttatattgtagttgttctattttaatcaaatgttagcgtgatttatattttttttcgcctcgacatcatctgcccagatgcgaagttaagtgcgcagaaagtaatatcatgcgtcaatcgtatgtgaatgctggtcgctatactgctgtcgattcgatactaacgccgccatccagtttaaagcggccgcgaattcactggccgtcgttttacaacgtcgtgactgggaaaaccctggcgttacccaacttaatcgccttgcagcacatccccctttcgccagctggcgtaatagcgaagaggcccgcaccgatcgcccttcccaacagttgcgcagcctgaatggcgaatggcgcctgatgcggtattttctccttacgcatctgtgcggtatttcacaccgcatatatggtgcactctcagtacaatctgctctgatgccgcatagttaagccagccccgacacccgccaacacccgctgacgcgccctgacgggcttgtctgctcccggcatccgcttacagacaagctgtgaccgtctccgggagctgcatgtgtcagaggttttcaccgtcatcaccgaaacgcgcga

hphMX6

nmt41 prom

MCS

nmt term

ClonNat

beta-lactamase (AmpR)

**pINTH41PkN**

gacgaaagggcctcgtgatacgcctatttttataggttaatgtcatgataataatggtttcttagacgtcaggtggcacttttcggggaaatgtgcgcggaacccctatttgtttatttttctaaatacattcaaatatgtatccgctcatgagacaataaccctgataaatgcttcaataatattgaaaaaggaagagtatgagtattcaacatttccgtgtcgcccttattcccttttttgcggcattttgccttcctgtttttgctcacccagaaacgctggtgaaagtaaaagatgctgaagatcagttgggtgcacgagtgggttacatcgaactggatctcaacagcggtaagatccttgagagttttcgccccgaagaacgttttccaatgatgagcacttttaaagttctgctatgtggcgcggtattatcccgtattgacgccgggcaagagcaactcggtcgccgcatacactattctcagaatgacttggttgagtactcaccagtcacagaaaagcatcttacggatggcatgacagtaagagaattatgcagtgctgccataaccatgagtgataacactgcggccaacttacttctgacaacgatcggaggaccgaaggagctaaccgcttttttgcacaacatgggggatcatgtaactcgccttgatcgttgggaaccggagctgaatgaagccataccaaacgacgagcgtgacaccacgatgcctgtagcaatggcaacaacgttgcgcaaactattaactggcgaactacttactctagcttcccggcaacaattaatagactggatggaggcggataaagttgcaggaccacttctgcgctcggcccttccggctggctggtttattgctgataaatctggagccggtgagcgtgggtctcgcggtatcattgcagcactggggccagatggtaagccctcccgtatcgtagttatctacacgacggggagtcaggcaactatggatgaacgaaatagacagatcgctgagataggtgcctcactgattaagcattggtaactgtcagaccaagtttactcatatatactttagattgatttaaaacttcatttttaatttaaaaggatctaggtgaagatcctttttgataatctcatgaccaaaatcccttaacgtgagttttcgttccactgagcgtcagaccccgtagaaaagatcaaaggatcttcttgagatcctttttttctgcgcgtaatctgctgcttgcaaacaaaaaaaccaccgctaccagcggtggtttgtttgccggatcaagagctaccaactctttttccgaaggtaactggcttcagcagagcgcagataccaaatactgttcttctagtgtagccgtagttaggccaccacttcaagaactctgtagcaccgcctacatacctcgctctgctaatcctgttaccagtggctgctgccagtggcgataagtcgtgtcttaccgggttggactcaagacgatagttaccggataaggcgcagcggtcgggctgaacggggggttcgtgcacacagcccagcttggagcgaacgacctacaccgaactgagatacctacagcgtgagctatgagaaagcgccacgcttcccgaagggagaaaggcggacaggtatccggtaagcggcagggtcggaacaggagagcgcacgagggagcttccagggggaaacgcctggtatctttatagtcctgtcgggtttcgccacctctgacttgagcgtcgatttttgtgatgctcgtcaggggggcggagcctatggaaaaacgccagcaacgcggcctttttacggttcctggccttttgctggccttttgctcacatgttctttcctgcgttatcccctgattctgtggataaccgtattaccgcctttgagtgagctgataccgctcgccgcagccgaacgaccgagcgcagcgagtcagtgagcgaggaagcggaagagcgcccaatacgcaaaccgcctctccccgcgcgttggccgattcattaatgcagctggcacgacaggtttcccgactggaaagcgggcagtgagcgcaacgcaattaatgtgagttagctcactcattaggcaccccaggctttacactttatgcttccggctcgtatgttgtgtggaattgtgagcggataacaatttcacacaggaaacagctatgaccatgattacgccaagcttgcggccgcgttaattaaggcgcgccagatctgtttagcttgcctcgtccccgccgggtcacccggccagcgacatggaggcccagaataccctccttgacagtcttgacgtgcgcagctcaggggcatgatgtgactgtcgcccgtacatttagcccatacatccccatgtataatcatttgcatccatacattttgatggccgcacggcgcgaagcaaaaattacggctcctcgctgcggacctgcgagcagggaaacgctcccctcacagacgcgttgaattgtccccacgccgcgcccctgtagagaaatataaaaggttaggatttgccactgaggttcttctttcatatacttccttttaaaatcttgctaggatacagttctcacatcacatccgaacataaacaaccatgggtaaaaagcctgaactcaccgcgacgtctgtcgagaagtttctgatcgaaaagttcgacagcgtctccgacctgatgcggctctcggagggcgaagaatctcgtgctttcagcttcgatgtaggagggcgtggatatgtcctgcgggtaaatagctgcgccgatggtttctacaaagatcgttatgtttatcggcactttgcatcggccgcgctcccgattccggaagtgcttgacattggggaattcagcgagagcctgacctattgcatctcccgccgtgcacagggtgtcacgttgcaagacctgcctgaaaccgaacctgcccgctgttctgcaaccggtcgcggaggccatggatgcgatcgctgcggccgatcttagccagacgagcgggttcggcccattcggaccgcaaggaatcctgcaggtcgatcgactctagaggatcagaaaattatcgccataaaagacagaataagtcatcagcggttgtttcatttcctatattttttttttatttttttattttttaataagggaaaatttaacgtctaaggatacagaagattgttagcacattaaagtaataaaggcttaagtagtaagtgccttagcatgttattgtatttcaaaggacataatctaaaataataacaatatcatttctcacaagttattcaattttcttttttttttctaataatatcaagaatgtattatttgtttgacataagtcaactaatttatttaatatgctggattaatcttgcagacatgtaaattaacaagttttagtcaaataacgttgaagtttcaatgaactcaaataatttctctttttttttatataaccataatctgatttatattttccgcagggatcaactgaagttatgacatttggattggatcacttataaccttggtcgccaaataatacaaaaatcagcgttataaaacaaagaaggtttttgttaagaaattaatcctctttcttgataagaaagttgaaccgaaattgcagatactgatatatgaaaataatacccacaattttgggaatagcgcaagcctcaatttaaacaataggtgaggacacatgataatgacctcaatgattgttagaagaaaagagcctcattacaaaatcgaaaaatgaatggttgggtacaagtttccaaaacatggtaaagtggactttgcgtatgagacgtaaatagaaaaaaacacttgttatatgttttctagaattattgttgtctctttatggttggatgatgcaaaatagtaatttcggttagttgctgtaaaacaccacgagacaaatagatatggatatttattaaatcaggaaaaacgtaactctcggctactggatggttcagtcacccaacgattactggggagagaaaacagggcaaaagcaaagcttaaaggaatccgattgtcattcggcaatgtgcagcgaaactaaaaaccggataatggacctgttaatcgaaacattgaagataaaggaagaggaatcctggcatatcatcaattgaataagttgaattaattatttcaatctcattctcactttctgacttatagtcgctttgttaaatcatatcgagctcatgggtattcctaaccctttgcttggtcttgatggtattcctaaccctttgcttggtcttgattccatgggtattcctaaccctttgcttggtcttgatcatatgtcgacaggtaccagatctcgagggatccccgggtaaaaggaatgtctcccttgccagtactgctagggtttttctttcaaactatggaagcccattcaagctgcatattacgattttgtttttcgcttttagaaagtggtttagatgagataatagaaaaattcttgatctccgacaacgagtacttttattttttttgctaatcactttactcaatattagctcgaaatcgtagaaacgtagacgggtgcgggataccgagtggtgtagttaagaatttttataaaccacgtggcccaaaaatatgaacccaaaacgtttatacatgagtatactttaagaaggctataccccttcgtgttagatgtagttttagctacccaacccgagtctatgagcttgacttcagatgtagaaggcattaaatcgttttgaatattaattaaaaaacgatgaaaattaaatatttaaaagcaatcatacgctgaaaatttagtgctgtggctaatccttcaacatggaaatgccataaaagtgactttgacaaaaaaaaaagtatatacaggtagtaaactcatctacttcattgactttgtttacagcatgtggaaggaggaatatttattgctaaatcgtagtttaacattcaataagtaatactattgaaattcgacaagattggccgcatggatgaaaaagaggcattttgctttgggagaattagttcaaattagaactgaaaaaaaaaactttacgaggcaaaaatgtcggattgagatcgtaaaagttcgctcgtcgtcttttgctttgtgattgttttcatggatacatcttgctggatatttaaattttagtactatgtataagatattctataaatgttttatcacccaaacctgttagcgccttcttaattctattcaatctggcttttgctctgagactacttcttggactttcactacttgttagttatacggaatttgtgtaattagaagtgaaataatcctttctattagtaatgcgagctcggcgcgccagatctgtttagcttgcctcgtccccgccgggtcacccggccagcgacatggaggcccagaataccctccttgacagtcttgacgtgcgcagctcaggggcatgatgtgactgtcgcccgtacatttagcccatacatccccatgtataatcatttgcatccatacattttgatggccgcacggcgcgaagcaaaaattacggctcctcgctgccgacctgcgagcagggaaacgctcccctcacagacgcgttgaattgtccccacgccgcgcccctgtagagaaatataaaaggttaggatttgccactgaggttcttctttcatatacttcctttttaaatcttgctaggatacagttctcacatcacatccgaacataaacaaccatgggtaccactcttgacgacacggcttaccggtaccgcaccagtgtcccaggggacgccgaggccatcgaggcactggatgggtccttcaccaccgacaccgtcttccgcgtcaccgccaccggggacggcttcaccctgcgggaggtgccggtggacccgcccctgaccaaggtgttccccgacgacgaatcggacgacgaatcggacgacggggaggacggcgacccggactctcggacgttcgtcgcgtacggggacgacggcgacctggcgggcttcgtggtcgtctcgtactccggctggaaccgccggctgaccgtcgaggacatcgaggtcgccccggagcaccgggggcacggggtcgggcgcgcgttgatggggctcgtgacggagttcgcccgcgagcggggtgccgggcacctctggctggaggtcaccaacgtcaacgcaccggcgatccacgcgtaccggcggatggggttcaccctctgcggcctggacaccgccctgtacgacggcaccgcctcggacggcgagcaggcgctctacatgagcatgccctgcccctaatcagtactgacaataaaaagattcttgttttcaagaacttgtcatttgtatagtttttttatattgtagttgttctattttaatcaaatgttagcgtgatttatatttttttcgcctcgacatcatctgcccagatgcgaagttaagtgcgcagaaagtaatatcatgcgtcaatcgtatgtgaatgctggtcgctatactgctgtcgattcgatactaacgccgccatccagaattcggtcaatacactacatggcgtgatttcatttgcgcgattgctgatccccatgtgtatcactggcaaactgtgatggacgacaccgtcggtgcgtccgtcgcgcaggctctcgatgagctgatgctttgggccgaggactgccccgaagtccggcacctcgtgcacgcggatttcggctccaacaatgtcctgacggacaatggccgcataacagcggtcattgactggagcgaggcgatgttcggggattcccaatacgaggtcgccaacatcttcttctggaggccgtggttggcttgtatggagcagcagacgcgctacttcgagcggaggcatccggagcttgcaggatcgccgcggctccgggcgtatatgctccgcattggtcttgaccaactctatcagagcttggttgacggcaatttcgatgatgcagcttgggcgcagggtcgatgcgacgcaatcgtccgatccggagccgggactgtcgggcgtacacaaatcgcccgcagaagcgcggccgtctggaccgatggctgtgtagaagtactcgccgatagtggaaaccgacgccccagcactcgtccgagggcaaaggaataatcagtactgacaataaaaagattcttgttttcaagaacttgtcatttgtatagtttttttatattgtagttgttctattttaatcaaatgttagcgtgatttatattttttttcgcctcgacatcatctgcccagatgcgaagttaagtgcgcagaaagtaatatcatgcgtcaatcgtatgtgaatgctggtcgctatactgctgtcgattcgatactaacgccgccatccagtttaaagcggccgcgaattcactggccgtcgttttacaacgtcgtgactgggaaaaccctggcgttacccaacttaatcgccttgcagcacatccccctttcgccagctggcgtaatagcgaagaggcccgcaccgatcgcccttcccaacagttgcgcagcctgaatggcgaatggcgcctgatgcggtattttctccttacgcatctgtgcggtatttcacaccgcatatatggtgcactctcagtacaatctgctctgatgccgcatagttaagccagccccgacacccgccaacacccgctgacgcgccctgacgggcttgtctgctcccggcatccgcttacagacaagctgtgaccgtctccgggagctgcatgtgtcagaggttttcaccgtcatcaccgaaacgcgcga

hphMX6

nmt41 prom

MCS

PK tag

nmt term

ClonNat

beta-lactamase (AmpR)

**pINTH41PkC**

gacgaaagggcctcgtgatacgcctatttttataggttaatgtcatgataataatggtttcttagacgtcaggtggcacttttcggggaaatgtgcgcggaacccctatttgtttatttttctaaatacattcaaatatgtatccgctcatgagacaataaccctgataaatgcttcaataatattgaaaaaggaagagtatgagtattcaacatttccgtgtcgcccttattcccttttttgcggcattttgccttcctgtttttgctcacccagaaacgctggtgaaagtaaaagatgctgaagatcagttgggtgcacgagtgggttacatcgaactggatctcaacagcggtaagatccttgagagttttcgccccgaagaacgttttccaatgatgagcacttttaaagttctgctatgtggcgcggtattatcccgtattgacgccgggcaagagcaactcggtcgccgcatacactattctcagaatgacttggttgagtactcaccagtcacagaaaagcatcttacggatggcatgacagtaagagaattatgcagtgctgccataaccatgagtgataacactgcggccaacttacttctgacaacgatcggaggaccgaaggagctaaccgcttttttgcacaacatgggggatcatgtaactcgccttgatcgttgggaaccggagctgaatgaagccataccaaacgacgagcgtgacaccacgatgcctgtagcaatggcaacaacgttgcgcaaactattaactggcgaactacttactctagcttcccggcaacaattaatagactggatggaggcggataaagttgcaggaccacttctgcgctcggcccttccggctggctggtttattgctgataaatctggagccggtgagcgtgggtctcgcggtatcattgcagcactggggccagatggtaagccctcccgtatcgtagttatctacacgacggggagtcaggcaactatggatgaacgaaatagacagatcgctgagataggtgcctcactgattaagcattggtaactgtcagaccaagtttactcatatatactttagattgatttaaaacttcatttttaatttaaaaggatctaggtgaagatcctttttgataatctcatgaccaaaatcccttaacgtgagttttcgttccactgagcgtcagaccccgtagaaaagatcaaaggatcttcttgagatcctttttttctgcgcgtaatctgctgcttgcaaacaaaaaaaccaccgctaccagcggtggtttgtttgccggatcaagagctaccaactctttttccgaaggtaactggcttcagcagagcgcagataccaaatactgttcttctagtgtagccgtagttaggccaccacttcaagaactctgtagcaccgcctacatacctcgctctgctaatcctgttaccagtggctgctgccagtggcgataagtcgtgtcttaccgggttggactcaagacgatagttaccggataaggcgcagcggtcgggctgaacggggggttcgtgcacacagcccagcttggagcgaacgacctacaccgaactgagatacctacagcgtgagctatgagaaagcgccacgcttcccgaagggagaaaggcggacaggtatccggtaagcggcagggtcggaacaggagagcgcacgagggagcttccagggggaaacgcctggtatctttatagtcctgtcgggtttcgccacctctgacttgagcgtcgatttttgtgatgctcgtcaggggggcggagcctatggaaaaacgccagcaacgcggcctttttacggttcctggccttttgctggccttttgctcacatgttctttcctgcgttatcccctgattctgtggataaccgtattaccgcctttgagtgagctgataccgctcgccgcagccgaacgaccgagcgcagcgagtcagtgagcgaggaagcggaagagcgcccaatacgcaaaccgcctctccccgcgcgttggccgattcattaatgcagctggcacgacaggtttcccgactggaaagcgggcagtgagcgcaacgcaattaatgtgagttagctcactcattaggcaccccaggctttacactttatgcttccggctcgtatgttgtgtggaattgtgagcggataacaatttcacacaggaaacagctatgaccatgattacgccaagcttgcggccgcgttaattaaggcgcgccagatctgtttagcttgcctcgtccccgccgggtcacccggccagcgacatggaggcccagaataccctccttgacagtcttgacgtgcgcagctcaggggcatgatgtgactgtcgcccgtacatttagcccatacatccccatgtataatcatttgcatccatacattttgatggccgcacggcgcgaagcaaaaattacggctcctcgctgcggacctgcgagcagggaaacgctcccctcacagacgcgttgaattgtccccacgccgcgcccctgtagagaaatataaaaggttaggatttgccactgaggttcttctttcatatacttccttttaaaatcttgctaggatacagttctcacatcacatccgaacataaacaaccatgggtaaaaagcctgaactcaccgcgacgtctgtcgagaagtttctgatcgaaaagttcgacagcgtctccgacctgatgcggctctcggagggcgaagaatctcgtgctttcagcttcgatgtaggagggcgtggatatgtcctgcgggtaaatagctgcgccgatggtttctacaaagatcgttatgtttatcggcactttgcatcggccgcgctcccgattccggaagtgcttgacattggggaattcagcgagagcctgacctattgcatctcccgccgtgcacagggtgtcacgttgcaagacctgcctgaaaccgaacctgcccgctgttctgcaaccggtcgcggaggccatggatgcgatcgctgcggccgatcttagccagacgagcgggttcggcccattcggaccgcaaggaatcctgcaggtcgatcgactctagaggatcagaaaattatcgccataaaagacagaataagtcatcagcggttgtttcatttcctatattttttttttatttttttattttttaataagggaaaatttaacgtctaaggatacagaagattgttagcacattaaagtaataaaggcttaagtagtaagtgccttagcatgttattgtatttcaaaggacataatctaaaataataacaatatcatttctcacaagttattcaattttcttttttttttctaataatatcaagaatgtattatttgtttgacataagtcaactaatttatttaatatgctggattaatcttgcagacatgtaaattaacaagttttagtcaaataacgttgaagtttcaatgaactcaaataatttctctttttttttatataaccataatctgatttatattttccgcagggatcaactgaagttatgacatttggattggatcacttataaccttggtcgccaaataatacaaaaatcagcgttataaaacaaagaaggtttttgttaagaaattaatcctctttcttgataagaaagttgaaccgaaattgcagatactgatatatgaaaataatacccacaattttgggaatagcgcaagcctcaatttaaacaataggtgaggacacatgataatgacctcaatgattgttagaagaaaagagcctcattacaaaatcgaaaaatgaatggttgggtacaagtttccaaaacatggtaaagtggactttgcgtatgagacgtaaatagaaaaaaacacttgttatatgttttctagaattattgttgtctctttatggttggatgatgcaaaatagtaatttcggttagttgctgtaaaacaccacgagacaaatagatatggatatttattaaatcaggaaaaacgtaactctcggctactggatggttcagtcacccaacgattactggggagagaaaacagggcaaaagcaaagcttaaaggaatccgattgtcattcggcaatgtgcagcgaaactaaaaaccggataatggacctgttaatcgaaacattgaagataaaggaagaggaatcctggcatatcatcaattgaataagttgaattaattatttcaatctcattctcactttctgacttatagtcgctttgttaaatcatatgtcgacatggtaccagatctctcgaggatccccgggtatgggtattcctaaccctttgcttggtcttgatggtattcctaaccctttgcttggtcttgattccatgggtattcctaaccctttgcttggtcttgatcattaaggatcgggtaaaaggaatgtctcccttgccagtactgctagggtttttctttcaaactatggaagcccattcaagctgcatattacgattttgtttttcgcttttagaaagtggtttagatgagataatagaaaaattcttgatctccgacaacgagtacttttattttttttgctaatcactttactcaatattagctcgaaatcgtagaaacgtagacgggtgcgggataccgagtggtgtagttaagaatttttataaaccacgtggcccaaaaatatgaacccaaaacgtttatacatgagtatactttaagaaggctataccccttcgtgttagatgtagttttagctacccaacccgagtctatgagcttgacttcagatgtagaaggcattaaatcgttttgaatattaattaaaaaacgatgaaaattaaatatttaaaagcaatcatacgctgaaaatttagtgctgtggctaatccttcaacatggaaatgccataaaagtgactttgacaaaaaaaaaagtatatacaggtagtaaactcatctacttcattgactttgtttacagcatgtggaaggaggaatatttattgctaaatcgtagtttaacattcaataagtaatactattgaaattcgacaagattggccgcatggatgaaaaagaggcattttgctttgggagaattagttcaaattagaactgaaaaaaaaaactttacgaggcaaaaatgtcggattgagatcgtaaaagttcgctcgtcgtcttttgctttgtgattgttttcatggatacatcttgctggatatttaaattttagtactatgtataagatattctataaatgttttatcacccaaacctgttagcgccttcttaattctattcaatctggcttttgctctgagactacttcttggactttcactacttgttagttatacggaatttgtgtaattagaagtgaaataatcctttctattagtaatgcgagctcggcgcgccagatctgtttagcttgcctcgtccccgccgggtcacccggccagcgacatggaggcccagaataccctccttgacagtcttgacgtgcgcagctcaggggcatgatgtgactgtcgcccgtacatttagcccatacatccccatgtataatcatttgcatccatacattttgatggccgcacggcgcgaagcaaaaattacggctcctcgctgccgacctgcgagcagggaaacgctcccctcacagacgcgttgaattgtccccacgccgcgcccctgtagagaaatataaaaggttaggatttgccactgaggttcttctttcatatacttcctttttaaatcttgctaggatacagttctcacatcacatccgaacataaacaaccatgggtaccactcttgacgacacggcttaccggtaccgcaccagtgtcccaggggacgccgaggccatcgaggcactggatgggtccttcaccaccgacaccgtcttccgcgtcaccgccaccggggacggcttcaccctgcgggaggtgccggtggacccgcccctgaccaaggtgttccccgacgacgaatcggacgacgaatcggacgacggggaggacggcgacccggactctcggacgttcgtcgcgtacggggacgacggcgacctggcgggcttcgtggtcgtctcgtactccggctggaaccgccggctgaccgtcgaggacatcgaggtcgccccggagcaccgggggcacggggtcgggcgcgcgttgatggggctcgtgacggagttcgcccgcgagcggggtgccgggcacctctggctggaggtcaccaacgtcaacgcaccggcgatccacgcgtaccggcggatggggttcaccctctgcggcctggacaccgccctgtacgacggcaccgcctcggacggcgagcaggcgctctacatgagcatgccctgcccctaatcagtactgacaataaaaagattcttgttttcaagaacttgtcatttgtatagtttttttatattgtagttgttctattttaatcaaatgttagcgtgatttatatttttttcgcctcgacatcatctgcccagatgcgaagttaagtgcgcagaaagtaatatcatgcgtcaatcgtatgtgaatgctggtcgctatactgctgtcgattcgatactaacgccgccatccagaattcggtcaatacactacatggcgtgatttcatttgcgcgattgctgatccccatgtgtatcactggcaaactgtgatggacgacaccgtcggtgcgtccgtcgcgcaggctctcgatgagctgatgctttgggccgaggactgccccgaagtccggcacctcgtgcacgcggatttcggctccaacaatgtcctgacggacaatggccgcataacagcggtcattgactggagcgaggcgatgttcggggattcccaatacgaggtcgccaacatcttcttctggaggccgtggttggcttgtatggagcagcagacgcgctacttcgagcggaggcatccggagcttgcaggatcgccgcggctccgggcgtatatgctccgcattggtcttgaccaactctatcagagcttggttgacggcaatttcgatgatgcagcttgggcgcagggtcgatgcgacgcaatcgtccgaccgggagctgcatgtgtcagaggttttcaccgtcatcaccgaaacgcgcga

hphMX6

nmt41 prom

MCS

PK tag

nmt term

ClonNat

beta-lactamase (AmpR)

**pINTH41HAN**

gacgaaagggcctcgtgatacgcctatttttataggttaatgtcatgataataatggtttcttagacgtcaggtggcacttttcggggaaatgtgcgcggaacccctatttgtttatttttctaaatacattcaaatatgtatccgctcatgagacaataaccctgataaatgcttcaataatattgaaaaaggaagagtatgagtattcaacatttccgtgtcgcccttattcccttttttgcggcattttgccttcctgtttttgctcacccagaaacgctggtgaaagtaaaagatgctgaagatcagttgggtgcacgagtgggttacatcgaactggatctcaacagcggtaagatccttgagagttttcgccccgaagaacgttttccaatgatgagcacttttaaagttctgctatgtggcgcggtattatcccgtattgacgccgggcaagagcaactcggtcgccgcatacactattctcagaatgacttggttgagtactcaccagtcacagaaaagcatcttacggatggcatgacagtaagagaattatgcagtgctgccataaccatgagtgataacactgcggccaacttacttctgacaacgatcggaggaccgaaggagctaaccgcttttttgcacaacatgggggatcatgtaactcgccttgatcgttgggaaccggagctgaatgaagccataccaaacgacgagcgtgacaccacgatgcctgtagcaatggcaacaacgttgcgcaaactattaactggcgaactacttactctagcttcccggcaacaattaatagactggatggaggcggataaagttgcaggaccacttctgcgctcggcccttccggctggctggtttattgctgataaatctggagccggtgagcgtgggtctcgcggtatcattgcagcactggggccagatggtaagccctcccgtatcgtagttatctacacgacggggagtcaggcaactatggatgaacgaaatagacagatcgctgagataggtgcctcactgattaagcattggtaactgtcagaccaagtttactcatatatactttagattgatttaaaacttcatttttaatttaaaaggatctaggtgaagatcctttttgataatctcatgaccaaaatcccttaacgtgagttttcgttccactgagcgtcagaccccgtagaaaagatcaaaggatcttcttgagatcctttttttctgcgcgtaatctgctgcttgcaaacaaaaaaaccaccgctaccagcggtggtttgtttgccggatcaagagctaccaactctttttccgaaggtaactggcttcagcagagcgcagataccaaatactgttcttctagtgtagccgtagttaggccaccacttcaagaactctgtagcaccgcctacatacctcgctctgctaatcctgttaccagtggctgctgccagtggcgataagtcgtgtcttaccgggttggactcaagacgatagttaccggataaggcgcagcggtcgggctgaacggggggttcgtgcacacagcccagcttggagcgaacgacctacaccgaactgagatacctacagcgtgagctatgagaaagcgccacgcttcccgaagggagaaaggcggacaggtatccggtaagcggcagggtcggaacaggagagcgcacgagggagcttccagggggaaacgcctggtatctttatagtcctgtcgggtttcgccacctctgacttgagcgtcgatttttgtgatgctcgtcaggggggcggagcctatggaaaaacgccagcaacgcggcctttttacggttcctggccttttgctggccttttgctcacatgttctttcctgcgttatcccctgattctgtggataaccgtattaccgcctttgagtgagctgataccgctcgccgcagccgaacgaccgagcgcagcgagtcagtgagcgaggaagcggaagagcgcccaatacgcaaaccgcctctccccgcgcgttggccgattcattaatgcagctggcacgacaggtttcccgactggaaagcgggcagtgagcgcaacgcaattaatgtgagttagctcactcattaggcaccccaggctttacactttatgcttccggctcgtatgttgtgtggaattgtgagcggataacaatttcacacaggaaacagctatgaccatgattacgccaagcttgcggccgcgttaattaaggcgcgccagatctgtttagcttgcctcgtccccgccgggtcacccggccagcgacatggaggcccagaataccctccttgacagtcttgacgtgcgcagctcaggggcatgatgtgactgtcgcccgtacatttagcccatacatccccatgtataatcatttgcatccatacattttgatggccgcacggcgcgaagcaaaaattacggctcctcgctgcggacctgcgagcagggaaacgctcccctcacagacgcgttgaattgtccccacgccgcgcccctgtagagaaatataaaaggttaggatttgccactgaggttcttctttcatatacttccttttaaaatcttgctaggatacagttctcacatcacatccgaacataaacaaccatgggtaaaaagcctgaactcaccgcgacgtctgtcgagaagtttctgatcgaaaagttcgacagcgtctccgacctgatgcggctctcggagggcgaagaatctcgtgctttcagcttcgatgtaggagggcgtggatatgtcctgcgggtaaatagctgcgccgatggtttctacaaagatcgttatgtttatcggcactttgcatcggccgcgctcccgattccggaagtgcttgacattggggaattcagcgagagcctgacctattgcatctcccgccgtgcacagggtgtcacgttgcaagacctgcctgaaaccgaacctgcccgctgttctgcaaccggtcgcggaggccatggatgcgatcgctgcggccgatcttagccagacgagcgggttcggcccattcggaccgcaaggaatcctgcaggtcgatcgactctagaggatcagaaaattatcgccataaaagacagaataagtcatcagcggttgtttcatttcctatattttttttttatttttttattttttaataagggaaaatttaacgtctaaggatacagaagattgttagcacattaaagtaataaaggcttaagtagtaagtgccttagcatgttattgtatttcaaaggacataatctaaaataataacaatatcatttctcacaagttattcaattttcttttttttttctaataatatcaagaatgtattatttgtttgacataagtcaactaatttatttaatatgctggattaatcttgcagacatgtaaattaacaagttttagtcaaataacgttgaagtttcaatgaactcaaataatttctctttttttttatataaccataatctgatttatattttccgcagggatcaactgaagttatgacatttggattggatcacttataaccttggtcgccaaataatacaaaaatcagcgttataaaacaaagaaggtttttgttaagaaattaatcctctttcttgataagaaagttgaaccgaaattgcagatactgatatatgaaaataatacccacaattttgggaatagcgcaagcctcaatttaaacaataggtgaggacacatgataatgacctcaatgattgttagaagaaaagagcctcattacaaaatcgaaaaatgaatggttgggtacaagtttccaaaacatggtaaagtggactttgcgtatgagacgtaaatagaaaaaaacacttgttatatgttttctagaattattgttgtctctttatggttggatgatgcaaaatagtaatttcggttagttgctgtaaaacaccacgagacaaatagatatggatatttattaaatcaggaaaaacgtaactctcggctactggatggttcagtcacccaacgattactggggagagaaaacagggcaaaagcaaagcttaaaggaatccgattgtcattcggcaatgtgcagcgaaactaaaaaccggataatggacctgttaatcgaaacattgaagataaaggaagaggaatcctggcatatcatcaattgaataagttgaattaattatttcaatctcattctcactttctgacttatagtcgctttgttaaatcccatggcatacccgtacgatgttcctgactatgcgggctatccctatgacgtcccggactatgccatgggctacccttacgacgttccagattacgctcatatgtcgactctagaggatccccgggtaaaaggaatgtctcccttgccagtactgctagggtttttctttcaaactatggaagcccattcaagctgcatattacgattttgtttttcgcttttagaaagtggtttagatgagataatagaaaaattcttgatctccgacaacgagtacttttattttttttgctaatcactttactcaatattagctcgaaatcgtagaaacgtagacgggtgcgggataccgagtggtgtagttaagaatttttataaaccacgtggcccaaaaatatgaacccaaaacgtttatacatgagtatactttaagaaggctataccccttcgtgttagatgtagttttagctacccaacccgagtctatgagcttgacttcagatgtagaaggcattaaatcgttttgaatattaattaaaaaacgatgaaaattaaatatttaaaagcaatcatacgctgaaaatttagtgctgtggctaatccttcaacatggaaatgccataaaagtgactttgacaaaaaaaaaagtatatacaggtagtaaactcatctacttcattgactttgtttacagcatgtggaaggaggaatatttattgctaaatcgtagtttaacattcaataagtaatactattgaaattcgacaagattggccgcatggatgaaaaagaggcattttgctttgggagaattagttcaaattagaactgaaaaaaaaaactttacgaggcaaaaatgtcggattgagatcgtaaaagttcgctcgtcgtcttttgctttgtgattgttttcatggatacatcttgctggatatttaaattttagtactatgtataagatattctataaatgttttatcacccaaacctgttagcgccttcttaattctattcaatctggcttttgctctgagactacttcttggactttcactacttgttagttatacggaatttgtgtaattagaagtgaaataatcctttctattagtaatgcgagctcggcgcgccagatctgtttagcttgcctcgtccccgccgggtcacccggccagcgacatggaggcccagaataccctccttgacagtcttgacgtgcgcagctcaggggcatgatgtgactgtcgcccgtacatttagcccatacatccccatgtataatcatttgcatccatacattttgatggccgcacggcgcgaagcaaaaattacggctcctcgctgccgacctgcgagcagggaaacgctcccctcacagacgcgttgaattgtccccacgccgcgcccctgtagagaaatataaaaggttaggatttgccactgaggttcttctttcatatacttcctttttaaatcttgctaggatacagttctcacatcacatccgaacataaacaaccatgggtaccactcttgacgacacggcttaccggtaccgcaccagtgtcccaggggacgccgaggccatcgaggcactggatgggtccttcaccaccgacaccgtcttccgcgtcaccgccaccggggacggcttcaccctgcgggaggtgccggtggacccgcccctgaccaaggtgttccccgacgacgaatcggacgacgaatcggacgacggggaggacggcgacccggactctcggacgttcgtcgcgtacggggacgacggcgacctggcgggcttcgtggtcgtctcgtactccggctggaaccgccggctgaccgtcgaggacatcgaggtcgccccggagcaccgggggcacggggtcgggcgcgcgttgatggggctcgtgacggagttcgcccgcgagcggggtgccgggcacctctggctggaggtcaccaacgtcaacgcaccggcgatccacgcgtaccggcggatggggttcaccctctgcggcctggacaccgccctgtacgacggcaccgcctcggacggcgagcaggcgctctacatgagcatgccctgcccctaatcagtactgacaataaaaagattcttgttttcaagaacttgtcatttgtatagtttttttatattgtagttgttctattttaatcaaatgttagcgtgatttatatttttttcgcctcgacatcatctgcccagatgcgaagttaagtgcgcagaaagtaatatcatgcgtcaatcgtatgtgaatgctggtcgctatactgctgtcgattcgatactaacgccgccatccagaattcggtcaatacactacatggcgtgatttcatttgcgcgattgctgatccccatgtgtatcactggcaaactgtgatggacgacaccgtcggtgcgtccgtcgcgcaggctctcgatgagctgatgctttgggccgaggactgccccgaagtccggcacctcgtgcacgcggatttcggctccaacaatgtcctgacggacaatggccgcataacagcggtcattgactggagcgaggcgatgttcggggattcccaatacgaggtcgccaacatcttcttctggaggccgtggttggcttgtatggagcagcagacgcgctacttcgagcggaggcatccggagcttgcaggatcgccgcggctccgggcgtatatgctccgcattggtcttgaccaactctatcagagcttggttgacggcaatttcgatgatgcagcttgggcgcagggtcgatgcgacgcaatcgtccgatccggagccgggactgtcgggcgtacacaaatcgcccgcagaagcgcggccgtctggaccgatggctgtgtagaagtactcgccgatagtggaaaccgacgccccagcactcgtccgagggcaaaggaataatcagtactgacaataaaaagattcttgttttcaagaacttgtcatttgtatagtttttttatattgtagttgttctattttaatcaaatgttagcgtgatttatattttttttcgcctcgacatcatctgcccagatgcgaagttaagtgcgcagaaagtaatatcatgcgtcaatcgtatgtgaatgctggtcgctatactgctgtcgattcgatactaacgccgccatccagtttaaagcggccgcgaattcactggccgtcgttttacaacgtcgtgactgggaaaaccctggcgttacccaacttaatcgccttgcagcacatccccctttcgccagctggcgtaatagcgaagaggcccgcaccgatcgcccttcccaacagttgcgcagcctgaatggcgaatggcgcctgatgcggtattttctccttacgcatctgtgcggtatttcacaccgcatatatggtgcactctcagtacaatctgctctgatgccgcatagttaagccagccccgacacccgccaacacccgctgacgcgccctgacgggcttgtctgctcccggcatccgcttacagacaagctgtgaccgtctccgggagctgcatgtgtcagaggttttcaccgtcatcaccgaaacgcgcga

hphMX6

nmt41 prom

MCS

HA tag

nmt term

ClonNat

beta-lactamase (AmpR)

**pINTH41HMN**

gacgaaagggcctcgtgatacgcctatttttataggttaatgtcatgataataatggtttcttagacgtcaggtggcacttttcggggaaatgtgcgcggaacccctatttgtttatttttctaaatacattcaaatatgtatccgctcatgagacaataaccctgataaatgcttcaataatattgaaaaaggaagagtatgagtattcaacatttccgtgtcgcccttattcccttttttgcggcattttgccttcctgtttttgctcacccagaaacgctggtgaaagtaaaagatgctgaagatcagttgggtgcacgagtgggttacatcgaactggatctcaacagcggtaagatccttgagagttttcgccccgaagaacgttttccaatgatgagcacttttaaagttctgctatgtggcgcggtattatcccgtattgacgccgggcaagagcaactcggtcgccgcatacactattctcagaatgacttggttgagtactcaccagtcacagaaaagcatcttacggatggcatgacagtaagagaattatgcagtgctgccataaccatgagtgataacactgcggccaacttacttctgacaacgatcggaggaccgaaggagctaaccgcttttttgcacaacatgggggatcatgtaactcgccttgatcgttgggaaccggagctgaatgaagccataccaaacgacgagcgtgacaccacgatgcctgtagcaatggcaacaacgttgcgcaaactattaactggcgaactacttactctagcttcccggcaacaattaatagactggatggaggcggataaagttgcaggaccacttctgcgctcggcccttccggctggctggtttattgctgataaatctggagccggtgagcgtgggtctcgcggtatcattgcagcactggggccagatggtaagccctcccgtatcgtagttatctacacgacggggagtcaggcaactatggatgaacgaaatagacagatcgctgagataggtgcctcactgattaagcattggtaactgtcagaccaagtttactcatatatactttagattgatttaaaacttcatttttaatttaaaaggatctaggtgaagatcctttttgataatctcatgaccaaaatcccttaacgtgagttttcgttccactgagcgtcagaccccgtagaaaagatcaaaggatcttcttgagatcctttttttctgcgcgtaatctgctgcttgcaaacaaaaaaaccaccgctaccagcggtggtttgtttgccggatcaagagctaccaactctttttccgaaggtaactggcttcagcagagcgcagataccaaatactgttcttctagtgtagccgtagttaggccaccacttcaagaactctgtagcaccgcctacatacctcgctctgctaatcctgttaccagtggctgctgccagtggcgataagtcgtgtcttaccgggttggactcaagacgatagttaccggataaggcgcagcggtcgggctgaacggggggttcgtgcacacagcccagcttggagcgaacgacctacaccgaactgagatacctacagcgtgagctatgagaaagcgccacgcttcccgaagggagaaaggcggacaggtatccggtaagcggcagggtcggaacaggagagcgcacgagggagcttccagggggaaacgcctggtatctttatagtcctgtcgggtttcgccacctctgacttgagcgtcgatttttgtgatgctcgtcaggggggcggagcctatggaaaaacgccagcaacgcggcctttttacggttcctggccttttgctggccttttgctcacatgttctttcctgcgttatcccctgattctgtggataaccgtattaccgcctttgagtgagctgataccgctcgccgcagccgaacgaccgagcgcagcgagtcagtgagcgaggaagcggaagagcgcccaatacgcaaaccgcctctccccgcgcgttggccgattcattaatgcagctggcacgacaggtttcccgactggaaagcgggcagtgagcgcaacgcaattaatgtgagttagctcactcattaggcaccccaggctttacactttatgcttccggctcgtatgttgtgtggaattgtgagcggataacaatttcacacaggaaacagctatgaccatgattacgccaagcttgcggccgcgttaattaaggcgcgccagatctgtttagcttgcctcgtccccgccgggtcacccggccagcgacatggaggcccagaataccctccttgacagtcttgacgtgcgcagctcaggggcatgatgtgactgtcgcccgtacatttagcccatacatccccatgtataatcatttgcatccatacattttgatggccgcacggcgcgaagcaaaaattacggctcctcgctgcggacctgcgagcagggaaacgctcccctcacagacgcgttgaattgtccccacgccgcgcccctgtagagaaatataaaaggttaggatttgccactgaggttcttctttcatatacttccttttaaaatcttgctaggatacagttctcacatcacatccgaacataaacaaccatgggtaaaaagcctgaactcaccgcgacgtctgtcgagaagtttctgatcgaaaagttcgacagcgtctccgacctgatgcggctctcggagggcgaagaatctcgtgctttcagcttcgatgtaggagggcgtggatatgtcctgcgggtaaatagctgcgccgatggtttctacaaagatcgttatgtttatcggcactttgcatcggccgcgctcccgattccggaagtgcttgacattggggaattcagcgagagcctgacctattgcatctcccgccgtgcacagggtgtcacgttgcaagacctgcctgaaaccgaacctgcccgctgttctgcaaccggtcgcggaggccatggatgcgatcgctgcggccgatcttagccagacgagcgggttcggcccattcggaccgcaaggaatcctgcaggtcgatcgactctagaggatcagaaaattatcgccataaaagacagaataagtcatcagcggttgtttcatttcctatattttttttttatttttttattttttaataagggaaaatttaacgtctaaggatacagaagattgttagcacattaaagtaataaaggcttaagtagtaagtgccttagcatgttattgtatttcaaaggacataatctaaaataataacaatatcatttctcacaagttattcaattttcttttttttttctaataatatcaagaatgtattatttgtttgacataagtcaactaatttatttaatatgctggattaatcttgcagacatgtaaattaacaagttttagtcaaataacgttgaagtttcaatgaactcaaataatttctctttttttttatataaccataatctgatttatattttccgcagggatcaactgaagttatgacatttggattggatcacttataaccttggtcgccaaataatacaaaaatcagcgttataaaacaaagaaggtttttgttaagaaattaatcctctttcttgataagaaagttgaaccgaaattgcagatactgatatatgaaaataatacccacaattttgggaatagcgcaagcctcaatttaaacaataggtgaggacacatgataatgacctcaatgattgttagaagaaaagagcctcattacaaaatcgaaaaatgaatggttgggtacaagtttccaaaacatggtaaagtggactttgcgtatgagacgtaaatagaaaaaaacacttgttatatgttttctagaattattgttgtctctttatggttggatgatgcaaaatagtaatttcggttagttgctgtaaaacaccacgagacaaatagatatggatatttattaaatcaggaaaaacgtaactctcggctactggatggttcagtcacccaacgattactggggagagaaaacagggcaaaagcaaagcttaaaggaatccgattgtcattcggcaatgtgcagcgaaactaaaaaccggataatggacctgttaatcgaaacattgaagataaaggaagaggaatcctggcatatcatcaattgaataagttgaattaattatttcaatctcattctcactttctgacttatagtcgctttgttaaatcccatgggtagcagccaccatcatcaccatcatgctgaggagcaaaagttaatttctgaagaagatttgtccatggctgaagaacaaaaattgatcagcgaggaggacttacatatgtcgactctagaggatccccgggtaaaaggaatgtctcccttgccagtactgctagggtttttctttcaaactatggaagcccattcaagctgcatattacgattttgtttttcgcttttagaaagtggtttagatgagataatagaaaaattcttgatctccgacaacgagtacttttattttttttgctaatcactttactcaatattagctcgaaatcgtagaaacgtagacgggtgcgggataccgagtggtgtagttaagaatttttataaaccacgtggcccaaaaatatgaacccaaaacgtttatacatgagtatactttaagaaggctataccccttcgtgttagatgtagttttagctacccaacccgagtctatgagcttgacttcagatgtagaaggcattaaatcgttttgaatattaattaaaaaacgatgaaaattaaatatttaaaagcaatcatacgctgaaaatttagtgctgtggctaatccttcaacatggaaatgccataaaagtgactttgacaaaaaaaaaagtatatacaggtagtaaactcatctacttcattgactttgtttacagcatgtggaaggaggaatatttattgctaaatcgtagtttaacattcaataagtaatactattgaaattcgacaagattggccgcatggatgaaaaagaggcattttgctttgggagaattagttcaaattagaactgaaaaaaaaaactttacgaggcaaaaatgtcggattgagatcgtaaaagttcgctcgtcgtcttttgctttgtgattgttttcatggatacatcttgctggatatttaaattttagtactatgtataagatattctataaatgttttatcacccaaacctgttagcgccttcttaattctattcaatctggcttttgctctgagactacttcttggactttcactacttgttagttatacggaatttgtgtaattagaagtgaaataatcctttctattagtaatgcgagctcggcgcgccagatctgtttagcttgcctcgtccccgccgggtcacccggccagcgacatggaggcccagaataccctccttgacagtcttgacgtgcgcagctcaggggcatgatgtgactgtcgcccgtacatttagcccatacatccccatgtataatcatttgcatccatacattttgatggccgcacggcgcgaagcaaaaattacggctcctcgctgccgacctgcgagcagggaaacgctcccctcacagacgcgttgaattgtccccacgccgcgcccctgtagagaaatataaaaggttaggatttgccactgaggttcttctttcatatacttcctttttaaatcttgctaggatacagttctcacatcacatccgaacataaacaaccatgggtaccactcttgacgacacggcttaccggtaccgcaccagtgtcccaggggacgccgaggccatcgaggcactggatgggtccttcaccaccgacaccgtcttccgcgtcaccgccaccggggacggcttcaccctgcgggaggtgccggtggacccgcccctgaccaaggtgttccccgacgacgaatcggacgacgaatcggacgacggggaggacggcgacccggactctcggacgttcgtcgcgtacggggacgacggcgacctggcgggcttcgtggtcgtctcgtactccggctggaaccgccggctgaccgtcgaggacatcgaggtcgccccggagcaccgggggcacggggtcgggcgcgcgttgatggggctcgtgacggagttcgcccgcgagcggggtgccgggcacctctggctggaggtcaccaacgtcaacgcaccggcgatccacgcgtaccggcggatggggttcaccctctgcggcctggacaccgccctgtacgacggcaccgcctcggacggcgagcaggcgctctacatgagcatgccctgcccctaatcagtactgacaataaaaagattcttgttttcaagaacttgtcatttgtatagtttttttatattgtagttgttctattttaatcaaatgttagcgtgatttatatttttttcgcctcgacatcatctgcccagatgcgaagttaagtgcgcagaaagtaatatcatgcgtcaatcgtatgtgaatgctggtcgctatactgctgtcgattcgatactaacgccgccatccagaattcggtcaatacactacatggcgtgatttcatttgcgcgattgctgatccccatgtgtatcactggcaaactgtgatggacgacaccgtcggtgcgtccgtcgcgcaggctctcgatgagctgatgctttgggccgaggactgccccgaagtccggcacctcgtgcacgcggatttcggctccaacaatgtcctgacggacaatggccgcataacagcggtcattgactggagcgaggcgatgttcggggattcccaatacgaggtcgccaacatcttcttctggaggccgtggttggcttgtatggagcagcagacgcgctacttcgagcggaggcatccggagcttgcaggatcgccgcggctccgggcgtatatgctccgcattggtcttgaccaactctatcagagcttggttgacggcaatttcgatgatgcagcttgggcgcagggtcgatgcgacgcaatcgtccgatccggagccgggactgtcgggcgtacacaaatcgcccgcagaagcgcggccgtctggaccgatggctgtgtagaagtactcgccgatagtggaaaccgacgccccagcactcgtccgagggcaaaggaataatcagtactgacaataaaaagattcttgttttcaagaacttgtcatttgtatagtttttttatattgtagttgttctattttaatcaaatgttagcgtgatttatattttttttcgcctcgacatcatctgcccagatgcgaagttaagtgcgcagaaagtaatatcatgcgtcaatcgtatgtgaatgctggtcgctatactgctgtcgattcgatactaacgccgccatccagtttaaagcggccgcgaattcactggccgtcgttttacaacgtcgtgactgggaaaaccctggcgttacccaacttaatcgccttgcagcacatccccctttcgccagctggcgtaatagcgaagaggcccgcaccgatcgcccttcccaacagttgcgcagcctgaatggcgaatggcgcccgggagctgcatgtgtcagaggttttcaccgtcatcaccgaaacgcgcga

hphMX6

nmt41 prom

MCS

HisMyc tag

nmt term

ClonNat

beta-lactamase (AmpR)

**pINTH41EGFPN**

gacgaaagggcctcgtgatacgcctatttttataggttaatgtcatgataataatggtttcttagacgtcaggtggcacttttcggggaaatgtgcgcggaacccctatttgtttatttttctaaatacattcaaatatgtatccgctcatgagacaataaccctgataaatgcttcaataatattgaaaaaggaagagtatgagtattcaacatttccgtgtcgcccttattcccttttttgcggcattttgccttcctgtttttgctcacccagaaacgctggtgaaagtaaaagatgctgaagatcagttgggtgcacgagtgggttacatcgaactggatctcaacagcggtaagatccttgagagttttcgccccgaagaacgttttccaatgatgagcacttttaaagttctgctatgtggcgcggtattatcccgtattgacgccgggcaagagcaactcggtcgccgcatacactattctcagaatgacttggttgagtactcaccagtcacagaaaagcatcttacggatggcatgacagtaagagaattatgcagtgctgccataaccatgagtgataacactgcggccaacttacttctgacaacgatcggaggaccgaaggagctaaccgcttttttgcacaacatgggggatcatgtaactcgccttgatcgttgggaaccggagctgaatgaagccataccaaacgacgagcgtgacaccacgatgcctgtagcaatggcaacaacgttgcgcaaactattaactggcgaactacttactctagcttcccggcaacaattaatagactggatggaggcggataaagttgcaggaccacttctgcgctcggcccttccggctggctggtttattgctgataaatctggagccggtgagcgtgggtctcgcggtatcattgcagcactggggccagatggtaagccctcccgtatcgtagttatctacacgacggggagtcaggcaactatggatgaacgaaatagacagatcgctgagataggtgcctcactgattaagcattggtaactgtcagaccaagtttactcatatatactttagattgatttaaaacttcatttttaatttaaaaggatctaggtgaagatcctttttgataatctcatgaccaaaatcccttaacgtgagttttcgttccactgagcgtcagaccccgtagaaaagatcaaaggatcttcttgagatcctttttttctgcgcgtaatctgctgcttgcaaacaaaaaaaccaccgctaccagcggtggtttgtttgccggatcaagagctaccaactctttttccgaaggtaactggcttcagcagagcgcagataccaaatactgttcttctagtgtagccgtagttaggccaccacttcaagaactctgtagcaccgcctacatacctcgctctgctaatcctgttaccagtggctgctgccagtggcgataagtcgtgtcttaccgggttggactcaagacgatagttaccggataaggcgcagcggtcgggctgaacggggggttcgtgcacacagcccagcttggagcgaacgacctacaccgaactgagatacctacagcgtgagctatgagaaagcgccacgcttcccgaagggagaaaggcggacaggtatccggtaagcggcagggtcggaacaggagagcgcacgagggagcttccagggggaaacgcctggtatctttatagtcctgtcgggtttcgccacctctgacttgagcgtcgatttttgtgatgctcgtcaggggggcggagcctatggaaaaacgccagcaacgcggcctttttacggttcctggccttttgctggccttttgctcacatgttctttcctgcgttatcccctgattctgtggataaccgtattaccgcctttgagtgagctgataccgctcgccgcagccgaacgaccgagcgcagcgagtcagtgagcgaggaagcggaagagcgcccaatacgcaaaccgcctctccccgcgcgttggccgattcattaatgcagctggcacgacaggtttcccgactggaaagcgggcagtgagcgcaacgcaattaatgtgagttagctcactcattaggcaccccaggctttacactttatgcttccggctcgtatgttgtgtggaattgtgagcggataacaatttcacacaggaaacagctatgaccatgattacgccaagcttgcggccgcgttaattaaggcgcgccagatctgtttagcttgcctcgtccccgccgggtcacccggccagcgacatggaggcccagaataccctccttgacagtcttgacgtgcgcagctcaggggcatgatgtgactgtcgcccgtacatttagcccatacatccccatgtataatcatttgcatccatacattttgatggccgcacggcgcgaagcaaaaattacggctcctcgctgcggacctgcgagcagggaaacgctcccctcacagacgcgttgaattgtccccacgccgcgcccctgtagagaaatataaaaggttaggatttgccactgaggttcttctttcatatacttccttttaaaatcttgctaggatacagttctcacatcacatccgaacataaacaaccatgggtaaaaagcctgaactcaccgcgacgtctgtcgagaagtttctgatcgaaaagttcgacagcgtctccgacctgatgcggctctcggagggcgaagaatctcgtgctttcagcttcgatgtaggagggcgtggatatgtcctgcgggtaaatagctgcgccgatggtttctacaaagatcgttatgtttatcggcactttgcatcggccgcgctcccgattccggaagtgcttgacattggggaattcagcgagagcctgacctattgcatctcccgccgtgcacagggtgtcacgttgcaagacctgcctgaaaccgaacctgcccgctgttctgcaaccggtcgcggaggccatggatgcgatcgctgcggccgatcttagccagacgagcgggttcggcccattcggaccgcaaggaatcctgcaggtcgatcgactctagaggatcagaaaattatcgccataaaagacagaataagtcatcagcggttgtttcatttcctatattttttttttatttttttattttttaataagggaaaatttaacgtctaaggatacagaagattgttagcacattaaagtaataaaggcttaagtagtaagtgccttagcatgttattgtatttcaaaggacataatctaaaataataacaatatcatttctcacaagttattcaattttcttttttttttctaataatatcaagaatgtattatttgtttgacataagtcaactaatttatttaatatgctggattaatcttgcagacatgtaaattaacaagttttagtcaaataacgttgaagtttcaatgaactcaaataatttctctttttttttatataaccataatctgatttatattttccgcagggatcaactgaagttatgacatttggattggatcacttataaccttggtcgccaaataatacaaaaatcagcgttataaaacaaagaaggtttttgttaagaaattaatcctctttcttgataagaaagttgaaccgaaattgcagatactgatatatgaaaataatacccacaattttgggaatagcgcaagcctcaatttaaacaataggtgaggacacatgataatgacctcaatgattgttagaagaaaagagcctcattacaaaatcgaaaaatgaatggttgggtacaagtttccaaaacatggtaaagtggactttgcgtatgagacgtaaatagaaaaaaacacttgttatatgttttctagaattattgttgtctctttatggttggatgatgcaaaatagtaatttcggttagttgctgtaaaacaccacgagacaaatagatatggatatttattaaatcaggaaaaacgtaactctcggctactggatggttcagtcacccaacgattactggggagagaaaacagggcaaaagcaaagcttaaaggaatccgattgtcattcggcaatgtgcagcgaaactaaaaaccggataatggacctgttaatcgaaacattgaagataaaggaagaggaatcctggcatatcatcaattgaataagttgaattaattatttcaatctcattctcactttctgacttatagtcgctttgttaaatcatgagtaaaggagaagaacttttcactggagttgtcccaattcttgttgaattagatggtgatgttaatgggcacaaattttctgtcagtggagagggtgaaggtgatgcaacatacggaaaacttacccttaaatttatttgcactactggaaaactacctgttccatggccaacacttgtcactactttgacttatggtgttcaatgcttttcaagatacccagatcacatgaaacagcatgactttttcaagagtgccatgcccgaaggttatgtacaggaaagaactatatttttcaaagatgacgggaactacaagacacgtgctgaagtcaagtttgaaggtgatacccttgttaatagaatcgagttaaaaggtattgattttaaagaagatggaaacattcttggacacaaattggaatacaactataactcacacaatgtatacatcatggcagacaaacaaaagaatggaatcaaagttaacttcaaaattagacacaacattgaagatggaagcgttcaactagcagaccattatcaacaaaatactccaattggcgatggccctgtccttttaccagacaaccattacctgtccacacaatctgccctttcgaaagatcccaacgaaaagagagaccacatggtccttcttgagtttgtaacagctgctgggattacacatggcatggatgaactatacaaacatatgtcgactctagaggatccccgggtaaaaggaatgtctcccttgccagtactgctagggtttttctttcaaactatggaagcccattcaagctgcatattacgattttgtttttcgcttttagaaagtggtttagatgagataatagaaaaattcttgatctccgacaacgagtacttttattttttttgctaatcactttactcaatattagctcgaaatcgtagaaacgtagacgggtgcgggataccgagtggtgtagttaagaatttttataaaccacgtggcccaaaaatatgaacccaaaacgtttatacatgagtatactttaagaaggctataccccttcgtgttagatgtagttttagctacccaacccgagtctatgagcttgacttcagatgtagaaggcattaaatcgttttgaatattaattaaaaaacgatgaaaattaaatatttaaaagcaatcatacgctgaaaatttagtgctgtggctaatccttcaacatggaaatgccataaaagtgactttgacaaaaaaaaaagtatatacaggtagtaaactcatctacttcattgactttgtttacagcatgtggaaggaggaatatttattgctaaatcgtagtttaacattcaataagtaatactattgaaattcgacaagattggccgcatggatgaaaaagaggcattttgctttgggagaattagttcaaattagaactgaaaaaaaaaactttacgaggcaaaaatgtcggattgagatcgtaaaagttcgctcgtcgtcttttgctttgtgattgttttcatggatacatcttgctggatatttaaattttagtactatgtataagatattctataaatgttttatcacccaaacctgttagcgccttcttaattctattcaatctggcttttgctctgagactacttcttggactttcactacttgttagttatacggaatttgtgtaattagaagtgaaataatcctttctattagtaatgcgagctcggcgcgccagatctgtttagcttgcctcgtccccgccgggtcacccggccagcgacatggaggcccagaataccctccttgacagtcttgacgtgcgcagctcaggggcatgatgtgactgtcgcccgtacatttagcccatacatccccatgtataatcatttgcatccatacattttgatggccgcacggcgcgaagcaaaaattacggctcctcgctgccgacctgcgagcagggaaacgctcccctcacagacgcgttgaattgtccccacgccgcgcccctgtagagaaatataaaaggttaggatttgccactgaggttcttctttcatatacttcctttttaaatcttgctaggatacagttctcacatcacatccgaacataaacaaccatgggtaccactcttgacgacacggcttaccggtaccgcaccagtgtcccaggggacgccgaggccatcgaggcactggatgggtccttcaccaccgacaccgtcttccgcgtcaccgccaccggggacggcttcaccctgcgggaggtgccggtggacccgcccctgaccaaggtgttccccgacgacgaatcggacgacgaatcggacgacggggaggacggcgacccggactctcggacgttcgtcgcgtacggggacgacggcgacctggcgggcttcgtggtcgtctcgtactccggctggaaccgccggctgaccgtcgaggacatcgaggtcgccccggagcaccgggggcacggggtcgggcgcgcgttgatggggctcgtgacggagttcgcccgcgagcggggtgccgggcacctctggctggaggtcaccaacgtcaacgcaccggcgatccacgcgtaccggcggatggggttcaccctctgcggcctggacaccgccctgtacgacggcaccgcctcggacggcgagcaggcgctctacatgagcatgccctgcccctaatcagtactgacaataaaaagattcttgttttcaagaacttgtcatttgtatagtttttttatattgtagttgttctattttaatcaaatgttagcgtgatttatatttttttcgcctcgacatcatctgcccagatgcgaagttaagtgcgcagaaagtaatatcatgcgtcaatcgtatgtgaatgctggtcgctatactgctgtcgattcgatactaacgccgccatccagaattcggtcaatacactacatggcgtgatttcatttgcgcgattgctgatccccatgtgtatcactggcaaactgtgatggacgacaccgtcggtgcgtccgtcgcgcaggctctcgatgagctgatgctttgggccgaggactgccccgaagtccggcacctcgtgcacgcggatttcggctccaacaatgtcctgacggacaatggccgcataacagcggtcattgactggagcgaggcgatgttcggggattcccaatacgaggtcgccaacatcttcttctggaggccgtggttggcttgtatggagcagcagacgcgctacttcgagcggaggcatccggagcttgcaggatcgccgcggctccgggcgtatatgctccgcattggtcttgaccaactctatcagagcttggttgacggcaatttcgatgatgcagcttgggcgcagggtcgatgcgacgcaatcgtccgatccggagccgggactgtcgggcgtacacaaatcgcccgcagaagcgcggccgtctggaccgatggctgtgtagaagtactcgccgatagtggaaaccgacgccccagcactcgtccgagggcaaaggaataatcagtactgacaataaaaagattcttgttttcaagaacttgtcatttgtatagtttttttatattgtagttgttctattttaatcaaatgttagcgtgatttatattttttttcgcctcgacatcatctgcccagatgcgaagttaagtgcgcagaaagtaatatcatgcgtcaatcgtatgtgaatgctggtcgctatactgctgtcgattcgatactaacgccgccatccagtttaaagcggccgcgaattcactggccgtcgttttacaacgtcgtgactgggaaaaccctggcgttacccaacttaatcgccttgcagcacatccccctttcgccagctggcgtaatagcgaagaggcccgcaccgatcgcccttcccaacagttgcgcagcctgaatggcgaatggcgcccgggagctgcatgtgtcagaggttttcaccgtcatcaccgaaacgcgcga

hphMX6

nmt41 prom

MCS

eGFP tag

nmt term

ClonNat

beta-lactamase (AmpR)

**pINTH41EGFPC**

gacgaaagggcctcgtgatacgcctatttttataggttaatgtcatgataataatggtttcttagacgtcaggtggcacttttcggggaaatgtgcgcggaacccctatttgtttatttttctaaatacattcaaatatgtatccgctcatgagacaataaccctgataaatgcttcaataatattgaaaaaggaagagtatgagtattcaacatttccgtgtcgcccttattcccttttttgcggcattttgccttcctgtttttgctcacccagaaacgctggtgaaagtaaaagatgctgaagatcagttgggtgcacgagtgggttacatcgaactggatctcaacagcggtaagatccttgagagttttcgccccgaagaacgttttccaatgatgagcacttttaaagttctgctatgtggcgcggtattatcccgtattgacgccgggcaagagcaactcggtcgccgcatacactattctcagaatgacttggttgagtactcaccagtcacagaaaagcatcttacggatggcatgacagtaagagaattatgcagtgctgccataaccatgagtgataacactgcggccaacttacttctgacaacgatcggaggaccgaaggagctaaccgcttttttgcacaacatgggggatcatgtaactcgccttgatcgttgggaaccggagctgaatgaagccataccaaacgacgagcgtgacaccacgatgcctgtagcaatggcaacaacgttgcgcaaactattaactggcgaactacttactctagcttcccggcaacaattaatagactggatggaggcggataaagttgcaggaccacttctgcgctcggcccttccggctggctggtttattgctgataaatctggagccggtgagcgtgggtctcgcggtatcattgcagcactggggccagatggtaagccctcccgtatcgtagttatctacacgacggggagtcaggcaactatggatgaacgaaatagacagatcgctgagataggtgcctcactgattaagcattggtaactgtcagaccaagtttactcatatatactttagattgatttaaaacttcatttttaatttaaaaggatctaggtgaagatcctttttgataatctcatgaccaaaatcccttaacgtgagttttcgttccactgagcgtcagaccccgtagaaaagatcaaaggatcttcttgagatcctttttttctgcgcgtaatctgctgcttgcaaacaaaaaaaccaccgctaccagcggtggtttgtttgccggatcaagagctaccaactctttttccgaaggtaactggcttcagcagagcgcagataccaaatactgttcttctagtgtagccgtagttaggccaccacttcaagaactctgtagcaccgcctacatacctcgctctgctaatcctgttaccagtggctgctgccagtggcgataagtcgtgtcttaccgggttggactcaagacgatagttaccggataaggcgcagcggtcgggctgaacggggggttcgtgcacacagcccagcttggagcgaacgacctacaccgaactgagatacctacagcgtgagctatgagaaagcgccacgcttcccgaagggagaaaggcggacaggtatccggtaagcggcagggtcggaacaggagagcgcacgagggagcttccagggggaaacgcctggtatctttatagtcctgtcgggtttcgccacctctgacttgagcgtcgatttttgtgatgctcgtcaggggggcggagcctatggaaaaacgccagcaacgcggcctttttacggttcctggccttttgctggccttttgctcacatgttctttcctgcgttatcccctgattctgtggataaccgtattaccgcctttgagtgagctgataccgctcgccgcagccgaacgaccgagcgcagcgagtcagtgagcgaggaagcggaagagcgcccaatacgcaaaccgcctctccccgcgcgttggccgattcattaatgcagctggcacgacaggtttcccgactggaaagcgggcagtgagcgcaacgcaattaatgtgagttagctcactcattaggcaccccaggctttacactttatgcttccggctcgtatgttgtgtggaattgtgagcggataacaatttcacacaggaaacagctatgaccatgattacgccaagcttgcggccgcgttaattaaggcgcgccagatctgtttagcttgcctcgtccccgccgggtcacccggccagcgacatggaggcccagaataccctccttgacagtcttgacgtgcgcagctcaggggcatgatgtgactgtcgcccgtacatttagcccatacatccccatgtataatcatttgcatccatacattttgatggccgcacggcgcgaagcaaaaattacggctcctcgctgcggacctgcgagcagggaaacgctcccctcacagacgcgttgaattgtccccacgccgcgcccctgtagagaaatataaaaggttaggatttgccactgaggttcttctttcatatacttccttttaaaatcttgctaggatacagttctcacatcacatccgaacataaacaaccatgggtaaaaagcctgaactcaccgcgacgtctgtcgagaagtttctgatcgaaaagttcgacagcgtctccgacctgatgcggctctcggagggcgaagaatctcgtgctttcagcttcgatgtaggagggcgtggatatgtcctgcgggtaaatagctgcgccgatggtttctacaaagatcgttatgtttatcggcactttgcatcggccgcgctcccgattccggaagtgcttgacattggggaattcagcgagagcctgacctattgcatctcccgccgtgcacagggtgtcacgttgcaagacctgcctgaaaccgaacctgcccgctgttctgcaaccggtcgcggaggccatggatgcgatcgctgcggccgatcttagccagacgagcgggttcggcccattcggaccgcaaggaatcctgcaggtcgatcgactctagaggatcagaaaattatcgccataaaagacagaataagtcatcagcggttgtttcatttcctatattttttttttatttttttattttttaataagggaaaatttaacgtctaaggatacagaagattgttagcacattaaagtaataaaggcttaagtagtaagtgccttagcatgttattgtatttcaaaggacataatctaaaataataacaatatcatttctcacaagttattcaattttcttttttttttctaataatatcaagaatgtattatttgtttgacataagtcaactaatttatttaatatgctggattaatcttgcagacatgtaaattaacaagttttagtcaaataacgttgaagtttcaatgaactcaaataatttctctttttttttatataaccataatctgatttatattttccgcagggatcaactgaagttatgacatttggattggatcacttataaccttggtcgccaaataatacaaaaatcagcgttataaaacaaagaaggtttttgttaagaaattaatcctctttcttgataagaaagttgaaccgaaattgcagatactgatatatgaaaataatacccacaattttgggaatagcgcaagcctcaatttaaacaataggtgaggacacatgataatgacctcaatgattgttagaagaaaagagcctcattacaaaatcgaaaaatgaatggttgggtacaagtttccaaaacatggtaaagtggactttgcgtatgagacgtaaatagaaaaaaacacttgttatatgttttctagaattattgttgtctctttatggttggatgatgcaaaatagtaatttcggttagttgctgtaaaacaccacgagacaaatagatatggatatttattaaatcaggaaaaacgtaactctcggctactggatggttcagtcacccaacgattactggggagagaaaacagggcaaaagcaaagcttaaaggaatccgattgtcattcggcaatgtgcagcgaaactaaaaaccggataatggacctgttaatcgaaacattgaagatggaagaggaatcctggcatatcatcaattgaataagttgaattaattatttcaatctcattctcactttctgacttatagtcgctttgttaaatcatatgtcgacatggtaccagatctctcgaggatccccgggtatgagtaaaggagaagaacttttcactggagttgtcccaattcttgttgaattagatggtgatgttaatgggcacaaattttctgtcagtggagagggtgaaggtgatgcaacatacggaaaacttacccttaaatttatttgcactactggaaaactacctgttccatggccaacacttgtcactactttgacttatggtgttcaatgcttttcaagatacccagatcacatgaaacagcatgactttttcaagagtgccatgcccgaaggttatgtacaggaaagaactatatttttcaaagatgacgggaactacaagacacgtgctgaagtcaagtttgaaggtgatacccttgttaatagaatcgagttaaaaggtattgattttaaagaagatggaaacattcttggacacaaattggaatacaactataactcacacaatgtatacatcatggcagacaaacaaaagaatggaatcaaagttaacttcaaaattagacacaacattgaagatggaagcgttcaactagcagaccattatcaacaaaatactccaattggcgatggccctgtccttttaccagacaaccattacctgtccacacaatctgccctttcgaaagatcccaacgaaaagagagaccacatggtccttcttgagtttgtaacagctgctgggattacacatggcatggatgaactatacaaacatacgtaaccatggggtaaaaggaatgtctcccttgccagtactgctagggtttttctttcaaactatggaagcccattcaagctgcatattacgattttgtttttcgcttttagaaagtggtttagatgagataatagaaaaattcttgatctccgacaacgagtacttttattttttttgctaatcactttactcaatattagctcgaaatcgtagaaacgtagacgggtgcgggataccgagtggtgtagttaagaatttttataaaccacgtggcccaaaaatatgaacccaaaacgtttatacatgagtatactttaagaaggctataccccttcgtgttagatgtagttttagctacccaacccgagtctatgagcttgacttcagatgtagaaggcattaaatcgttttgaatattaattaaaaaacgatgaaaattaaatatttaaaagcaatcatacgctgaaaatttagtgctgtggctaatccttcaacatggaaatgccataaaagtgactttgacaaaaaaaaaagtatatacaggtagtaaactcatctacttcattgactttgtttacagcatgtggaaggaggaatatttattgctaaatcgtagtttaacattcaataagtaatactattgaaattcgacaagattggccgcatggatgaaaaagaggcattttgctttgggagaattagttcaaattagaactgaaaaaaaaaactttacgaggcaaaaatgtcggattgagatcgtaaaagttcgctcgtcgtcttttgctttgtgattgttttcatggatacatcttgctggatatttaaattttagtactatgtataagatattctataaatgttttatcacccaaacctgttagcgccttcttaattctattcaatctggcttttgctctgagactacttcttggactttcactacttgttagttatacggaatttgtgtaattagaagtgaaataatcctttctattagtaatgcgagctcggcgcgccagatctgtttagcttgcctcgtccccgccgggtcacccggccagcgacatggaggcccagaataccctccttgacagtcttgacgtgcgcagctcaggggcatgatgtgactgtcgcccgtacatttagcccatacatccccatgtataatcatttgcatccatacattttgatggccgcacggcgcgaagcaaaaattacggctcctcgctgccgacctgcgagcagggaaacgctcccctcacagacgcgttgaattgtccccacgccgcgcccctgtagagaaatataaaaggttaggatttgccactgaggttcttctttcatatacttcctttttaaatcttgctaggatacagttctcacatcacatccgaacataaacaaccatgggtaccactcttgacgacacggcttaccggtaccgcaccagtgtcccaggggacgccgaggccatcgaggcactggatgggtccttcaccaccgacaccgtcttccgcgtcaccgccaccggggacggcttcaccctgcgggaggtgccggtggacccgcccctgaccaaggtgttccccgacgacgaatcggacgacgaatcggacgacggggaggacggcgacccggactctcggacgttcgtcgcgtacggggacgacggcgacctggcgggcttcgtggtcgtctcgtactccggctggaaccgccggctgaccgtcgaggacatcgaggtcgccccggagcaccgggggcacggggtcgggcgcgcgttgatggggctcgtgacggagttcgcccgcgagcggggtgccgggcacctctggctggaggtcaccaacgtcaacgcaccggcgatccacgcgtaccggcggatggggttcaccctctgcggcctggacaccgccctgtacgacggcaccgcctcggacggcgagcaggcgctctacatgagcatgccctgcccctaatcagtactgacaataaaaagattcttgttttcaagaacttgtcatttgtatagtttttttatattgtagttgttctattttaatcaaatgttagcgtgatttatatttttttcgcctcgacatcatctgcccagatgcgaagttaagtgcgcagaaagtaatatcatgcgtcaatcgtatgtgaatgctggtcgctatactgctgtcgattcgatactaacgccgccatccagaattcggtcaatacactacatggcgtgatttcatttgcgcgattgctgatccccatgtgtatcactggcaaactgtgatggacgacaccgtcggtgcgtccgtcgcgcaggctctcgatgagctgatgctttgggccgaggactgccccgaagtccggcacctcgtgcacgcggatttcggctccaacaatgtcctgacggacaatggccgcataacagcggtcattgactggagcgaggcgatgttcggggattcccaatacgaggtcgccaacatcttcttctggaggccgtggttggcttgtatggagcagcagacgcgctacttcgagcggaggcatccggagcttgcaggatcgccgcggctccgggcgtatatgctccgcattggtcttgaccaactctatcagagcttggttgacggcaatttcgatgatgcagcttgggcgcagggtcgatgcgacgcaatcgtccgatccggagccgggactgtcgggcgtacacaaatcgcccgcagaagcgcggccgtctggaccgatggctgtgtagaagtactcgccgatagtggaaaccgacgccccagcactcgtccgagggcaaaggaataatcagtactgacaataaaaagattcttgttttcaagaacttgtcatttgtatagtttttttatattgtagttgttctattttaatcaaatgttagcgtgatttatattttttttcgcctcgacatcatctgcccagatgcgaagttaagtgcgcagaaagtaatatcatgcgtcaatcgtatgtgaatgctggtcgctatactgctgtcgattcgatactaacgccgccatccagtttaaagcggccgcgaattcactggccgtcgttttacaacgtcgtgactgggaaaaccctggcgttacccaacttaatcgccttgcagcacatccccctttcgccagctggcgtaatagcgaagaggcccgcaccgatcgcccttcccaacagttgcgcagcctgaatggcgaatggcgcctgatgcggtattttctccttacgcatctgtgcggtatttcacaccgcatatatggtgcactctcagtacaatctgctctgatgccgcatagttaagccagccccgacacccgccaacacccgctgacgcgccctgacgggcttgtctgctcccggcatccgcttacagacaagctgtgaccgtctccgggagctgcatgtgtcagaggttttcaccgtcatcaccgaaacgcgcga

hphMX6

nmt41 prom

MCS

eGFP tag

nmt term

ClonNat

beta-lactamase (AmpR)

**pINTH1**

gacgaaagggcctcgtgatacgcctatttttataggttaatgtcatgataataatggtttcttagacgtcaggtggcacttttcggggaaatgtgcgcggaacccctatttgtttatttttctaaatacattcaaatatgtatccgctcatgagacaataaccctgataaatgcttcaataatattgaaaaaggaagagtatgagtattcaacatttccgtgtcgcccttattcccttttttgcggcattttgccttcctgtttttgctcacccagaaacgctggtgaaagtaaaagatgctgaagatcagttgggtgcacgagtgggttacatcgaactggatctcaacagcggtaagatccttgagagttttcgccccgaagaacgttttccaatgatgagcacttttaaagttctgctatgtggcgcggtattatcccgtattgacgccgggcaagagcaactcggtcgccgcatacactattctcagaatgacttggttgagtactcaccagtcacagaaaagcatcttacggatggcatgacagtaagagaattatgcagtgctgccataaccatgagtgataacactgcggccaacttacttctgacaacgatcggaggaccgaaggagctaaccgcttttttgcacaacatgggggatcatgtaactcgccttgatcgttgggaaccggagctgaatgaagccataccaaacgacgagcgtgacaccacgatgcctgtagcaatggcaacaacgttgcgcaaactattaactggcgaactacttactctagcttcccggcaacaattaatagactggatggaggcggataaagttgcaggaccacttctgcgctcggcccttccggctggctggtttattgctgataaatctggagccggtgagcgtgggtctcgcggtatcattgcagcactggggccagatggtaagccctcccgtatcgtagttatctacacgacggggagtcaggcaactatggatgaacgaaatagacagatcgctgagataggtgcctcactgattaagcattggtaactgtcagaccaagtttactcatatatactttagattgatttaaaacttcatttttaatttaaaaggatctaggtgaagatcctttttgataatctcatgaccaaaatcccttaacgtgagttttcgttccactgagcgtcagaccccgtagaaaagatcaaaggatcttcttgagatcctttttttctgcgcgtaatctgctgcttgcaaacaaaaaaaccaccgctaccagcggtggtttgtttgccggatcaagagctaccaactctttttccgaaggtaactggcttcagcagagcgcagataccaaatactgttcttctagtgtagccgtagttaggccaccacttcaagaactctgtagcaccgcctacatacctcgctctgctaatcctgttaccagtggctgctgccagtggcgataagtcgtgtcttaccgggttggactcaagacgatagttaccggataaggcgcagcggtcgggctgaacggggggttcgtgcacacagcccagcttggagcgaacgacctacaccgaactgagatacctacagcgtgagctatgagaaagcgccacgcttcccgaagggagaaaggcggacaggtatccggtaagcggcagggtcggaacaggagagcgcacgagggagcttccagggggaaacgcctggtatctttatagtcctgtcgggtttcgccacctctgacttgagcgtcgatttttgtgatgctcgtcaggggggcggagcctatggaaaaacgccagcaacgcggcctttttacggttcctggccttttgctggccttttgctcacatgttctttcctgcgttatcccctgattctgtggataaccgtattaccgcctttgagtgagctgataccgctcgccgcagccgaacgaccgagcgcagcgagtcagtgagcgaggaagcggaagagcgcccaatacgcaaaccgcctctccccgcgcgttggccgattcattaatgcagctggcacgacaggtttcccgactggaaagcgggcagtgagcgcaacgcaattaatgtgagttagctcactcattaggcaccccaggctttacactttatgcttccggctcgtatgttgtgtggaattgtgagcggataacaatttcacacaggaaacagctatgaccatgattacgccaagcttgcggccgcgttaattaaggcgcgccagatctgtttagcttgcctcgtccccgccgggtcacccggccagcgacatggaggcccagaataccctccttgacagtcttgacgtgcgcagctcaggggcatgatgtgactgtcgcccgtacatttagcccatacatccccatgtataatcatttgcatccatacattttgatggccgcacggcgcgaagcaaaaattacggctcctcgctgcggacctgcgagcagggaaacgctcccctcacagacgcgttgaattgtccccacgccgcgcccctgtagagaaatataaaaggttaggatttgccactgaggttcttctttcatatacttccttttaaaatcttgctaggatacagttctcacatcacatccgaacataaacaaccatgggtaaaaagcctgaactcaccgcgacgtctgtcgagaagtttctgatcgaaaagttcgacagcgtctccgacctgatgcggctctcggagggcgaagaatctcgtgctttcagcttcgatgtaggagggcgtggatatgtcctgcgggtaaatagctgcgccgatggtttctacaaagatcgttatgtttatcggcactttgcatcggccgcgctcccgattccggaagtgcttgacattggggaattcagcgagagcctgacctattgcatctcccgccgtgcacagggtgtcacgttgcaagacctgcctgaaaccgaacctgcccgctgttctgcaaccggtcgcggaggccatggatgcgatcgctgcggccgatcttagccagacgagcgggttcggcccattcggaccgcaaggaatcctgcaggtcgatcgactctagaggatcagaaaattatcgccataaaagacagaataagtcatcagcggttgtttcatttcctatattttttttttatttttttattttttaataagggaaaatttaacgtctaaggatacagaagattgttagcacattaaagtaataaaggcttaagtagtaagtgccttagcatgttattgtatttcaaaggacataatctaaaataataacaatatcatttctcacaagttattcaattttcttttttttttctaataatatcaagaatgtattatttgtttgacataagtcaactaatttatttaatatgctggattaatcttgcagacatgtaaattaacaagttttagtcaaataacgttgaagtttcaatgaactcaaataatttctctttttttttatataaccataatctgatttatattttccgcagggatcaactgaagttatgacatttggattggatcacttataaccttggtcgccaaataatacaaaaatcagcgttataaaacaaagaaggtttttgttaagaaattaatcctctttcttgataagaaagttgaaccgaaattgcagatactgatatatgaaaataatacccacaattttgggaatagcgcaagcctcaatttaaacaataggtgaggacacatgataatgacctcaatgattgttagaagaaaagagcctcattacaaaatcgaaaaatgaatggttgggtacaagtttccaaaacatggtaaagtggactttgcgtatgagacgtaaatagaaaaaaacacttgttatatgttttctagaattattgttgtctctttatggttggatgatgcaaaatagtaatttcggttagttgctgtaaaacaccacgagacaaatagatatggatatttattaaatcaggaaaaacgtaactctcggctactggatggttcagtcacccaacgattactggggagagaaaacagggcaaaagcaaagcttaaaggaatccgattgtcattcggcaatgtgcagcgaaactaaaaaccggataatggacctgttaatcgaaacattgaagatatataaaggaagaggaatcctggcatatcatcaattgaataagttgaattaattatttcaatctcattctcactttctgacttatagtcgctttgttaaatcatatgtcgactctagaggatccccgggtaaaaggaatgtctcccttgccagtactgctagggtttttctttcaaactatggaagcccattcaagctgcatattacgattttgtttttcgcttttagaaagtggtttagatgagataatagaaaaattcttgatctccgacaacgagtacttttattttttttgctaatcactttactcaatattagctcgaaatcgtagaaacgtagacgggtgcgggataccgagtggtgtagttaagaatttttataaaccacgtggcccaaaaatatgaacccaaaacgtttatacatgagtatactttaagaaggctataccccttcgtgttagatgtagtgttccccgacgacgaatcggacgacgaatcggacgacggggaggacggcgacccggactctcggacgttcgtcgcgtacggggacgacggcgacctggcgggcttcgtggtcgtctcgtactccggctggaaccgccggctgaccgtcgaggacatcgaggtcgccccggagcaccgggggcacggggtcgggcgcgcgttgatggggctcgtgacggagttcgcccgcgagcggggtgccgggcacctctggctggaggtcaccaacgtcaacgcaccggcgatccacgcgtaccggcggatggggttcaccctctgcggcctggacaccgccctgtacgacggcaccgcctcggacggcgagcaggcgctctacatgagcatgccctgcccctaatcagtactgacaataaaaagattcttgttttcaagaacttgtcatttgtatagtttttttatattgtagttgttctattttaatcaaatgttagcgtgatttatatttttttcgcctcgacatcatctgcccagatgcgaagttaagtgcgcagaaagtaatatcatgcgtcaatcgtatgtgaatgctggtcgctatactgctgtcgattcgatactaacgccgccatccagaattcggtcaatacactacatggcgtgatttcatttgcgcgattgctgatccccatgtgtatcactggcaaactgtgatggacgacaccgtcggtgcgtccgtcgcgcaggctctcgatgagctgatgctttgggccgaggactgccccgaagtccggcacctcgtgcacgcggatttcggctccaacaatgtcctgacggacaatggccgcataacagcggtcattgactggagcgaggcgatgttcggggattcccaatacgaggtcgccaacatcttcttctggaggccgtggttggcttgtatggagcagcagacgcgctacttcgagcggaggcatccggagcttgcaggatcgccgcggctccgggcgtatatgctccgcattggtcttgaccaactctatcagagcttggttgacggcaatttcgatgatgcagcttgggcgcagggtcgatgcgacgcaatcgtccgatccggagccgggactgtcgggcgtacacaaatcgcccgcagaagcgcggccgtctggaccgatggctgtgtagaagtactcgccgatagtggaaaccgacgccccagcactcgtccgagggcaaaggaataatcagtactgacaataaaaagattcttgttttcaagaacttgtcatttgtatagtttttttatattgtagttgttctattttaatcaaatgttagcgtgatttatattttttttcgcctcgacatcatctgcccagatgcgaagttaagtgcgcagaaagtaatatcatgcgtcaatcgtatgtgaatgctggtcgctatactgctgtcgattcgatactaacgccgccatccagtttaaagcggccgcgaattcactggccgtcgttttacaacgtcgtgactgggaaaaccctggcgttacccaacttaatcgccttgcagcacatccccctttcgccagctggcgtaatagcgaagaggcccgcaccgatcgcccttcccaacagttgcgcagcctgaatggcgaatggcgccgggagctgcatgtgtcagaggttttcaccgtcatcaccgaaacgcgcga

hphMX6

nmt1 prom

MCS

nmt term

ClonNat

beta-lactamase (AmpR)
