# Supplementary material for: Extending the Schizosaccharomyces pombe Molecular Genetic Toolbox
Source: PLoS One. 2014 May 21;9(5):e97683. doi: 10.1371/journal.pone.0097683 (PMC4029729; doi:10.1371/journal.pone.0097683)
Supplement: Figure S3 — DNA sequences of the pINTH series. (DOCX) [file pone.0097683.s003.docx]

**pINTK**

GGGCGAATTCGCGGCCGCGCAGGAAGATTATCTAACACAAAGTGAAGTAAGGGATACAAAGCACTATCATGAGACTCATTAATGACAAAGCGCTCCAAAGCAATACGCCAATTGACGTATTCGGAAAGCTTGGTATTGAAGCCAAAAGTGGAAAGAGTTCCTAGGAATTGGTTGAAACGAAGTCGGGGATGACTAGTTACGTGAGCAACAATTACTCCTTGTTCGGGATGGAAAGCACTGGCAGTTACCACGCGAGCAACGTGATCGGCAGGAACCATATTTACAGTATTATTGATGTTCGCGTACAGACCCAATTCAATGCACCCTTTGACCTTCGACCAAAAGTCGTCGGTATTAATAGCACCAGACTTGGAGTCGCCTAAAATGTATCCAGGACGCGCAGCAGCGCCACGAAGGCCACGTAATCCCGCTTGCCTTACTAAATATTCAGATACCCATTTGCTTTGTCCGTAACCGGTATGCAAATCTTTCGAAGAACCTTGCAAAGGATCGGATTCTGGGATTCCATTTCCTCCCTTTGAAGTAATTTCGTTCGAAAGGTTAACGTAATACTCAGTATCAACAGTTGATGTGGATGAAACAAAACTCAAGGATTTACCTTTTCCTAAAGAGCAAAGTTTTAATGCAGTAATTGTACCCAGGTACCATAACTTCGTATAGCATACATTATACGAAGTTATTTGACATCTAATTTATTCTGTTCCAACACCAATGTTTATAACCAAGTTTTATCTTGTTTGTCTACATGGTATTTTACATTCATCTACATACATCTTTCATTGGCTTTGTACATAGTTATCATTACAAGTCTAAAAAAATTCACTCTTTTCTTATTCAATGTCAATCCAAGAGAAAAGATTGTGGTAATGTTGTAGGAGCATGTTTAATAAATTACTATAGCAAATTACTTTTTATTCCCAAGGTGTTTATCTATAATAGTTAATATTTTAGTCGCTACATAAAATTTTACCAAAGAGTACTTGTATACTAATTCTAAATGCCTTCTGACATAAAACGCCTAGGAAAACAAACGCAAACAAGGCATCGACTTTTTCAATAACCAACCAAAAAAATTTTACATTAGTCTTTTTTTAATGCTGAGAAAGTCTTTGCTGATATGCCTTCCAACCAGCTTCTCTATATCTCTTGGCTTCGACAACAGGATTACGACCAGCTCCATAGACTCCACGACCAACAATGATGATATCGCTACCGCAGTTTACAATCACTTCTTCAGGAGTACGATATTGCTGTCCCAGCCCGTCTCCTTTAACATCCAAGCCGATACCAGGGGACATAGTTATGTAGTCGCTTTGAAGGTTAGGAAATCGACGACCAGCTATAAAGCCAAAGCAAAAATCGGTATGCTTCTCAAACCATTCTAAGGTTTTCTCTGTGTAGGAACCAGTAGCCAAAGAGCCTTTGGAAGACATTTCAGCCAAAAGCAAGAGACCACGTCCCAAAGGTAAACCAACTTCTTTGAGGCCTTGTATAATACCCTCGCCTGGCACTGTATGGCAATTTGTGATATGAGCCCAAGAAGCAATTTTGTACACACCAGATGCATATTGTAGCTTGACGGTATTTCCAATGTCTGCGAATTTGCGATCCTCAAAGATAAGAAAACGATGCTTTTTACCTAAGGCCACCAGTTTTTCTACCATATCCTGGTCGAAATCCTCGACAACGTCAATATGTGTCTTGATAACACAGACATAGGGTCCAATTTTATCTACCAATTCTAAGATTTCGGATTTCTTCGTCAAATCGACCGCGACTGACAAGTTGCTTTGCTTTTCTTCCATCAAAGCCAACAATTCCTTGGCAATGGGATTTTTCATCCCCTCAGCTCTAGCTGAATAGCTTTGAAATACTCTAGCATCCATAACTTTGCTTTTAAACCTTTAATTTCGATCCAAGCAAAAAAGAGGTTCTTGGTAGGACAATACGGTAAGAAAACACGACATGTGCAGAGATGCCGACGAAGCATAGTTAAACTGGGATGGTAAAATCAATTAAGAATTTATAAAGACAAAATTGTATAAGTCTCTAAAACATCTTAATTATACCTCACAGAACTATCTAAAATATATTCACAAAGTGCAAACATTATCATGAAAAAGAACCATTTTAATTTAAAGCAAGGGCATTAAGGCTTATTTACAGAATTTCTTACTTTTGTAAAGATTATAAGGCTGATTATCTTTTTCACCATGCCAAAAATTACACAAGATAGAATGGATGTTTGAAATTAAACGTGAGTATACAAACAAATACACTAGGTAAATCGAAACATTTTTTTCTCCATTAAGTAACAAATTCCTATTTAGAGAAAGAATGCTGAGTAGATTAAATAATCTATACAAACTTTTTTAACACAAATGCATACATATAGCCAGTGGGATTTGTAGCATAACTTCGTATAGCATACATTATACGAAGTTATGAGCTCCCGGGGATCCTCTAGAGTCGACCTGCAGGAAAGTATGATATGTTGTTGGTAACGGAGAGTGTCAAATGTTCGAACTGCCTAAAACCTTCGTTGTAGATGATAAATTGATAATCTTCATTCACAAACAAAATCCAAAACATATGTTTAATATACTCCAATAATGAAATTGTTTTGTTATATCATTTCAAATTAGTGTTTAAGTTATTTATCGTACATATTTTGTAAATTCAAATGTACTGCTTATTATTGCAATTATTAAACTATTGTGCAAAATAATAATAATAATTTAATTTTATAATAAACATTTGTACAAAGTGTCATATCACAAAGTTAGTCTTCAATTTTATTTCTTCGATATTTGTATTTATCAACAACCCTACGTGTCAGTGCGGCCGCAAGCTTGAGTATTCTATAGTGTCACCTAAATAGCTTGGCGTAATCATGGTCATAGCTGTTTCCTGTGTGAAATTGTTATCCGCTCACAATTCCACACAACATACGAGCCGGAAGCATAAAGTGTAAAGCCTGGGGTGCCTAATGAGTGAGCTAACTCACATTAATTGCGTTGCGCTCACTGCCCGCTTTCCAGTCGGGAAACCTGTCGTGCCAGCTGCATTAATGAATCGGCCAACGCGCGGGGAGAGGCGGTTTGCGTATTGGGCGCTCTTCCGCTTCCTCGCTCACTGACTCGCTGCGCTCGGTCGTTCGGCTGCGGCGAGCGGTATCAGCTCACTCAAAGGCGGTAATACGGTTATCCACAGAATCAGGGGATAACGCAGGAAAGAACATGTGAGCAAAAGGCCAGCAAAAGGCCAGGAACCGTAAAAAGGCCGCGTTGCTGGCGTTTTTCCATAGGCTCCGCCCCCCTGACGAGCATCACAAAAATCGACGCTCAAGTCAGAGGTGGCGAAACCCGACAGGACTATAAAGATACCAGGCGTTTCCCCCTGGAAGCTCCCTCGTGCGCTCTCCTGTTCCGACCCTGCCGCTTACCGGATACCTGTCCGCCTTTCTCCCTTCGGGAAGCGTGGCGCTTTCTCATAGCTCACGCTGTAGGTATCTCAGTTCGGTGTAGGTCGTTCGCTCCAAGCTGGGCTGTGTGCACGAACCCCCCGTTCAGCCCGACCGCTGCGCCTTATCCGGTAACTATCGTCTTGAGTCCAACCCGGTAAGACACGACTTATCGCCACTGGCAGCAGCCACTGGTAACAGGATTAGCAGAGCGAGGTATGTAGGCGGTGCTACAGAGTTCTTGAAGTGGTGGCCTAACTACGGCTACACTAGAAGAACAGTATTTGGTATCTGCGCTCTGCTGAAGCCAGTTACCTTCGGAAAAAGAGTTGGTAGCTCTTGATCCGGCAAACAAACCACCGCTGGTAGCGGTGGTTTTTTTGTTTGCAAGCAGCAGATTACGCGCAGAAAAAAAGGATCTCAAGAAGATCCTTTGATCTTTTCTACGGGGTCTGACGCTCAGTGGAACGAAAACTCACGTTAAGGGATTTTGGTCATGAGATTATCAAAAAGGATCTTCACCTAGATCCTTTTAAATTAAAAATGAAGTTTTAAATCAATCTAAAGTATATATGAGTAAACTTGGTCTGACAGTTACCAATGCTTAATCAGTGAGGCACCTATCTCAGCGATCTGTCTATTTCGTTCATCCATAGTTGCCTGACTCCCCGTCGTGTAGATAACTACGATACGGGAGGGCTTACCATCTGGCCCCAGTGCTGCAATGATACCGCGAGACCCACGCTCACCGGCTCCAGATTTATCAGCAATAAACCAGCCAGCCGGAAGGGCCGAGCGCAGAAGTGGTCCTGCAACTTTATCCGCCTCCATCCAGTCTATTAATTGTTGCCGGGAAGCTAGAGTAAGTAGTTCGCCAGTTAATAGTTTGCGCAACGTTGTTGCCATTGCTACAGGCATCGTGGTGTCACGCTCGTCGTTTGGTATGGCTTCATTCAGCTCCGGTTCCCAACGATCAAGGCGAGTTACATGATCCCCCATGTTGTGCAAAAAAGCGcTTAGCTCCTTCGGTCCTCCGATCGTTGTCAGAAGTAAGTTGGCCGCAGTGTTATCACTCATGGTTATGGCAGCACTGCATAATTCTCTTACTGTCATGCCATCCGTAAGATGCTTTTCTGTGACTGGTGAGTACTCAACCAAGTCATTCTGAGAATAGTGTATGCGGCGACCGAGTTGCTCTTGCCCGGCGTCAATACGGGATAATACCGCGCCACATAGCAGAACTTTAAAAGTGCTCATCATTGGAAAACGTTCTTCGGGGCGAAAACTCTCAAGGATCTTACCGCTGTTGAGATCCAGTTCGATGTAACCCACTCGTGCACCCAACTGATCTTCAGCATCTTTTACTTTCACCAGCGTTTCTGGGTGAGCAAAAACAGGAAGGCAAAATGCCGCAAAAAAGGGAATAAGGGCGACACGGAAATGTTGAATACTCATACTCTTCCTTTTTCAATATTATTGAAGCATTTATCAGGGTTATTGTCTCATGAGCGGATACATATTTGAATGTATTTAGAAAAATAAACAAATAGGGGTTCCGCGCACATTTCCCCGAAAAGTGCCACCTGACGTCTAAGAAACCATTATTATCATGACATTAACCTATAAAAATAGGCGTATCACGAGGCCCTTTCGTCTCGCGCGTTTCGGTGATGACGGTGAAAACCTCTGACACATGCAGCTCCCGGAGACGGTCACAGCTTGTCTGTAAGCGGATGCCGGGAGCAGACAAGCCCGTCAGGGCGCGTCAGCGGGTGTTGGCGGGTGTCGGGGCTGGCTTAACTATGCGGCATCAGAGCAGATTGTACTGAGAGTGCACCATATGCGGTGTGAAATACCGCACAGATGCGTAAGGAGAAAATACCGCATCAGGAAATTGTAAGCGTTAATATTTTGTTAAAATTCGCGTTAAATTTTTGTTAAATCAGCTCATTTTTTAACCAATAGGCCGAAATCGGCAAAATCCCTTATAAATCAAAAGAATAGACCGAGATAGGGTTGAGTGTTGTTCCAGTTTGGAACAAGAGTCCACTATTAAAGAACGTGGACTCCAACGTCAAAGGGCGAAAAACCGTCTATCAGGGCGATGGCCCACTACGTGAACCATCACCCTAATCAAGTTTTTTGGGGTCGAGGTGCCGTAAAGCACTAAATCGGAACCCTAAAGGGAGCCCCCGATTTAGAGCTTGACGGGGAAAGCCGGCGAACGTGGCGAGAAAGGAAGGGAAGAAAGCGAAAGGAGCGGGCGCTAGGGCGCTGGCAAGTGTAGCGGTCACGCTGCGCGTAACCACCACACCCGCCGCGCTTAATGCGCCGCTACAGGGCGCGTCCATTCGCCATTCAGGCTGCGCAACTGTTGGGAAGGGCGATCGGTGCGGGCCTCTTCGCTATTACGCCAGCTGGCGAAAGGGGGATGTGCTGCAAGGCGATTAAGTTGGGTAACGCCAGGGTTTTCCCAGTCACGACGTTGTAAAACGACGGCCAGTGAATTGTAATACGACTCACTATA
